# Developing an initial programme theory for a model of social care in prisons and on release (empowered together): A realist synthesis approach

**DOI:** 10.1177/00258024241264762

**Published:** 2024-07-25

**Authors:** Deborah Buck, Lee D Mulligan, Charlotte Lennox, Jana Bowden, Matilda Minchin, Lowenna Kemp, Lucy Devine, Joshua Southworth, Falaq Ghafur, Catherine Robinson, Andrew Shepherd, Jennifer J Shaw, Katrina Forsyth

**Affiliations:** 1 Social Care and Society, 5292 University of Manchester, Manchester, UK; 2 Division of Psychology and Mental Health, 5292 University of Manchester, Manchester, UK; 3 1343 Pennine Care NHS Foundation Trust, Ashton-under-Lyne, UK; 4 5292 University of Manchester, Manchester, UK; 5 9022 Greater Manchester Mental Health NHS Foundation Trust, Prestwich, UK

**Keywords:** Adult offender, human rights, prison, older adult, social care, social justice

## Abstract

Many people are living in prison with a range of social care needs, for example, requiring support with washing, eating, getting around safely, and/or maintaining relationships. However, social care for this vulnerable group is generally inadequate. There is uncertainty and confusion about who is legally responsible for this and how it can best be provided, and a lack of integration with healthcare. We used realist-informed approaches to develop an initial programme theory (IPT) for identifying/assessing social care needs of, and providing care to, male adults in prison and on release. IPT development was an iterative process involving (a) an initial scoping of the international prison literature; (b) scoping prison and community social care policy documents and guidelines; (c) full systematic search of the international prison social care literature; (d) insights from the community social care literature; (e) stakeholder workshops. Information from 189 documents/sources and stakeholder feedback informed the IPT, which recommended that models of prison social care should be: trauma-informed; well integrated with health, criminal justice, third-sector services and families; and person-centred involving service-users in all aspects including co-production of care plans, goals, and staff training/awareness programmes. Our IPT provides an initial gold standard model for social care provision for people in prison and on release. The model, named Empowered Together, will be evaluated in a future trial and will be of interest to those working in the criminal justice system, care providers and commissioners, local authorities, housing authorities, voluntary groups, and service-users and their families.

## Introduction

There is mounting recognition that social care needs in and on release from prison are poorly identified and that when they are detected they are often inadequately met.^1–5^ Some people living in prison struggle to dress/bathe, get to the toilet, collect meals, or engage in rehabilitative activities, and some have inadequate help with incontinence and personal hygiene.^3,6–8^ This is a universal problem^9^ and provision largely remains fragmented with many people in prison left underserved.^9,10^

Moreover, given the increasing number of older people living in prison, the level of social care needs in this setting is increasing not only in the UK but in most high-income countries.^11,12^

According to international policy, care for people living in prison should equate to that in the community and there should be continuity of care upon release.^11,13,14^ However, social care in prison has been described as inconsistent or in some cases non-existent, exacerbated by unclear lines of responsibility.^11,15^ In England, before the 2014 Care Act^16^ it was unclear where responsibility lay for meeting social care needs in prison. This meant inadequate support or unmet needs,^17^ for example, people with mobility problems unable to shower regularly^18^; those with incontinence not being provided with pads/clean bedding.^8^ The Care Act clarified that responsibility for provision lies with local authorities (LAs), but there is no clear guidance on how services should be delivered, leaving this at the discretion of individual LAs.^19^ Adequacy of provision varies considerably between and across prisons and LAs, and no overarching model of best practice has been identified.^19^ It is unclear how much the situation has improved since the Care Act's inception and there is evidence of ongoing failings.^6^

There are examples of good social care practice in prison settings including sociable activities to overcome isolation of older adults in prison^20^; ‘light work’ activities for this group^5^; and well-supported/trained peer supporter systems.^10,19,21^ While these initiatives are encouraging, there is no consistency or guarantee of equivalence of care for people in prison compared to people living in the community, with inspectors reporting a ‘mixed picture’ despite the Care Act.^10^ Advocates are calling for people living in prison to have access to the same level of care as their community-dwelling counterparts,^4,20,22^ thereby meeting human rights obligations.^4,8^

Considering the insufficient guidance and inconsistent provision of social care in prisons, we aimed to develop an initial programme theory (IPT) and model for identifying, assessing, and providing for these needs (for individuals meeting Care Act eligibility criteria), with a view to evaluating in a future trial.

## Methods

### Realist approach and synthesis

Systematic literature reviews are often the first ports of call when informing policy or evidence-based guidelines.^23^ Our preliminary investigations found an absence of systematic literature reviews on provision of social care in prison and on release, however, and traditional systematic reviews do not lend themselves to learning from alternative settings or exploring what works, for whom, and why.^24^ Therefore, a realist approach and synthesis were adopted to explore the literature in prison and community settings.

Realist synthesis is an iterative, qualitative, theory-driven approach to synthesising qualitative, quantitative and/or mixed-methods data and is appropriate when considering complex interventions across different settings.^25,26^ It brings together evidence and insights from multiple sources, promotes stakeholder engagement, and optimises learning across policy, disciplinary and organisational domains.^26^ Programme theories are sets of assumptions about how and why a programme/intervention contributes to specific outcomes.^27–29^ A key concept is the context-mechanism-outcome (CMO) configuration: context is the ‘background environment’, and mechanisms are resources created by interventions/programmes, and people's response to them.^30^ Realist synthesis can explore insights from a diverse range of material including policy documents/guidelines/grey literature whereby insights may include authors’ interpretations/recommendations, and helps us to understand how interventions might work and why (rather than simply ‘does it work?’).^30–32^

As recommended by the RAMESES (Realist And MEta-narrative Evidence Syntheses: Evolving Standards) group, we adopted an abductive reasoning approach: information gathering and synthesis were iterative processes involving continual reviewing/refining to facilitate and fine-tune our evolving IPT.^28,30^

### Research questions

- What works for social care in prison and on release, in what circumstances, and why?
- What works for social care in the wider community, in what circumstances, and how can this be translated into prison settings?

### Process

**Figure 1.**
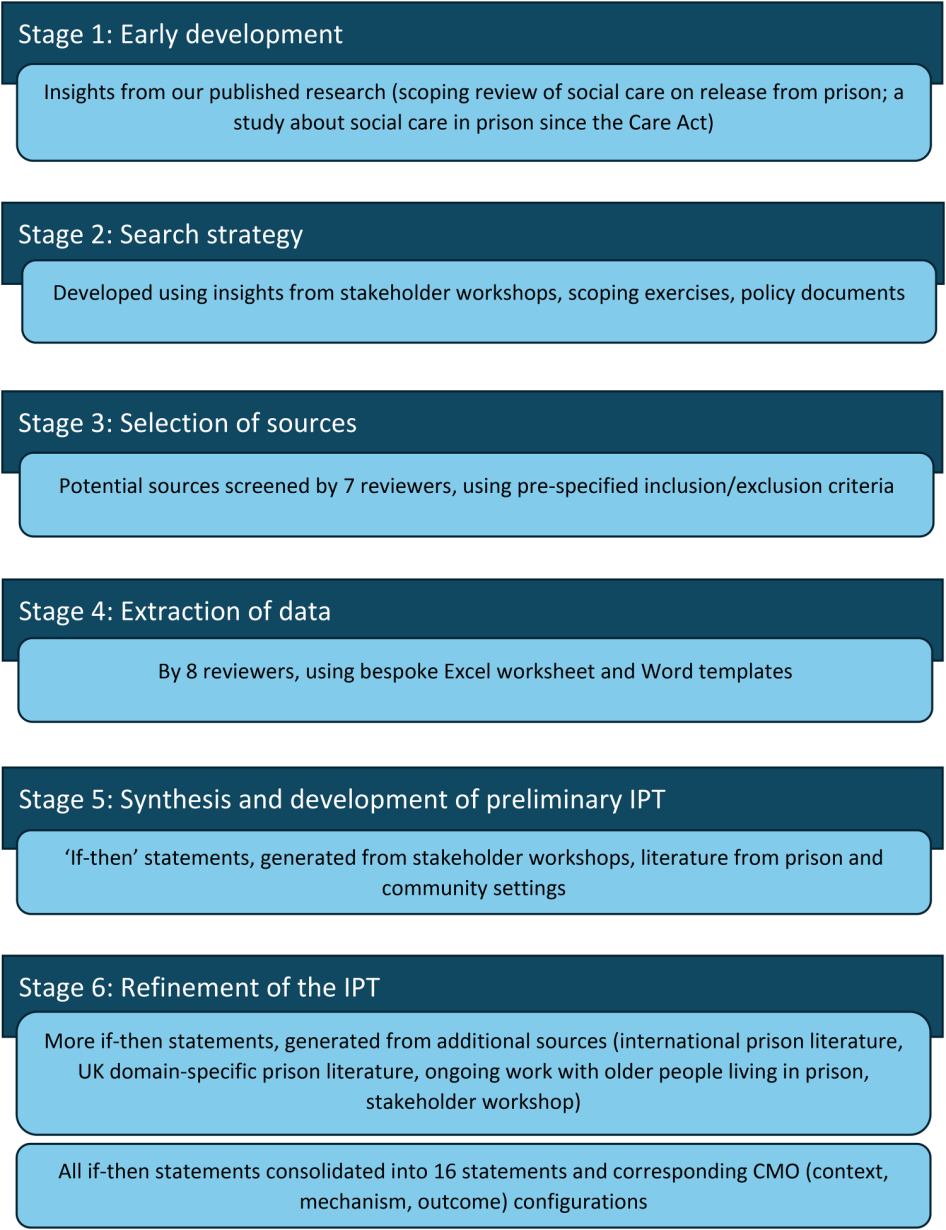
Multi-staged process for developing and refining the IPT using a realist synthesis approach.

The overall process for developing and refining the IPT, including development of the search strategy, was multi-staged. Each of these stages is summarised in Figure 1 and described in more detail below.

#### Stage 1: early development of IPT

We began developing our IPT based on findings from our previous research including a scoping review of social care on release from prison^33^ and a study about social care in prison since the Care Act.^19^ These provided an initial structure for our IPT, focussing on four aspects of social care: identification, assessment, provision in prison, provision on release.

#### Stage 2: literature search strategy/process

In line with the realist approach,^30–32^ the search process involved several stages to inform, develop and refine the search strategy before conducting a full systematic search. This included stakeholder workshops, scoping exercises, and examination of key policy documents. We also examined specific aspects of the literature identified as important by stakeholders, namely literature concerning strengths-based assessment approaches and community good practice. Full details of the overall process are included in Table 1. Lists of the search terms used are provided in Supplementary Table S1. Snowballing methods^34^ were used to identify additional sources.

**Table 1.** Iterative process for developing initial if-then statements and preliminary IPT framework, and refining IPT framework.

Stage	Date	Aim/method
1. First online stakeholder workshop	February 2022	Discuss priority areas to inform search strategy and Initial if-then statements. Focus: prison settings. Two-hour workshop with social care (SC) and prison staff.
2. Second online stakeholder workshop	March 2022	Discuss what can be learnt from community settings about identification/assessment of SC needs; how needs are met; how this might be applied to prisons. Two-hour workshop with prison and community SC staff and academics.
3. Initial scoping exercise	April 2022	Scope international literature on SC in prison to inform search strategy and initial if-then statements. Used PubMed search engine.
4. Main search: full systematic database search	April 2022; updated November 2023	Systematic search of literature on SC in prisons. Used Embase, Medline, PsycINFO, Social Policy & Practice, Criminal Justice Abstracts electronic databases.^a^
5. Recommendations from policy documents and guidelines on SC	April 2022	Search for/data extraction from UK policy documents/guidelines about SC to inform initial if-then statements. Used Google search engine.
6. Reviews of SC in the community	May 2022	Search for/data extraction from systematic literature reviews of SC in UK community settings to inform initial if-then statements. Used PubMed search engine.^a^
7. Search on strengths and assets-based approaches	May 2022	Search for/data extraction from examples of strengths and assets-based approaches to SC (as suggested during second workshop) to inform initial if-then statements. Used Google search engine.^a^
8. Search on good practice in the community	May 2022	Search for/data extraction from examples of ‘good practice’ SC in the community to inform initial if-then statements. Used Google search engine.^a^
9. Consulting our ongoing work	June 2022	Obtain insights from data already extracted as part of parallel work on health and social care specifically for older adults in prison (minimises duplication of effort), to generate additional if-then statements.
10. Third online stakeholder workshop	June 2022	Review the IPT developed to date. Two-hour workshop with members of our experts by experience (EBE) women's group. Group members emphasised need for trauma-informed approaches.
11. Search for trauma-informed approaches	October 2022	Search for trauma-informed approach to SC (as recommended during third stakeholder workshop). Used Google search engine. Extracted information about guidance for/benefits of this approach, to inform if-then statements/CMOs.
12. Fourth online stakeholder workshop	November 2022	Final ratification of IPT. Two-hour workshop with members of our Experts by Experience group.

^a^ Search strategy provided in Supplementary Table S1

#### Stage 3: screening/selection of literature

**Figure 2.**
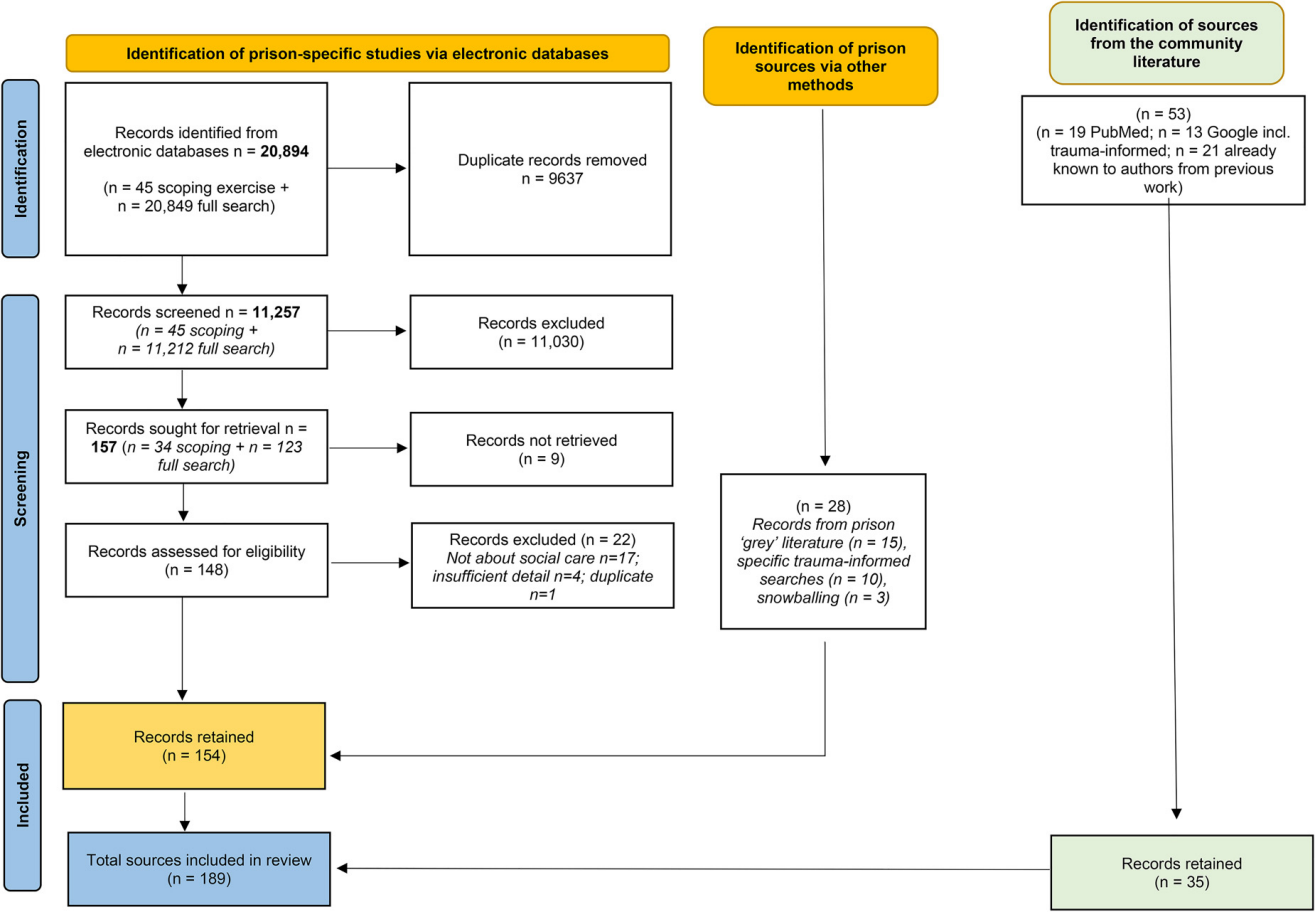
PRISMA flow diagram.

Articles were screened by abstract/title by seven reviewers using pre-specified inclusion/exclusion criteria (Supplementary Table S2). In total, 20,975 records were retrieved and 189 were retained. The PRISMA (Preferred Reporting Items for Systematic Reviews and Meta-Analyses) (flow diagram (Figure 2) displays full details of the sources and numbers of records retrieved/retained.

#### Stage 4: data extraction from literature

Data extraction was conducted by eight reviewers using bespoke Excel worksheet and Word templates. Information extracted included the population/sub-group, stages reported (e.g., identification/assessment/provision/release), type of social care, findings, examples/facilitators of good practice, and author recommendations. Data extraction templates are provided in Supplementary Table S3.

#### Stage 5: synthesis and development of the preliminary IPT

**Figure 3.**
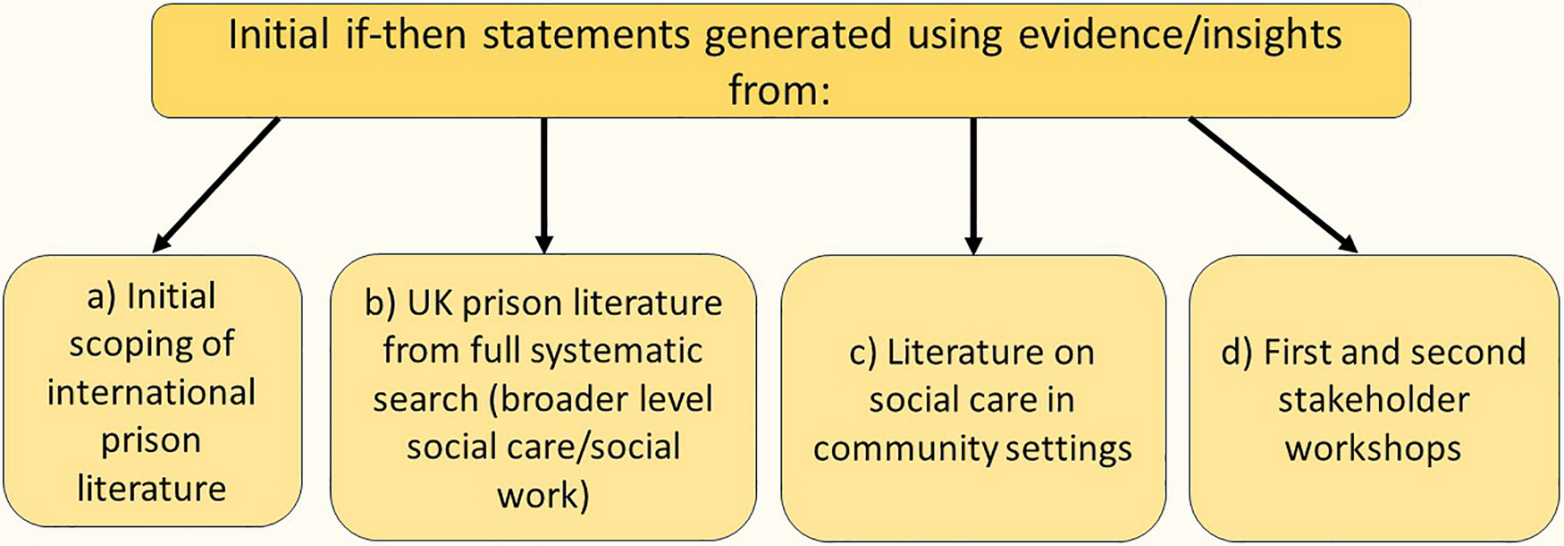
Developing the preliminary IPT - summary of the research process.

The preliminary framework for the IPT (Supplementary Figure S1) was informed by if-then statements which were generated from a variety of sources including stakeholder workshops and literature from prison and community settings (full details are provided in Figure 3). If–then statements help structure ideas and identify how an intervention might be linked to outcomes.^25^ Insights for these ‘scenarios’ can be gleaned from any section of the document. Generation of initial statements involved theorising how particular ‘resources’ (e.g., life skills training) may result in particular outcomes (e.g., successful reintegration). The statements were organised by theme into a single word document. This ‘working’ document was refined over several iterative steps through team discussions and updated during subsequent stages of IPT refinement (see below). The final version contains all statements, CMOs, and ‘nuggets’ of information,^29^ together with sources/references, and forms Supplementary File S1.

#### Stage 6: refinement of the IPT

**Figure 4.**
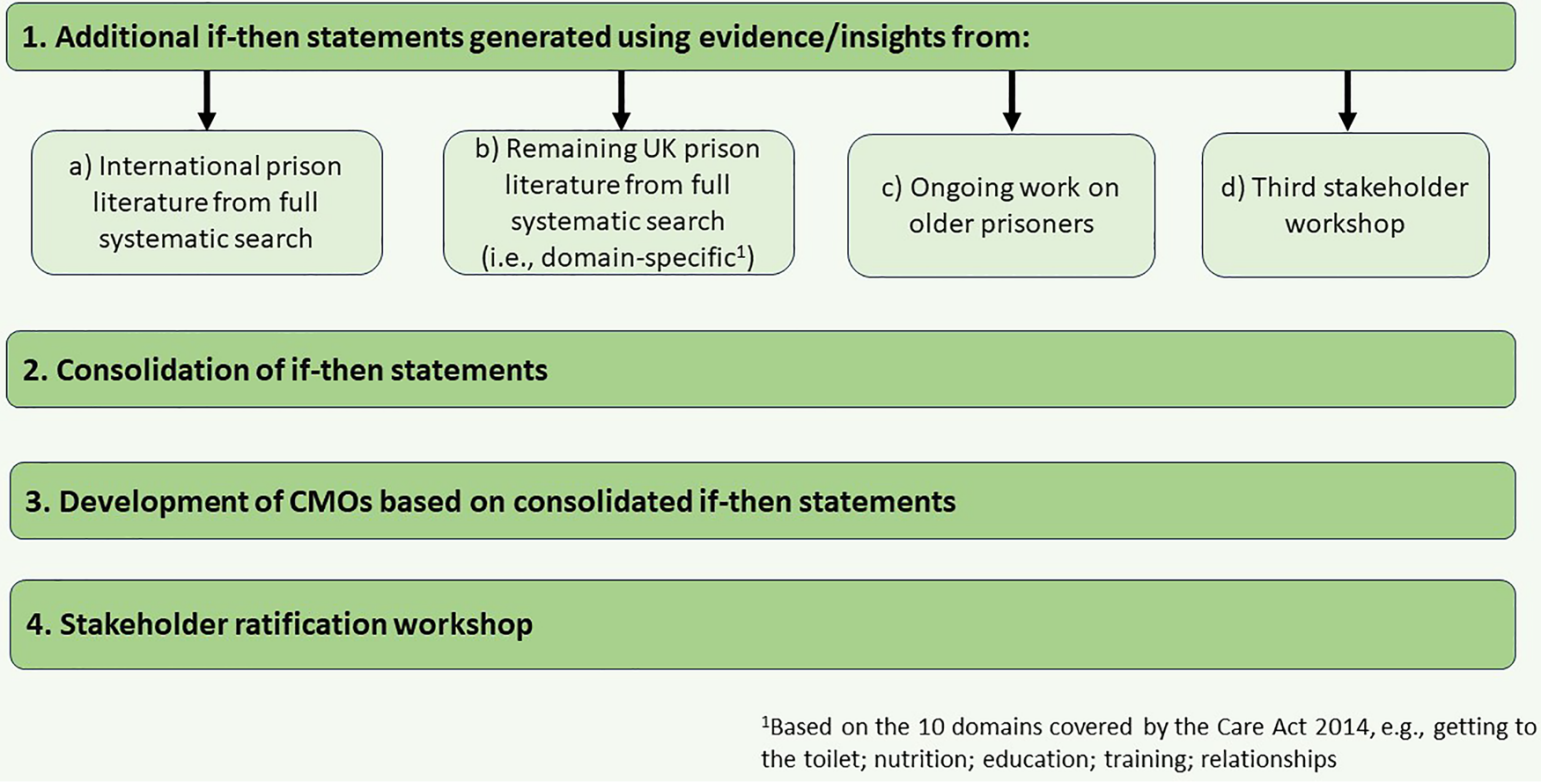
Refining the IPT - summary of the research process.

**Figure 5.**
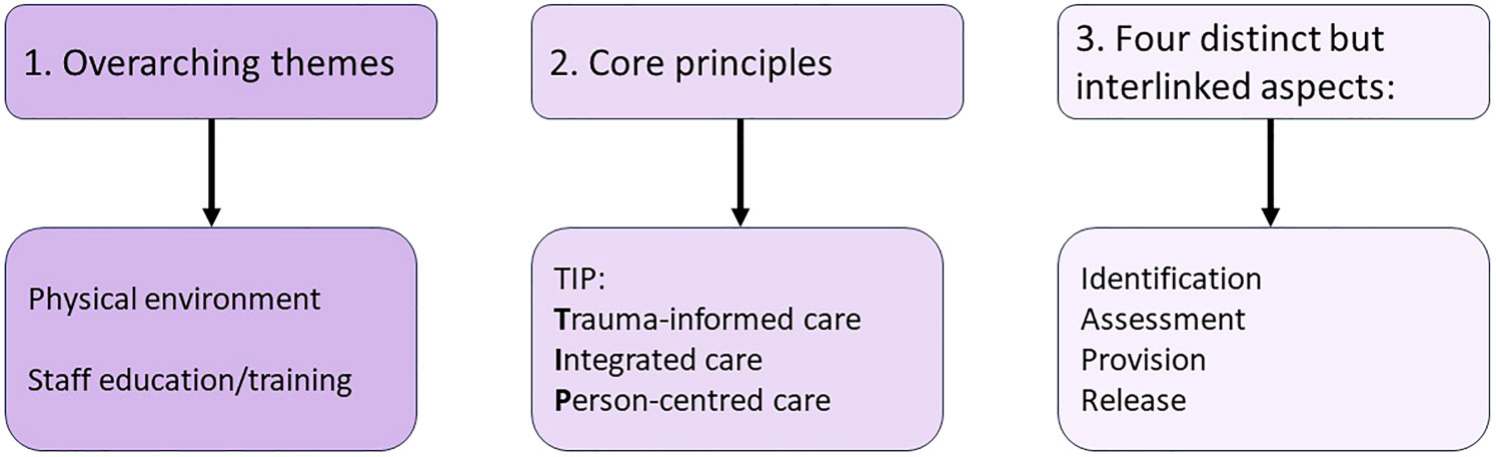
Three categories of CMO guiding the IPT.

Further if-then statements were generated based on additional sources as outlined in Figure 4. In total, 263 if-then statements were generated alongside 245 additional ‘nuggets’ of information (Supplementary File S1). Together with the initial statements, these were reformulated into 16 consolidated statements over several iterative steps to produce richer, more detailed accounts while minimising overlap/repetition.^35^ We used these 16 statements to develop 16 corresponding CMO configurations, theorising about expected responses to proposed interventions/resources. The theoretical elements were based on evidence/insights from the literature and reviewers’ hypotheses and were discussed/refined by the team as part of the iterative process, with mechanisms subdivided into ‘resource’ and ‘response/reasoning’ components. These help to explain how resources work to change individual or group reactions/reasoning which achieves the outcomes.^36,37^ After several further phases of CMO development, including creative mind-mapping sessions with the review team and reflecting further on the data, we distinguished three categories of CMO as outlined in Figure 5, and 11 CMOs concerning identification, assessment, provision, and release which were combined into four consolidated CMOs.

## Results

This section presents a narrative summary of the four consolidated CMOs, which are outlined in Table 2 and visually in Supplementary Figure S2. Thus, this section encompasses a description of the model of social care that our expert-by-experience group has named ‘Empowered Together’. An extended version of the findings is available (Supplementary File S2). The content of the CMOs is based on evidence and insights from sources as described In the Methods. The complete list of if-then statements, CMOs, and ‘nuggets’ of information are included in Supplementary File S1.

**Table 2.** Context, mechanism, outcome (CMO) configurations.

Context	Mechanism (resource)	Mechanism (response)	Outcomes
CMO1: Identification of social care needs			
Identification of people living in prison with social care (SC) needs is not always conducted systematically on entry to prison, over the course of the sentence, or prior to release. SC needs are not well understood among many prison staff, and there is confusion about staff's responsibilities. Some people in prison are reluctant to acknowledge care needs, some do not have the capacity, and some are unaware of their entitlements. The high turnover of the prison population and unpredictable nature of transitions between prisons makes SC provision and continuity of care additionally challenging.	A standardised, validated person-centred screening tool should be developed, to be administered by SC practitioners trained to use it using a trauma-informed approach. Screening should include identifying problems posed by the physical environment. Active case finding should be promoted, including when individuals move wing or prison; need clarity for staff about roles/responsibilities. People in prison, families, and staff should be educated to be aware of, anticipate, and be sympathetic to SC needs and individuals’ right to receive support. SC practitioners should receive prison-specific training. Easy-read posters/leaflets about SC should be available to everyone. Need accessible self-referral systems and advocacy for those lacking capacity.	People in prison: empowerment; willing to acknowledge problems; maintain dignity; feel valued, validated; trust; feel safe. Staff: empathy, compassion, respect; confidence in role.	People in prison: needs identified in a sensitive, timely manner; less risk of needs being overlooked; enhanced preventative care; increased awareness; more self-referrals. Staff: Greater awareness among prison staff of emerging/changing needs; confidence and clarity in role; rapport. SC practitioners more confident about working in prison settings.
CMO2: Assessment of social care needs/care-planning			
On entry to prison, some individuals are seen by staff who are unaware of SC issues or how to talk with those in need. SC needs change over time but no systematic follow-up procedures to monitor needs. Some people in prison reticent to acknowledge SC needs for fear of appearing vulnerable. Trauma survivors at risk re-traumatisation by the assessment experience/process.	Qualified SC practitioners trained in trauma-informed approaches in prison settings should administer strength-based assessments where individuals are encouraged to discuss own priorities. Care plans should be co-designed to be flexible in anticipation of changing needs and take account of the physical environment; they should be available to relevant staff and accompany the individual if they are moved to another location. MDTs should be involved in person-centred care planning; team should include a social care lead to coordinate referrals, etc. but should be responsibility of everyone including prison staff to ensure plans are fulfilled. Follow-up assessments should occur at least twice yearly, with clear review dates in response to changing needs. Those who lack capacity should receive advocacy support.	People in prison: trust staff more; feel safer; more engaged with assessments/care plans; feel more valued; a sense of hope. Staff: more empathetic in their approach; more confident and organised in their role.	More holistic assessments promoting equality and social inclusion; tailored care plans more responsive to changing needs; less risk of re-traumatisation; greater equivalence of care; improved relationships between staff and people in prison, and between prison and other staff.
CMO3: Provision of SC in prison			
General aspects of SC in prison include: activities of daily living (ADLs) which some people in prison need help with (e.g., bathing, toileting, mobility); peer support (not always appropriate); access to purposeful activity (lack of suitable activities for people in prison with SC needs); maintaining relationships (many find this difficult, especially people in prison with SC needs alongside other problems such as learning difficulties or mental health problems).	Social care leads should be nominated to coordinate care and ensure there is no gap in provision if people move prison. There should be appropriate personal support, adaptations, and equipment available to meet the ADL needs of people in prison. Formal peer supporter systems should be established which include training, job descriptions, and ongoing support for peer supporters, and safeguarding for peer supporters and those receiving support. Tailored work, education, and other activities including day-care support should be codesigned with voluntary organisations and people in prison and ensure ease of access. Trauma-informed arrangements should be in place which (a) allow people in prison adequate contact with family and (b) provide accessible facilities to enable social interaction and development of positive relationships with other people living in prison.	Care receivers: feel connected, valued, motivated; a sense of hope, purpose, mastery, and autonomy while maintaining dignity. More trust and feel safer. Peer supporters: feel empathy; a sense of purpose; pride.	People in prison receive more appropriate and timely support; continuity of care; improved human rights; social inclusion; equal access to purposeful activity; new skills; improved prospects; less isolated; more autonomy; better relationships with other people living in prison, and family/friends. Peer supporters benefit from new skills, increased confidence, prospects, and self-esteem. Less strain on prison staff. Improved relationships between staff and people in prison.
CMO4: Provision of SC on release			
Ex-offenders face many barriers to successful resettlement on release from prison, including nowhere to stay, no training or employment lined up, little money or clothing, no medication or immediate access to services. Exacerbated when release day is Friday. Employers’ reluctance to employ ex-offenders. Significantly increases risk of: homelessness; declining physical & mental well-being; re-offending.	Release planning should begin asap and involve assessments including housing needs. Co-designed pre-release courses should be run by specially trained staff and be trauma-informed with tailored content for people with SC needs, taking into account of other issues such as LLDs. People in prison should be involved in the planning and be enabled to learn work and life skills. Personalised guidance and care packages should be provided, including suitable housing. Trauma-informed job placements should be established. There should be ‘nodal points’ for trauma-informed probation and aftercare services. Release days should be Monday to Thursday when access to services is more likely; arrangements should be in place for regular follow-ups and accessible self-referral systems.	People in prison: respond with positivity; feel motivated; sense of dignity, pride, hope; trust; safety. Employers: feel compassion; sense of benevolence for disadvantaged people; pride in company/organisation	Reduced anxieties prior to release; greater equality and social inclusion; resettlement more successful by improving life & work skills and better meeting housing and other social care needs, and ensuring continuity of care; greater success in securing/retaining work; less homelessness and re-offending; wider societal benefits.

### Identifying social care needs

Before social care needs can be addressed they need to be identified on arrival and, for emerging needs, at any point during an individual's stay in prison.

A key resource needed to ensure accurate and timely identification of needs is a standardised but flexible, person-centred screening tool capable of detecting ‘harder to see’ needs. This should include open-ended questions, enabling/encouraging individuals to acknowledge difficulties they have with mobility, self-care, relationships, or other elements of social care, with social care practitioners trained to screen in a sensitive, trauma-informed way. The identification process should include screening for mental health problems, learning difficulties, dementia, mild cognitive impairment, and trauma alongside general health and social care needs.

Active case finding is fundamental to ensure social care needs are identified, particularly those that may not be apparent on arrival from the community or when individuals move to a different prison. This should involve repeating the formal identification process twice yearly after arrival and enabling suitably trained staff to proactively talk to people, asking questions about their ability to get around safely, take care of themselves, and participate in activities, with a robust route into assessment. This should involve liaison between care staff and prison officers who may identify/refer people with additional support needs if these become apparent.

The identification of social care needs must happen within an integrated care system which includes clarity for staff about respective roles/responsibilities. Awareness of issues relating to social care is vital for this to work. While all people living in prison, their families, and staff should be educated about social care issues, staff should receive formal training to recognise/understand these needs, drawing on the tenets of equality, diversity, and inclusion. Training programmes should be co-designed with people with lived experience of prison and voluntary organisations, further promoting person-centredness and ensuring pertinent issues are addressed. While some social workers receive training around social work in prisons, many do not feel suitably equipped or confident to work in this setting or with particular groups such as people with substance misuse problems. Well-advertised, accessible self-referral options should be available together with user-friendly informational posters/leaflets, such as ‘The Care Act and You’ leaflet, with the option of advocacy to ensure the information is understood by the individual or, where necessary, make decisions on their behalf.

### Assessing social care needs

Individuals should be empowered to be actively involved in their assessments and in co-designing care plans, which must be timely and take the limitations of the physical environment into account. Care plans should be available to relevant staff and accompany the individual if they are moved to another location. Advocacy should be offered to people with insufficient capacity to meaningfully participate in their assessments/care plans. Advocates could be informal and internal to the prison (peers, prison staff, healthcare staff) with the person's consent, or formal independent advocacy services including LAs or other staff who must be approved by the LA (e.g., the third sector). All staff should be educated about trauma-informed approaches. The assessment process itself will be traumatising for some individuals, so formal assessments should be conducted by suitably qualified practitioners.

Strengths and assets-based approaches to assessment and care-planning focus on people's capabilities, explore help available from wider support networks, and take account of issues of importance to the individual. Care needs change over time, therefore regular follow-up assessments should be conducted, with changes to care plans being jointly agreed. It is important that everyone has user-friendly information about social care rights and the option to self-refer in response to changing needs.

### Provision of social care/support

To optimise social care in prisons it is vital that collaborative, integrated working is implemented whereby prisons, LAs, healthcare, social care, and voluntary organisations develop joint working arrangements with multidisciplinary teams (MDTs), co-designed goals, shared aims, and agreements on information sharing. Pivotal to this is the nomination of a social care lead in each prison to facilitate the co-ordination of care. Social care leads should be embedded within the integrated care system to coordinate referrals and ensure continuity of care if people move prison. MDTs should be involved in care-planning with the social care lead, but it is crucial that there is clarity regarding roles and responsibilities and that all stakeholders are accountable for implementing these plans, which could be expedited by co-designed memorandums of understanding.

Services should be co-designed with people who have social care needs and experience of living in prison. Tailoring to individual needs, this should include safe access to purposeful activities and fully integrated, meaningful day-care support where appropriate. Support should be available to help people build/maintain relationships with family and peers.

Peer support systems are seen as a major resource. It is evident, however, that peer supporters must be suitably assessed, trained, safeguarded, and supported themselves. Where possible, the peer supporter and support receiver should be matched to take account of cultural, religious, communication and support needs. Training and support for peers could involve third-sector organisations (such as RECOOP), while group peer mentoring programmes could be run by ex-offenders with social care needs. A professional could be employed to support peer supporters who could be encouraged to obtain recognised qualifications. Peer supporters should not be involved in providing personal care.

Individuals should be supported to maintain contact with family/friends, and arrangements made to encourage family visits. This is particularly important for certain groups such as people with learning difficulties. The duration/frequency of visits may be more than the standard amount if this is what individuals want/need to maintain social networks. This could include online visits/video visitations, which should not be a replacement for in-person visits. It is important to ensure facilities within prison are accessible to enable social interaction and development of positive relationships with peers. Accessible activities include tailored clubs and buddying systems. Positive staff relationships are important, particularly for those with no other contacts.

### Social care on release from prison

People living in prison should be offered tailored pre-release planning and courses, and planning should begin as early as possible, with the individual's engagement, to enable assessment of predicted needs, and covering, at the very least, housing, training/education, employment, food provision, and social and healthcare. People should be enabled to maintain or learn life skills, with other issues such as language and learning disorders (LLDs) being considered. Courses should be co-designed, trauma-informed, and run by staff who have been specifically trained. Employment training should focus on basic work/life skills. Employment placement programmes should ideally be developed with input from employment specialists. Individuals should be provided with personalised guidance notes prior to release, and a care package (if applicable) and personalised pathway document upon release. A single point of contact should be established for those returning to the community, providing trauma-informed probation, counselling, and aftercare services, continuing-care packages, and joined-up support including suitable housing, employment, and access to benefits. There should be arrangements for regular follow-up and ongoing support, and self-referral options to address changing needs.

## Discussion

### Summary of findings

Our IPT proposes that social care in and on release from prison should be trauma-informed, integrated, and person-centred (TIP). To effectively identify social care needs there needs to be a standardised but flexible systematic screening tool, alongside active case finding and training to increase awareness. Assessments should be strengths-based resulting in co-produced care plans with advocacy provided where needed. Care should be coordinated by a social care lead. Peer support should be provided where appropriate, by trained, well-supported and supervised peers. Early coproduced pre-release courses should be provided, alongside personalised guidance notes and an identified single point of contact should be provided for individuals with social care needs on release.

### Comparisons to the literature

TIP principles are increasingly encouraged in community settings,^38,39^ and are becoming relatively well-established in the criminal justice systems of the UK and USA.^40^ However, in the UK this applies only to high secure male prison estates.^40^ Our programme theory/model focuses on social care in and on release from prison and applies to all categories of male prisons. This is important because people living in prison are among the most vulnerable, marginalised and excluded in society.^41^ Providing TIP care/support to this group will help reduce inequalities, improve well-being, prevent/delay further social care needs, and improve post-release outcomes.

Reintegration is difficult for most people on release from prison who face challenges such as limited family support, stigma, no employment links, and no stable housing.^42,43^ These challenges tend to be amplified for people with social care needs. Our IPT offers solutions to these challenges, including skills training, co-designed pre-release courses, and personalised guidance and care packages,^4,44–46^ set within TIP approaches.

### Strengths and limitations

To the best of our knowledge, this is the first realist-informed synthesis to determine how the social care needs of men in prison and on release may be best identified, assessed, and met. Whilst the current study included international literature, sources were restricted to English language: it is possible that valuable insights have been omitted. We acknowledge that the framework is predominantly England-focused but believe many other jurisdictions could take some valuable insights from this. Financial implications were not considered, and while some new or improved services would entail financial input, it is likely that the longer-term benefits from preventative care and more successful reintegration into the community would negate this financial cost. Our IPT does not cover people living in the female prison estate, as it is widely held that many of the social care needs of this group differ to those in male prisons. However, we are currently undertaking a study focusing specifically on this group.

### Implications for policy and practice

The need to formalise agreements between LAs, healthcare providers and prison staff for the provision of social care in prison is evident. The Care Act stipulates that this should be in the form of a memorandum of understanding; however, such formal understandings are lacking or poorly implemented. This is the first step for improving social care provision in prisons. The need for a designated prison-based social care lead to coordinate service provision is a further aspect of the management structure that requires implementing to enable the development of effective, integrated prison health and social care.^4,10,47^ Cultural change is also required to ensure that the principles of trauma-informed, integrated, person-centred care are embedded within health, social care, and custodial organisations.^9,48^ More pragmatically, thorough screening and active case finding is required to ensure individuals with social care needs are not missed.^19^ Strengths-based assessments, appropriate peer support, and coproduced care plans should also be implemented. Finally, to prepare for release, early coproduced pre-release courses, personalised guidance and a single point of contact should also be provided.

Discernible barriers to be addressed include lack of awareness among staff and people living in prison,^49^ challenges in training entire workforces characterised by high turnover,^42^ silo-working (stakeholder workshop, February 2022), and uncertainty around legal responsibilities, roles and accountability.^50,51^ How to eventually achieve a ‘gold standard’ will vary between prisons and across LAs, depending on current systems and presence/absence of existing agreements, and the characteristics of individual prison estates. While legislation has clarified who is responsible for social care in England's prisons, provision remains inconsistent and until now there was no proposed programme theory aiming to address this. A strength of our IPT is that it is accompanied by examples of how this may be achieved.

### Future research

There is a dearth of empirical evidence relating to models of social care in prison since the introduction of the 2014 Care Act. We have developed an IPT and logic model for social care in and on release from prison, based on available information. The next steps are to implement this systems-level ‘Empowered Together’ model and conduct a feasibility study. The model will need further refinement and customisation for different prison settings before a full evaluation can be conducted.

## Conclusion

In conclusion, our IPT provides a sound initial framework indicating how social care in prisons can be developed to achieve equivalence of care while preserving dignity, preventing/delaying deterioration, improving prospects and in the long-term improving outcomes and benefiting the wider community.

## Supplemental Material

sj-docx-1-msl-10.1177_00258024241264762 - Supplemental material for Developing an initial programme theory for a model of social care in prisons and on release (empowered together): A realist synthesis approach Supplemental material, sj-docx-1-msl-10.1177_00258024241264762 for Developing an initial programme theory for a model of social care in prisons and on release (empowered together): A realist synthesis approach by Deborah Buck, Lee D Mulligan, Charlotte Lennox, Jana Bowden, Matilda Minchin, Lowenna Kemp, Lucy Devine, Joshua Southworth, Falaq Ghafur, Catherine Robinson, Andrew Shepherd, Jennifer J Shaw and Katrina Forsyth in Medicine, Science and the Law

sj-pdf-2-msl-10.1177_00258024241264762 - Supplemental material for Developing an initial programme theory for a model of social care in prisons and on release (empowered together): A realist synthesis approach Supplemental material, sj-pdf-2-msl-10.1177_00258024241264762 for Developing an initial programme theory for a model of social care in prisons and on release (empowered together): A realist synthesis approach by Deborah Buck, Lee D Mulligan, Charlotte Lennox, Jana Bowden, Matilda Minchin, Lowenna Kemp, Lucy Devine, Joshua Southworth, Falaq Ghafur, Catherine Robinson, Andrew Shepherd, Jennifer J Shaw and Katrina Forsyth in Medicine, Science and the Law

sj-docx-3-msl-10.1177_00258024241264762 - Supplemental material for Developing an initial programme theory for a model of social care in prisons and on release (empowered together): A realist synthesis approach Supplemental material, sj-docx-3-msl-10.1177_00258024241264762 for Developing an initial programme theory for a model of social care in prisons and on release (empowered together): A realist synthesis approach by Deborah Buck, Lee D Mulligan, Charlotte Lennox, Jana Bowden, Matilda Minchin, Lowenna Kemp, Lucy Devine, Joshua Southworth, Falaq Ghafur, Catherine Robinson, Andrew Shepherd, Jennifer J Shaw and Katrina Forsyth in Medicine, Science and the Law

sj-docx-4-msl-10.1177_00258024241264762 - Supplemental material for Developing an initial programme theory for a model of social care in prisons and on release (empowered together): A realist synthesis approach Supplemental material, sj-docx-4-msl-10.1177_00258024241264762 for Developing an initial programme theory for a model of social care in prisons and on release (empowered together): A realist synthesis approach by Deborah Buck, Lee D Mulligan, Charlotte Lennox, Jana Bowden, Matilda Minchin, Lowenna Kemp, Lucy Devine, Joshua Southworth, Falaq Ghafur, Catherine Robinson, Andrew Shepherd, Jennifer J Shaw and Katrina Forsyth in Medicine, Science and the Law

sj-docx-5-msl-10.1177_00258024241264762 - Supplemental material for Developing an initial programme theory for a model of social care in prisons and on release (empowered together): A realist synthesis approach Supplemental material, sj-docx-5-msl-10.1177_00258024241264762 for Developing an initial programme theory for a model of social care in prisons and on release (empowered together): A realist synthesis approach by Deborah Buck, Lee D Mulligan, Charlotte Lennox, Jana Bowden, Matilda Minchin, Lowenna Kemp, Lucy Devine, Joshua Southworth, Falaq Ghafur, Catherine Robinson, Andrew Shepherd, Jennifer J Shaw and Katrina Forsyth in Medicine, Science and the Law

sj-docx-6-msl-10.1177_00258024241264762 - Supplemental material for Developing an initial programme theory for a model of social care in prisons and on release (empowered together): A realist synthesis approach Supplemental material, sj-docx-6-msl-10.1177_00258024241264762 for Developing an initial programme theory for a model of social care in prisons and on release (empowered together): A realist synthesis approach by Deborah Buck, Lee D Mulligan, Charlotte Lennox, Jana Bowden, Matilda Minchin, Lowenna Kemp, Lucy Devine, Joshua Southworth, Falaq Ghafur, Catherine Robinson, Andrew Shepherd, Jennifer J Shaw and Katrina Forsyth in Medicine, Science and the Law

sj-docx-7-msl-10.1177_00258024241264762 - Supplemental material for Developing an initial programme theory for a model of social care in prisons and on release (empowered together): A realist synthesis approach Supplemental material, sj-docx-7-msl-10.1177_00258024241264762 for Developing an initial programme theory for a model of social care in prisons and on release (empowered together): A realist synthesis approach by Deborah Buck, Lee D Mulligan, Charlotte Lennox, Jana Bowden, Matilda Minchin, Lowenna Kemp, Lucy Devine, Joshua Southworth, Falaq Ghafur, Catherine Robinson, Andrew Shepherd, Jennifer J Shaw and Katrina Forsyth in Medicine, Science and the Law
