# Supplementary material for: Developing an initial programme theory for a model of social care in prisons and on release (empowered together): A realist synthesis approach
Source: Med Sci Law. 2024 Jul 25;65(3):194–206. doi: 10.1177/00258024241264762 (PMC12149453; doi:10.1177/00258024241264762)
Supplement: sj-docx-1-msl-10.1177_00258024241264762 - Supplemental material for Developing an initial programme theory for a model of social care in prisons and on release (empowered together): A realist synthesis approach [file sj-docx-1-msl-10.1177_00258024241264762.docx]

**Supplementary Figure S1: Preliminary IPT for model(s) of social care in prison and on release**

| **Context (C)**  **(wider)** | **Legal framework:**  Care Act; Disability Discrimination Act; Human Rights | | | |
| --- | --- | --- | --- | --- |
|  | **Policy:**  PSI: Prisoners assisting other prisoners; Information sharing agreements; Joint workforce plans; Joint funding and commissioning;  Alternatives to custody (compassionate release, secure nursing homes, ROTL, community sentencing); | | | |
|  | **Environment**:  Suitably adjusted prisons; specially adapted wings and cells | | | |
| **Context (C)**  **(prison)** | **Person-centred care:**  Equality, diversity, and inclusion; co-production/design | | | |
|  | **Integrated care**  **(key stakeholders = health, local authorities, CJS, third sector, peers, families/carers, service user):**  Co-production; protocols; MoU; SOPs; MDTs; continuous training; shared goals; awareness; co-location of staff; team work; good and clear communication; frailty register; shared IT systems ; single points of access; continuity of care | | | |
|  | **Local leads / Single points of access** | | | |
| **Mechanisms**  **(M)** | **Prevention of unmet social care needs and deterioration** | | | |
|  | **Identification**  Active case finding  Accessible self-referral  Frailty register  Day centres  OMIC | **Assessment and care planning**  Strength/asset-based approach  3 conversations  Standardisation | **Care and support**  Peer/buddy carers  Day centres/groups  Purposeful activity  Rehabilitation & enablement | **Release**  Early planning  Appropriate accommodation  Pre-release courses  Personalised guides  Continuity of care |
| **Outcomes**  **(O)** | **Criminogenic:** recidivism,  **Wellbeing:** QoL; reduced anxiety  **Social care:** needs identified; needs met; safety, independence | | | |
