# Supplementary material for: Developing an initial programme theory for a model of social care in prisons and on release (empowered together): A realist synthesis approach
Source: Med Sci Law. 2024 Jul 25;65(3):194–206. doi: 10.1177/00258024241264762 (PMC12149453; doi:10.1177/00258024241264762)
Supplement: sj-pdf-2-msl-10.1177_00258024241264762 - Supplemental material for Developing an initial programme theory for a model of social care in prisons and on release (empowered together): A realist synthesis approach [file sj-pdf-2-msl-10.1177_00258024241264762.pdf]

Supplementary Figure S2:

Overview of the IPT for social care (SC) in prisons and on release: overarching themes, core principles & interrelated stages

Core principles

- Trauma-informed
- Integrated care
- Person centered

Overarching themes

- Staff training & education
- Physical environment

Mechanisms (resources)	Outcomes
**Identification of social care needs**<br>Person-centered screening tools; user-friendly information; self-referral; advocacy; Screening for trauma; Awareness of roles; active case finding; Co-designed, trauma-informed training; Identifying potential barriers in physical environment	More needs identified; more self-referrals; prevention; staff awareness & confidence in role; rapport
**Assessment & care planning**<br>Strengths and assets based; co-designed personalised care plans; regular reviews; advocacy; Training in trauma-informed approaches for qualified SC practitioners; MDTs; Care co-ordination; Care plans take account of physical environment	Holistic assessments; less re-traumatisation; responsive care plans; less strain on staff
**Care & support in prison**<br>Accessible, co-designed meaningful activity; support to maintain relationships; Safeguarding; Care co-ordination; MDTs; formal peer supporter scheme; Co-designed staff training programmes; Adaptations/equipment to meet ADL needs; designated blocks	Appropriate support; social inclusion; equality; confidence; autonomy; well-being
**Care & support on release into the community**<br>Early, tailored release planning, guidance & care packages including employment & appropriate accommodation, clothing, equipment etc.; follow-ups; 'Nodal points' for trauma-informed probation & aftercare services; Co-designed pre-release courses	Life skills; less anxiety; successful re-entry; less recidivism; continuity; social inclusion
