# Supplementary material for: Developing an initial programme theory for a model of social care in prisons and on release (empowered together): A realist synthesis approach
Source: Med Sci Law. 2024 Jul 25;65(3):194–206. doi: 10.1177/00258024241264762 (PMC12149453; doi:10.1177/00258024241264762)
Supplement: sj-docx-3-msl-10.1177_00258024241264762 - Supplemental material for Developing an initial programme theory for a model of social care in prisons and on release (empowered together): A realist synthesis approach [file sj-docx-3-msl-10.1177_00258024241264762.docx]

**Supplementary Table S1: search strategies**

| **Community literature searches** | |
| --- | --- |
| **a) PubMed search for systematic reviews of social care in the community** (conducted on 4th May 2022 by DB) | |
| “social care” [title or abstract]  and  “UK” or “United Kingdom” or “England” | Restricted to ‘systematic reviews’ and years 2012 to 2022 |
| **b) Google** (search conducted in May 2022 by LD) | |
| Search terms | Comments |
| 1. “Social care" and "strengths and assets" | Searched following stakeholder feedback |
| 2. "Social care" and "three conversations model" and "prison" | Searched following stakeholder feedback |
| 3. "Social care" and "three conversations model" | To see if we could find anything useful from the wider community, i.e., not in prisons |
| 4. "Social care” and “adults in the community” and "England" and "examples of good practice" | To see if there were any examples of good practice of social care provision (or assessment) in England |
| **Prison literature searches** | |
| **a) PubMed search to inform initial scoping exercise** (conducted on 8 April 2022 by DB) | |
| Prisoner* or Offender* or Inmate*  AND  “Social Care” | Restricted to years 2012 to 2022 |
| b**) Full search of the prison social care literature** (conducted on 22 April 2022 by DB) | |
| OVID search string for Embase, Medline, PsycINFO, Social Policy & Practice  (ti = title, ab = abstracts)  ((Prison*.ti. or Prison*.ab.) OR (Offender*.ti. or Offender*.ab.) OR (Inmate*.ti. or Inmate*.ab.))  AND  ((Social Care.ti. or Social Care.ab.) OR (ADLs.ti. or ADLs.ab.) OR (Activities of daily living.ti. or Activities of daily living.ab.) OR (Family Relationships.ti. or Family Relationships.ab.) OR (Feeding.ti. or Feeding.ab.) OR (Eating.ti. or Eating.ab.) OR (Nutrition.ti. or Nutrition.ab.) OR (Personal Relationships.ti. or Personal Relationships.ab.) OR (Recreational Services.ti. or Recreational Services.ab.) OR (Recreational Facilities.ti. or Recreational Facilities.ab.) OR (Personal Care.ti. or Personal Care.ab.) OR (Personal hygiene.ti. or Personal hygiene.ab.) OR (Shower*.ti. or Shower*.ab.) OR (Bath*.ti. or Bath*.ab.) OR (Train*.ti. or Train*.ab.) OR (Educ*.ti. or Educ*.ab.) OR (Employ*.ti. or Employ*.ab.) OR (Work.ti. or Work.ab.) OR (Volunteer*.ti. or Volunteer*.ab.) OR (Caring Responsibilities.ti. or Caring Responsibilities.ab.) OR (Continen*.ti. or Continen*.ab.) OR (Incontin*.ti. or Incontin*.ab.) OR (Toilet Needs.ti. or Toilet Needs.ab.) OR (Carer For.ti. or Carer For.ab.) OR (Dressing.ti. or Dressing.ab.) OR (Getting Dressed.ti. or Getting Dressed.ab.))  Criminal Justice Abstracts search string  (TI = title, AB = abstracts)  (TI Prison* or AB Prison*) OR (TI Offend*. or AB Offend*) OR (TI Inmate* or AB Inmate*)  AND  (TI “Social Care” or AB “Social Care”) OR (TI ADLs or AB ADLs) OR (TI “Activities of daily living” or AB “Activities of daily living”) OR (TI “Family Relationships” or AB “Family Relationships”) OR (TI Feeding or AB Feeding) OR (TI Eating or AB Eating) OR (TI Nutrition or AB Nutrition) OR (TI “Personal Relationships” or AB “Personal Relationships”) OR (TI “Recreational Services” or AB “Recreational Services”) OR (TI “Recreational Facilities” or AB “Recreational Facilities”) OR (TI “Personal Care” or AB “Personal Care”) OR (TI “Personal hygiene” or AB “Personal hygiene”) OR (TI Shower* or AB Shower*) OR (TI Bath* or AB Bath*) OR (TI Train* or AB Train*) OR (IT Educ* or AB Educ*) OR (TI Employ* or AB Employ*) OR (TI Work or AB Work) OR (TI Volunteer* or AB Volunteer*) OR (TI “Caring Responsibilities” or AB “Caring Responsibilities”) OR (TI Continen* or AB Continen*) OR (TI Incontin* or AB Incontin*) OR (TI “Toilet Needs” or AB “Toilet Needs”) OR (TI Carer For or AB Carer For) OR (TI Dressing or AB Dressing) OR (TI “Getting Dressed” or AB “Getting Dressed”) | |
| **Community literature searches** | |
| **a) PubMed search for systematic reviews of social care in the community** (conducted on 4th May 2022 by DB) | |
| “social care” [title or abstract]  and  “UK” or “United Kingdom” or “England” | Restricted to ‘systematic reviews’ and years 2012 to 2022 |
| **b) Google** (search conducted in May 2022 by LD) | |
| Search terms | Comments |
| 1. “Social care" and "strengths and assets" | Searched following stakeholder feedback |
| 2. "Social care" and "three conversations model" and "prison" | Searched following stakeholder feedback |
| 3. "Social care" and "three conversations model" | To see if we could find anything useful from the wider community, i.e., not in prisons |
| 4. "Social care” and “adults in the community” and "England" and "examples of good practice" | To see if there were any examples of good practice of social care provision (or assessment) in England |
| **Trauma-informed approach searches** | |
| A basic google search was conducted on 25 October 2022 using the term ‘trauma-informed’ and ‘prison’ to identify recent articles and guidelines (date restricted from 01 January 2020 to 25 October 2022). This search was conducted following service-user feedback. | |
