# Supplementary material for: Developing an initial programme theory for a model of social care in prisons and on release (empowered together): A realist synthesis approach
Source: Med Sci Law. 2024 Jul 25;65(3):194–206. doi: 10.1177/00258024241264762 (PMC12149453; doi:10.1177/00258024241264762)
Supplement: sj-docx-4-msl-10.1177_00258024241264762 - Supplemental material for Developing an initial programme theory for a model of social care in prisons and on release (empowered together): A realist synthesis approach [file sj-docx-4-msl-10.1177_00258024241264762.docx]

**Supplementary Table S2: Criteria for initial screening (title/abstract)**

| **Inclusion criteria** | |
| --- | --- |
| Scope  Articles about identifying, screening, assessing and/or meeting social care (SC) needs of adults in or on release from prison.  Adult (18+) prisoners or ex-offenders.  Include if title/abstract refer to the term ‘social care’, or any of the 10 domains in the 2014 Care Act:  1. Managing/maintaining nutrition  2. Managing personal hygiene  3. Managing toilet needs  4. Being appropriately clothed  5. Being able to make use of home (in custody, prison) safely  6. Maintaining a habitable home environment (in custody, cell)  7. Developing/maintaining family/ personal relationships  8. Accessing/engaging in work, training, education or volunteering  9. Making use of necessary facilities/services in the local community (in custody, prison and any required community services)  10. Carrying out any caring responsibilities for a child | Population  Any adult prisoners/ex-offenders with SC needs, including specific sub-groups (e.g., prisoners with learning disabilities, older prisoners)  Include any studies about the perspectives of staff, peers, or family/friends of prisoners with SC needs |
| **Exclusion criteria** | |
| Exclude if there is no mention of SC or to any of the 2014 Care Act domains, e.g., if only refers to housing (which is not part of the Care Act) for ex-offenders generally.  Exclude studies that do not specify that participants are, or have been, in prison or in police custody e.g., studies of ‘forensic’ populations that do not specify a history of detention, or of people in other settings such as secure psychiatric facilities. Studies about prisoners of war should be excluded.  Exclude studies about young offenders. | |
