# Supplementary material for: Developing an initial programme theory for a model of social care in prisons and on release (empowered together): A realist synthesis approach
Source: Med Sci Law. 2024 Jul 25;65(3):194–206. doi: 10.1177/00258024241264762 (PMC12149453; doi:10.1177/00258024241264762)
Supplement: sj-docx-5-msl-10.1177_00258024241264762 - Supplemental material for Developing an initial programme theory for a model of social care in prisons and on release (empowered together): A realist synthesis approach [file sj-docx-5-msl-10.1177_00258024241264762.docx]

**Supplementary Table S3: Data extraction templates**

**a) Column headings from template data extraction form (prison literature) a) Column headings from template data extraction form (prison literature)**

Sheet 1:

| Study number | Author | Title | Journal | Year | Country | Indicate 'NR' here if you think this document is not relevant, and specify why | Type of document (e.g., empirical study, literature review, guidance, policy document etc.) | Does the document cover identification of SC needs? (y/n) | Does the document cover assessment of SC needs? (y/n) | Does the document cover provision for SC needs? (y/n) | Does the document cover SC needs on/ after release? (y/n) | Population (e.g., prisoners with learning difficulties, dementia, MH problems, older prisoners) | Reviewer initials |
| --- | --- | --- | --- | --- | --- | --- | --- | --- | --- | --- | --- | --- | --- |

Sheet 2:

| Study number | Type(s) of social care covered (e.g., employment, training, self-care, general) | Key relevant findings (quantitative) from emprical studies/systematic reviews (if a review, with meta analysis, include relevant results here) Type n/a if a discussion piece, guideline etc. | Key relevant findings (qualitative) from emprical studies/systematic reviews (if a review, include relevant excepts from narrative summary here) Type n/a if a discussion piece, guideline etc. | Relevant insights that may inform social care in prisons? Any useful recommendations? (these could be anywhere in the document but, for empirical studies/reviews, mostly likely in Discussion. We are interested in what works for whom and why, but also what may hinder successful identification, assessment and/or provision of SC needs) | Provide details of any examples of good practice which could inform social care in prisons | Realist approach: add your own ideas here for any 'if-then statements' or 'CMO' (context, mechanism, outcome) configurations | Add any other comments you have |
| --- | --- | --- | --- | --- | --- | --- | --- |

**b) Column headings from template data extraction form (community literature)**

| Source (author, year, title); study type; population; setting | Insights (findings; examples of good practice; recommendations; implications) | Reviewer’s ideas for any if-then statements | Reviewer initials |
| --- | --- | --- | --- |
