# Supplementary material for: Developing an initial programme theory for a model of social care in prisons and on release (empowered together): A realist synthesis approach
Source: Med Sci Law. 2024 Jul 25;65(3):194–206. doi: 10.1177/00258024241264762 (PMC12149453; doi:10.1177/00258024241264762)
Supplement: sj-docx-6-msl-10.1177_00258024241264762 - Supplemental material for Developing an initial programme theory for a model of social care in prisons and on release (empowered together): A realist synthesis approach [file sj-docx-6-msl-10.1177_00258024241264762.docx]

**Supplementary File S1: Full list of if-then statements, CMOs, consolidation, nuggets of information and sources/references**

| **Notes**:  a) ‘Specialised groups’ or subgroups referred to in some sources include people with LDDs, autism, mental health conditions, dementia, traumatic brain injury, history of or current trauma, and older adults in prison  b) This document includes the 16 specific CMOs. The final 4 consolidated CMOs are outlined in Table 2 of the submitted manuscript.  c) The complete list of references/sources used in developing and refining the IPT is provided from pages 91 onwards in this supplementary file. |
| --- |

**1. General – overarching issues which apply to all aspects of social care in prison/on release (i.e., identification; assessment; provision of care & support; release)**

| **Consolidated/refined if-then statements; CMOs** | **Original if-then statements / details / examples / ‘nuggets’ of information** |
| --- | --- |
| **1.1 Person-centredness** | |
| If staff could deliver person-led, person-centred and trauma-informed care which involved prisoners in developing care plans, using their strengths and assets, and provided accessible information, then prisoners would be more likely to have their social care needs met in a way that is tailored to them – Caiels et al 2021 (community); Care Quality Commission 2021 (community); Hagos et al 2021 (full); Hwang et al 2021 (full); Lennox et al 2021 (full); NICE 2019 (community); Sweeney 2021 (community); Levy et al 2018; NICE 2018a and 2018b (community); Flatt et al 2017 (full); Munday et al 2017 (full); National Institute for Health Research 2017 (community); Flynn et al 2016 (full); Slasberg & Beresford (2017); Coates 2016 (full); Henwood 2014 (community); Tinker et al 2014 (full); WHO 2014; Fox et al 2013 (full); Williams 2013 (full)  Person-centred CMO  C: Prisoners do not receive equivalent care to people living in the community, including person-centred care, due to prisons’ fragmented health and social care systems. The British Association of Social Workers describes person-centred care as “health and social care professionals work(ing) collaboratively with people who use services. Person-centred care supports people to develop the knowledge, skills and confidence they need to more effectively manage and make informed decisions about their own health and health care. It is coordinated and tailored to the needs of the individual. And, crucially, it ensures that people are always treated with dignity, compassion and respect.”  M - resource: An integrated health and social care system, in which staff are trained to deliver person-centred, trauma-informed care in collaboration with prisoners. The system enables prisoners to be actively involved in their assessments, individual goal setting, and personalised care-plans, with self-management being promoted where possible.  M response: Prisoners – dignity, empowerment, self-efficacy, motivation, engagement.  M response: Staff - empathy, compassion, respect.  O: Staff relate to prisoners in a dignified, compassionate, and respectful manner. Prisoners are more likely to have their health and social care needs met in a way that is tailored to them and they are more likely to engage with services. Improved working relationships. Greater equivalence of care. Enablement to live as independently as possible after release; more successful rehabilitation; improved community safety. | If staff delivered person-centred care (prisoners are involved in care plans, individualised care is given, and self-management is promoted) then older prisoners would benefit more – Hagos et al 2021 (full)  If focus groups with prisoners could be held, then any issues affecting their social care needs could be explored (such as the prison regime, physical environment, equipment needs, social isolation, purposeful activity) and acted upon in a person-centred way – Munday et al 2017 (full)    If prisons could adopt a whole-prison approach to identifying, supporting, and working with prisoners with Learning Difficulties and Disabilities (LDD), then their social care needs would be better met. In addition to appropriate training for staff, this could also entail ensuring that all prison information, forms, and digital systems are available and/or are designed in simple, plain English, with suitable adaptations to support those with dyslexia, and illustrative diagrams or images to accompany the text where possible – Coates 2016 (full)  “Policy, service planning and activity programmes should be developed according to global best-practice indicated by the UN Convention of the Rights of Person with Disabilities (2006) which recognises disability as evolving and shaped by interactions with the person in their environment” – Levy et al 2018  “Person centred approaches should be embedded in social care, ensuring service users are listened to and included in co-producing outcomes” – Levy et al 2018  “All adults in custody should expect care and support equivalent to that received by someone in the community. This principle of equivalence of care is the basis of the policy intent of the Act. This is crucial for ensuring that people in need of care and support achieve the outcomes that matter to them and that will support them to live as independently as possible after their detention. It will also contribute to the effectiveness of rehabilitation and improve community safety.” - NHS England and NHS Improvement 2021  If there could be a shift in thinking from “security” to “care”, and from “prisoner” to “citizen”, then this would help meet prisoners' social care needs - Hwang et al 2021 (full)  Maintaining client engagement:  If service users are shown respect and are properly informed via clear, open and honest communication from staff in the context of effective interagency collaboration, including shared practices and effective supervision for staff, then this could help to build rapport and trust with service users which in turn could encourage and maintain their engagement. This could include adapting communication style for individual clients – Lennox et al 2021 (full)  If older prisoners could be supported to live healthily in ways that are suited to them, then they would be less likely to heavily use healthcare services in later life (in prison or upon release) – Tinker et al 2014 (full)  Context: there are often overlapping social care needs between the community and prison  Mechanism: applying what has been learnt from community social care such as personalisation to a criminal justice environment  Outcome: Specific social care needs of individuals are more likely to be met in prison/upon release - Fox et al 2013 (full)  If careful consideration could be given to the best way to meet the needs of prisoners who require assistance with intimate personal care, such as showering and dressing, then their needs would be met in a dignified way – Tucker et al 2021 (full)  Reablement may offer a more cost and outcome effective solution than traditional care – one that may also be found more acceptable by those who would benefit from support. Reablement is a means of assisting individuals to lead full and independent lives by building their own skills to carry out tasks themselves, staying independent and safe. It is associated with better health-related quality of life and social care-related outcomes compared with conventional home care42.... may wish to work towards policies that encourage and support independence - and avoid providing unnecessary care that could lead to loss of independence, people losing the ability to take care of themselves or ‘deskilling’ – Scottish Prison Service 2017 (full)  If regular visits are arranged from healthcare assistants to provide support to prisoners with mobility issues, then particular social care needs such as bathing would be met - Di Lorito et al 2018 (scoping)  Example of good practice: “In the UK, HMP Wymott offers weekly visits from healthcare assistants, who provide support for bathing to prisoners with mobility issues; a programme of psychological interventions; self‐help books or referrals to chaplaincy in the occurrence of mental health crises; and the delivery of age‐friendly activities such as arts, yoga or cooking classes” - Di Lorito et al 2018 (scoping)  If a prison is unable to offer support services, then social care could be delegated to community-based organisations, thereby ensuring prisoners’ social care needs are met - Di Lorito et al 2018 (scoping)  Example of good practice: “In the Irish context, where prisons are not able to offer support services, social care has been delegated to community‐based organisations such as the Red Cross and the ageing prisoners have welcomed the initiative” - Di Lorito et al 2018 (scoping)  If interventions are given to older prisoners which tackle chronic health problems alongside co-occurring symptoms of PTSD, then those who experience PTSD alongside chronic health/pain/impairment in ADL can be better cared for, taking into account the complex interactions between their conditions – Flatt et al 2017 (full)  Almost half those assessed who are receiving assistance with ADLs are receiving it from their peers in prison. 15 of those assessed expressed reluctance to engage with formal care were it available. This suggests that while it is important to formalise responsibility and pathways for assessment and provision of care there should also be consideration of what type of support is available and how it is offered – Scottish Prison Service 2017 (full)  If prisons had older and disabled prisoner policies which provided age- and need-specific regimes, then many of the social care needs of these prisoners could be met - House of Commons Justice Committee 2013 (full) NOTE THIS WAS PRIOR TO THE CARE ACT  Asset-based vs needs-based goals: "Some System Change Practitioners said that the focus on the individual setting their own goals made a big difference to how they worked with people, and described adopting a more asset-based way of working" – Hough 2020 (full)  The World Health Organization (2014) advocates a ‘whole-prison approach’ including provision of:  • a healthy environment and a culture of care and rehabilitation;  • an atmosphere in which prisoners feel safe in the company of other prisoners and staff;  • opportunities for prisoners to talk to other people in confidence;  • opportunities for properly supervised care, including basic social care for prisoners by  other prisoners;  • opportunities, through visits, to maintain family links;  • information about the prison routine;  • ways to keep loneliness and boredom to a minimum;  • adequate food, opportunities for exercise and access to fresh air – WHO 2014  Insights from the community:  Supporting independence / person-centred care:  Use self-defined strengths, preferences, aspirations and needs as the basis on which to provide individualised care and support. Actively involve service users in all decisions. LAs must provide independent advocacy to enable people to participate in: needs assessment, care planning, the implementation process, and review, where they would otherwise have substantial difficulty in doing so - NICE 2018a (community, recommendations)  Focus on person-centred care, involving individuals in their care, training staff to be able to care for older adults appropriately based on their needs, ensure equal access to services for all individuals. Person-centred, tailored care and information: “tailored to their needs, strengths and preferences and is not determined solely by their age or learning disability… consider the communication needs of those with learning disabilities“– NICE 2018b (community, guideline - Older adults with learning disabilities)  “The person must be genuinely involved and influential throughout the planning process, and should be given every opportunity to take joint ownership of the development of the plan with the local authority if they wish, and the local authority agrees. There should be a default assumption that the person, with support if necessary, will play a strong pro-active role in planning if they choose to. Indeed, it should be made clear that the plan ‘belongs’ to the person it is intended for, with the local authority role being to ensure the production and sign-off of the plan to ensure that it is appropriate to meet the identified needs.” – Department for Health and Social Care 2018  The review (advancing care in care homes) emphasises a person-centred approach, individual needs, and personalised care - National Institute for Health Research 2017 (community, themed review)  Ensure personal care needs are responded to in a timely, appropriate, and dignified manner in line with the person's wishes and support plan – NICE 2018a (community, recommendations)  Co-production to enable people using care and support services to use their strengths and assets to shape services in their community - NICE 2019 (community, quick guide)  Interventions to promote physical activity among older adults:  “There are indications that purely cognitive strategies and BCTs might be less suitable for older adults than motivators more meaningful to them, including social and environmental support, and enjoyment coming from being physically active. A whole system-oriented approach is required that is tailored to meet the needs of older adults and aligned with social, individual and environmental factors." - Zubala et al 2017 (community, umbrella review)  Strength-based approaches to social care in prison may not only benefit prisoners, but also the prison/justice system as a whole (e.g., effective partnership working, economics) - Slasberg & Beresford 2017 (community)  If people with SC needs are seen as experts in their situation and given responsibility, and professionals work collaboratively with them, then they may be empowered. This could lead to greater self-efficacy and motivation, and better working relationships – Caiels et al 2021 (community, scoping review)  If a ‘skills around the person’ (SATP) approach is adopted, drawing on individual prisoner’s assets (skills, knowledge, experience, and attributes), then this could help meet the demands of the Care Act 2014 in terms of provision of care, and supporting prisoners to live their lives - Caiels et al 2021 (community, systematic review); Henwood 2014 (community, report)  “Leeds City Council document outlining the local use and benefits of taking a strengths-based approach to social care in Leeds. Indicates use of ‘3 conversations’ model as its mechanism for delivering strengths-based approach. Includes some vignettes from peoples’ experience of accessing services using a strengths-based approach.” - Caiels et al 2021 (community, systematic review)  The 3 Conversations Model in South Gloucestershire Council:  “This new approach to adult social care developed by Partners 4 Change aims to:  • Enable better experiences & outcomes for those contacting our service.  • Create a different way of working that enables practitioners to use their skills to develop more creative solutions & feel more satisfied in their roles.  • To better use scarce resources & to reduce waiting times” - Caiels et al 2021 (community, systematic review)  Advance care planning:  Humanization models of assistance and person-centred care can help avoid ‘tick box’ exercises - Poveda-Moral et al 2021 (community)  Trauma-informed approaches  “Trauma informed care is used in many good practice examples and takes a person-centred approach to someone’s history. A trauma informed approach to care aims to provide an environment where a person who has experienced trauma feels safe and can develop trust. … Trauma informed care creates a culture of thoughtfulness and communication, with staff continuously doing their best to learn about and adapt to the different and changing needs of the people they work” – Care Quality Commission 2021b  Supporting engagement with services:  “Promote engagement by providing services that:  are person-centred, empathetic, non-judgemental  aim to address health inequalities  are inclusive and pay attention to the diverse experiences of people using the service.  Consider using psychologically informed environments and trauma-informed care. Recognise that people's behaviour and engagement with services is influenced by their traumatic experiences, socioeconomic circumstances and previous experiences of services.” – NICE 2022  “In trauma-informed approaches, there is an understanding of the complex and pervasive impact of trauma on a person’s worldview, relationships and ways of engaging with services and staff” - Sweeney 2021, community, guidelines) |
| **1.2 Trauma-informed care in the CJS** | |
| Trauma-informed CMO  C: People who have suffered trauma are over-represented in prison settings and prisons also house the most severely traumatised people in society  M – resource: Education and training for prison staff which incorporates trauma-informed knowledge and practice.  M – response: Prisoners: Feelings of safety and trust  O: Less risk of re-traumatisation; prisoners less anxious about acknowledging their needs; improved relationships; greater engagement with services; more self-compassion; improved self-care | If prisons could develop trauma-informed interventions for offenders, then their health and social care needs will be better met and both length of stay and rates of recidivism will reduce - Flynn et al, 2016 (full)  “recognising trauma was important to support recovery and avoid re-traumatisation” - McAnallen & McGinnis 2021  “where practitioners were committed to trauma-informed practice, they were important mediators for its integration into organisational practices” - McAnallen & McGinnis 2021  “Becoming trauma-informed is always a journey and experts describe a range of stages which can be summarised as:  • Trauma informed: ‘what we know’ – the knowledge about adversity and  trauma and its effects on individuals, communities, and society  • Trauma responsive: ‘what we do’ – creation of an environment for healing and recovery  • Trauma specific: ‘what services we provide’ – providing actual therapeutic  approaches that focus on trauma to help healing and recovery.  A criminal justice system provides appropriate service when it incorporates all three levels” – Durr 2020  “It is submitted that the focus of penal policy and practice should be recalibrated to put healing at the centre of relationships and interventions, assisting ‘unrecovered trauma survivors’28 with offending behaviour to make better sense of themselves and their multiplicity of personal struggles. This would enhance people’s self-compassion and relational abilities, equipping them to focus on their strengths and acquire skills, such as an education, to pursue their vision of a good life” - Mulcahy 2019  “A judicial institution is full of elements that may evoke memories of traumatic events that have been experienced. These include physical examinations, the restriction of privacy, reduced freedom of movement. The conscious handling of such elements by the prison ideally reduces the stress that these elements can cause in traumatized inmates” - Krammer et al 2019  “A greater provision of individually delivered trauma processing therapies in prison is recommended in line with trauma-informed care initiatives. We found an over-reliance on phase 1 stabilization interventions delivered by unqualified staff, however where trauma processing interventions are not feasible due to practical or safety concerns, stabilization interventions can still be helpful in reducing PTSD symptoms. A stronger partnership between prison operational system and prison health care providers is essential for successful implementation of trauma-focused interventions. Prisons may wish to consider medical holds for prisoners completing trauma-focused treatments, to reduce therapeutic barriers (such as transfers or scheduling conflicts) which may increase the risk of destabilization” – Malik et al 2019  “Being trauma-informed means recognising the impact that trauma … has on an individual and in acknowledging this, providing appropriate support to that person. A trauma-informed approach is a change of perspective from “What’s wrong with you?” to “What happened to you?” … seeks not to  re-traumatise with blame and sanction, but to recognise strengths and skills, build confidence and re-educate – embedding new coping skills to enable recognition and regulation of behaviour (Substance Abuse and Mental Health Services Administration, 2014b).” – McCartan 2020  Developing a ‘person first, trauma-informed workforce’:  Place the individual service-user at the centre of the process, allowing their voice to be heard and enabling them to move forward at a sustainable pace; promoting desistance, behaviour change, harm reduction and prevention.  “Workplaces need to have trauma-informed practice embedded at a policy level, ensuring it is a key plank in all organisational policies and factored into the development of new policies.”  “Being trauma-informed should be part of the day-to-day practice in an organisation; it should be constantly considered and developed. It should be written into all aspects of the organisation’s activities and be reflected in development, planning and maintenance of all working practices. It should be the subject of clear leadership in all parts of the organisation.”  “… develop a space for service users that is not trauma inducing or triggering, and where they feel able to engage with treatment, rehabilitation or supervision without feeling that they are at risk of relapse. This is a challenge in criminal justice settings, but one that needs to be considered as the shape, layout and flow of a building may have a traumatic impact on service users in general; especially if their traumatic experiences were criminal justice related.”  “… training staff in how best to communicate and interact with service-users. This involves staff training, appropriate leadership and awareness raising. Being trauma-informed needs to be at the forefront of practice in all forms of communication, support, and interactions, especially with challenging and difficult service users” - McCartan 2020  Insights re: trauma-informed care from HM Inspectorate of Probation (Petrillo & Bradley 2022):  Report refers to ‘Six key principles’ as espoused by Fallot and Harris, 2006; Harris and Fallot, 2009; Keeping Bristol Safe Partnership, 2021:  **Safety**: •Throughout the organisation, staff, and the people accessing services should feel physically, emotionally, and psychologically safe. The physical setting and interpersonal interactions within service spaces should promote a sense of safety.  **Trustworthiness**: •Organisational processes and decisions are conducted transparently, with the goal of building and maintaining trust among staff and people who use the service.  **Collaboration**: •There is true partnering and levelling of power differences between practitioners and people using the service, and also among staff at all levels of the organisation, from Administrators to Directors. There is recognition that healing happens in relationships and in the meaningful sharing of power and decision-making.  **Empowerment**: •Throughout the organisation, individuals’ strengths and skills are recognised, built on, and validated, and new skills developed as necessary  **Choice**: •The organisation aims to strengthen the staff and people on probation’s experience of choice and recognise that every person’s experience is unique and requires an individualised approach.  **Inclusivity**: Organisations intentionally recognise and address inequalities, oppression, and marginalisation. Practice is characterised by sensitivity and humility in responding to diverse needs. Organisations understand the impact of collective and historical trauma.  Example of an initiative at one UK prison – the ‘calm and compassion’ course:  “… a six-week course, comprising of weekly 2.5 hour group sessions and in-cell work. During the group, participants are supported to turn towards feelings of warmth and kindness through psychoeducation, group activities and techniques that aim to build awareness and strengthen their capacity to attend, think and respond in ways that are more compassionate to both themselves and others. Some specific activities within the group include discussions on our development according to relational contexts, mindfulness and compassionate imagery exercises, learning to restructure responses to our self-critical thoughts and improving self-care and other compassionate behaviours” – Wright 2021  “Trauma frequently diminishes people's sense of self-worth and self-belief, and breaks their faith in  others and in authority. Assessments are the present-day focal point for the desperation and accumulated trauma of a lifetime. Yet assessments can compound trauma where people feel that another person has the power to decide whether or not help is received … Common feelings associated with undergoing assessment include worry, desperation, shame and fear of judgment alongside a fragile sense of hope. Trauma survivors may question their right to support, feeling that others have greater needs. Many feel that they need to prove they are worthy of support yet feel profoundly unworthy. There is potential for significant harm where a trauma survivor reaches out for help but is turned away, reinforcing shame, worthlessness and hopelessness. Survivors are aware of the potential for rejection which causes fear and anxiety, particularly where they have no alternatives” – Sweeney 2021  Study protocol for an RCT of a trauma-informed re-entry service for young males (18-35):  “It cannot be assumed that evidence-based trauma treatment approaches for the general population are appropriate or effective approaches to responding to lifetime traumatic experiences among males leaving correctional facilities.” The intervention will use “trauma-trained re-entry specialists to deliver the intervention and provide re-entry supports to help improve community stabilization. Trauma-trained re-entry specialists will be masters-level social workers or social work graduate students who have completed a comprehensive two-week training. In addition to treatment specific instruction, this training focuses on the effects of trauma, barriers to re-entry, cultural competency, and skills for creating safety during the sessions”– Pettus et al 2022 |
| **1.3 Staff levels, training, and learning** | |
| If prisons could ensure sufficient staffing levels and if prison and probation staff received appropriate, trauma-informed, co-designed education and training, then prisoners' social care needs could be better identified and met in prison and on release – Forsyth et al 2022 (full); Scottish Government 2022 (full); Bradley 2021; CLINKS & RECOOP 2021 (full); Hagos et al 2022 (full); Hagos et al 2021 (full); Hughes & ten Bensel 2021 (full); Age UK 2019; National Probation Service 2019 (SCoR); Ogletree et al 2019 (community); Clinks 2018; Tucker et al 2018 (full); HMIPS 2017 (full); Hayes et al 2012 (full)  If special consideration is given to underserved and disadvantaged groups in prison, including those with disabilities, dementia, and older groups and including the use of specialised screening tools and staff training, then the needs of these groups can be better identified and met - Brooke & Rybacka 2020 (full); Brooke et al 2020 (full); Garcia-Martinez & Alvarez 2021 (full); Favril et al 2020 (scoping); Forsyth et al 2020 (scoping); Du Toit et al 2019 (full); Levy et al 2018; NICE 2018 (community); Skarupski et al 2018; Coates 2016 (full); van Dooren et al 2016 (full); Young et al 2016 (full); Anderson 2015 (full)  If social workers and other social care practitioners are provided with additional, appropriate training on working with and assessing the social care needs of adults in prison, then they will be better equipped and more confident in their role - Tucker et al 2018 (full)  Staff training CMO  C: All prison officers in the UK receive training and must complete an apprenticeship. There are opportunities to specialise in working with disadvantaged or vulnerable people, but many officers are not trained or educated to recognise, understand, and address individuals’ social care needs. Social workers receive some training on social care in the CJS but some do not feel confident or prepared enough to work with and assess this client group.  M - resource: A specific education and training programme for prison and probation staff and social care practitioners covering the social care needs of people in prison and on release. The programme is codesigned with prisoners and specialist voluntary organisations, and mandatory for all prison staff and probation workers. It includes education and training on trauma-informed knowledge and practice. It also covers the importance of good communication between staff and prisoners, and of information sharing.  M - response (care receivers): feel better understood and valued; build trust  M – response (staff): feel empathy and respect; gain confidence in role.  O: Prison staff are more responsive to prisoners’ social care needs and confident to make decisions and referrals when appropriate; social care practitioners are better equipped to work with clients in the prison setting; prisoners’ social care needs are more readily identified, understood, and addressed. | Training for prison/probation staff  If training on common health and social problems among older prisoners, including on dementia and frailty, is provided officers then this would help them to refer prisoners to the appropriate service provider – Hagos et al 2022 (full)  Training programmes should be co-designed with prisoners and supported by specialist voluntary organisations – CLINKS & RECOOP 2021 (full)  If prison staff are given special training, then they will be able to recognise that prisoners with social care needs cannot necessarily do the same tasks or at the same pace as other prisoners - Hayes et al 2012 (full)  If training is provided to address gaps in knowledge and management of older prisoner care, and to improve communication and collaboration, then custodial staff will be better equipped to deal with older prisoner care - Hagos et al 2021 (full)  Training could be delivered in short, hands-on sessions with interactive designs – Hagos et al 2021 (full)  and/or in the form of workshops which are presented to prison staff, prisoners, and health and social care professionals at the same time to facilitate consistent understanding of issues and initiate communication between groups - Brooke & Rybacka 2020 (full)  Training could be included in Prison Officer Entry Level Training (POELT) - CLINKS & RECOOP 2021 (full)  Train prison staff how to care for older prisoners - Age UK 2019  Where health and prison care staff take the lead in identifying prisoners with social care needs, it is important to determine what training these staff need; health care problems not identified on admission are often left unidentified - Tucker et al 2018 (full)  The importance of disability training for prison guards was asserted to improve interactions with prisoners with intellectual disability. Key informants noted that prison guards, and most corrective service staff, don’t seem to have a lot of education working with anyone with a disability - van Dooren et al 2016 (full)  If there is mandatory training/education aimed at enabling prison staff to understand and support the older population and others with social care needs, and if this is co-designed with prisoners supported by specialist voluntary organisations, then the social care needs of such prisoners would be better identified and met - CLINKS & RECOOP 2021 (full)  If prisons could increase staffing levels including an officer key worker for each prisoner, then prisoners' social care needs would be better identified - Forsyth et al 2022 (full)  If training is provided to judicial and penal staff on prisoners with intellectual disabilities and their rights, then adequate adaptations can be made for these people so that they can understand how the prison functions, their rights and obligations, and the function of staff - Garcia-Martinez and Alvarez 2021 (full)  Increased staffing levels and provision of an officer ‘key worker’ for every prisoner – Forsyth et al 2021 (full)  Targeted, disability-specific training for prison officers to improve their interactions with prisoners with intellectual disability was seen as important – Young et al 2016 (full)  Importance of adequate staffing levels and of acknowledging that not all staff are equally suited to working with older prisoners. “This role demands some particular qualities, experience and skills. Suitable officers should be selected for the care of older prisoners and provided with appropriate  Training” - Her Majesty’s Inspectorate of Prisons in Scotland (HMIPS) 2017 (full)  Summary of key recommendation regarding staff: Action to address staff shortage and retention; mandatory induction materials for all staff on health inequalities and services available – Scottish Government 2022 (full)  “The SPS should ensure that staff who work with older prisoners are identified as suitable for the role and appropriately trained. They should be provided with the training necessary to support them in their tasks, including specific training in dementia care and other age related health subjects. Their work should be valued and supported in prisons, with sufficient resources committed to provide the necessary care for these prisoners.” - Her Majesty’s Inspectorate of Prisons in Scotland (HMIPS) 2017 (full)  “Identify ways for sharing best practice and knowledge across all domains of practice, including through research with academic partners and other external organisations to evaluate and inform future work and to support the development of a culture of learning and inquiry” – Levy et al 2018  “Develop opportunities for joint learning for health, social care and SPS staff on understanding social care, disability, working with diversity and the impact of prison on health and wellbeing” – Levy et al 2018  If prison officers were given more training, then they will be less likely to miss social care needs related to mobility and relationship problems – Forsyth et al 2018 (full)  “Prison officers, voluntary sector staff and volunteers should be provided with training to equip them to identify and support people with health and care needs, which should include … trauma-informed approaches” – Clinks 2018  Traumatic events “shape how we experience and construct our view of the world, our feelings of safety. These experiences influence how we trust^13^ individuals and the subsequent relationships that we build” – Bradley 2021  “… the delivery of the BTI [‘Becoming Trauma Informed’] programme across the long-term **high secure** Male prison estate has been in progress since May 2018^32^” but…. “BTI and TIP is yet to be embedded within local and lower category male prisons.” – Bradley 2021  “trauma should be a strategic priority for HMPS and the Ministry of Justice, who have the strategic oversight of institutional transformation to support the implementation of TIP” – Bradley 2021  “Staff at every level of the institution, regardless of role, should receive training and information about trauma. Specialised training can be tailored and provided depending on the needs of the staff. … Training should also focus on supporting, prioritising and emphasising staff wellbeing and development” – Bradley 2021  The author notes that leadership days, workshops, training, Toolkits, and follow-ups delivered by charities such as ‘One Small Thing’ are structures to guide and support prisons to develop TIP.  The ‘Working with Trauma Quality Mark’ has been developed in the UK in partnership with the charity One Small Thing – Bradley 2021  The NPS (National Probation Service) will: Develop a learning package for NPS staff to improve their knowledge of social care legislation, identification of social care needs, types of support available and referral pathways - National Probation Service 2019 (SCoR)  Parole officers stated there was a lack of training or resources that primarily addressed how to manage older parolees. Over half (n = 11) expressed the need for age-specific training in areas of addressing health care and housing. Many older parolees use canes, walkers, and wheelchairs and have various mental and physical health issues that become problematic for parole officers who are responsible for supervising them - Hughes & ten Bensel 2021  Specific populations:  ‘Vulnerable’ or disadvantaged groups include those with dementia, learning disabilities, autism spectrum conditions, trauma, traumatic brain injury, ADL disabilities, and some older prisoners  Prison staff could be educated to identify and work with those with dementia or mild cognitive impairment - Brooke et al 2020a (full)  If all correctional staff (nurses, guards etc) are trained in how to care for older prisoners with dementia, then better care can be provided - Du Toit et al 2019 (full)  If prison staff are educated in how to identify and work with those with dementia in prison, then social care outcomes will improve – Brooke & Rybacka 2020 (full)  If dementia education workshops could be delivered to prison staff, prisoners and health and social care professionals together, then this would facilitate consistent understanding of issues and initiate communication between these groups - Brooke & Rybacka 2020 (full)  If all prison staff were trained to recognise dementia and mild cognitive impairment in prisoners, then identification of social care needs would be more likely to occur - Forsyth et al 2020 (scoping)  Prisons could use a consistent and rigorous assessment mechanism to set a baseline against which to measure individuals’ academic performance and screen for learning difficulties and/or disabilities – Coates 2016 (full)  If more appropriate screening tools were developed to identify cognitive impairment and social care needs in older prisoners, then their care needs could be better targeted - Brooke et al 2020 (full)  If there was a focus on identifying the social care needs of specific populations such as those with learning disabilities or autism spectrum conditions, then the prison would be better prepared to then meet the needs of these populations - Anderson 2015 (full)  If there is comprehensive screening for social risk factors associated with self-harm (such as unemployment, homelessness) upon entry into prison, then social care provision can be facilitated that will reduce the risk of self-harm among prisoners - Favril et al 2020 (scoping)  Training for specialist social care staff  While a survey conducted after the 2014 Care Act found that it was chiefly specialist social care staff who were assessing prisoners in most LAs, there is nevertheless concern that assessing this group may require additional training and that it is unclear how well-equipped practitioners were to work with them - Tucker et al 2018 (full)  Community insights  Co-production of information, protocols, organisational policies and procedures, and staff training – NICE 2018 (community, recommendations)  Ensure practitioners have time to build relationships and rapport with prisoners; training and supporting of practitioners to work in this way - NICE 2018 (community, recommendations)  Suggestions re: how to avoid omissions in care include better communication and education for staff - Ogletree et al 2019 (community – nursing homes) |
| **1.4 Multidisciplinary teams (MDTs) and collaborative, joint working; integrated care systems** | |
| If prisons adopted an integrated health and social care system, with MDTs and social care leads/coordinators and fostered collaborative, inter-agency working and co-design including with voluntary organisations, then staff could work together proactively to facilitate timely and appropriate screening, assessment, person-centred care and release planning, and delivery of care along with preventative measures – Forsyth et al 2022 (full); Stakeholder Workshop (Feb 2022); Kenkmann et al 2022 (scoping); Scottish Government 2022 (full); Care Quality Commission 2021 (community); Forsyth et al 2021 (full); Lennox et al 2021 (full); NHS England and NHS Improvement 2021; Her Majesty’s Inspectorate of Probation; Forsyth et al 2020 (scoping); Frost et al 2020 (community); Kelly et al 2020 (community); Rowe et al 2020 (full); Age UK 2019; Briggs et al 2018 (community); Chadborn et al 2019 (community); Peacock et al 2019 (full); Care Quality Commission & HM Inspectorate of Prisons 2018 (full); Care Quality Commission 2018 & 2016 (community); Di Lorito et al 2018 (scoping); Forsyth et al 2018 (full); Hollomotz et al 2018 (full); Levy et al 2018; NICE 2018 (community); Tucker et al 2018 (full); Goodman et al 2017 (community); Kirst et al 2017; NICE 2017; Shaw et al 2017; Anderson 2015/16 (full); Mackie & Darvill 2016 (community); NICE 2016; NOMS) 2016; Pearmain 2016 (full); Prisons and Probation Ombudsman 2016 (full); Young et al 2016 (full); Winters et al 2016 (community); Forsyth et al 2015 (full); O’Hara et al 2015 (full); McKenna et al 2014; Trivedi et al 2013 (community); Moll 2013 (full); Raghavan 2013 (full); O’Hara 2012 (full)  Integrated working CMO  C: Prison, healthcare, and social care staff often function disparately and have conflicting priorities and goals, resulting in inefficiencies and gaps in the identification, assessment, and provision of prisoners’ health and social care needs.  M - resources: Integrated health and social care, with proactive key workers, social care leads, and multidisciplinary care teams (MDTs) characterised by co-design, collaboration, shared goals and values, memorandums of understanding (MOUs), shared IT systems, formal agreements on information sharing and respective roles and responsibilities, promotion of work interdependence, and shared training and supervision. Includes out-of-hours access to social care support, including overnight. Aims for regular staff rather than temporary agency workers. MDTs also collaborate with voluntary and community organisations including those with expertise in equality and diversity.  M - response (staff): Motivation (to learn from and support each other); mutual respect.  (Prisoners): feel valued and included  O: Increased likelihood that prisoners’ health, social and custodial care needs are identified, assessed, and met adequately and in a timely, person-centred manner including in an emergency. Consistency of care. Duplication avoided. Increased job satisfaction for staff while retaining their sense of professional identity; a legitimising function which enables staff to focus on ‘working with’ rather than ‘doing to’ prisons. | Summary of key recommendation regarding information: Join up health and care data in prisons; information sharing protocols between organisations – Scottish Government 2022 (full)  Importance of having someone to formally coordinate care provision, including through the gate. Issues around Information sharing for care plans; continuity of care. Issue of ‘silos’ needs to be addressed (in prisons AND on release) – stakeholder workshop, February 2022  Multidisciplinary team: “A group of professionals from different disciplines who each provide specific support to a person, working as a team. In prison settings, a multidisciplinary team may include physical and mental health professionals, prison staff, National Probation Service and/or community rehabilitation company (CRC) representatives, chaplains and staff from other agencies, such as immigration services and social care staff” – NICE 2016  The multi-disciplinary team, its role and composition should be key to the delivery of integrated health and social care in prisons – Levy et al 2018  We recommend that social work play a lead role in co-ordinating and assessing social care in prisons and that teams have a diversity of professionals involved, including medical or nursing staff, occupational therapists, mental health staff, rehabilitation workers and others – Levy et al 2018  If prisons had permanently based local authority social workers, then more timely assessments could be made - Care Quality Commission & HM Inspectorate of Prisons 2018 (full)  If prisons contained multidisciplinary care teams, then the health and social care needs of older prisoners would be better met – Peacock et al 2019 (full)  MDT could include social workers, occupational therapists, mental health and health care staff - Care Quality Commission & HM Inspectorate of Prisons 2018 (full)  (Prisoners with mental health problems)  If there was clearer role definition within multi-disciplinary teams and more effective collaboration with between community health and correctional staff, then released offenders will be more likely to engage with social care and other services post-release – McKenna et al 2014 (full)  If social care interventions could be considered alongside healthcare interventions, then prisoners with social care needs would be better equipped with skills to reintegrate into society successfully - Kenkmann et al 2022 (scoping)  If prisons could draw upon the expertise of voluntary organisations, then prisoners with social care needs would be less frail on release, more successfully reintegrated, and less likely to require care home placements - Kenkmann et al 2022 (scoping)  If prisons had full-time social workers based internally, then prisoners’ assessments would be timelier and the opportunity for joint working and good working relationships between prison, health, and social care staff would be enhanced - Forsyth et al 2020 (scoping)  “Having a lead care coordinator in place for people in prison who are receiving care from different teams means that they can receive joined-up care. The lead care coordinator can ensure good communication within the multidisciplinary team, which can include health, social care and custodial teams. By working with the multidisciplinary team the lead care coordinator can help to ensure that people in prison receive help and support to manage their health and social care needs. In addition, people in prison can receive help to reduce avoidable exacerbations of their physical and mental health conditions, reducing the risk of unplanned hospital admissions.” – NICE 2017  (Related to severely mentally ill prisoners on release) “Integrating health and social care services to meet prisoners’ needs in a holistic way on release is … vital to successful community reintegration. Thus, effective release planning and resettlement requires not only continuity of health care but also measures designed to meet the economic and social needs of the prisoner” – Shaw et al 2017  (Collaboration around release of prisoners with MH problems) If staff could be motivated and supported by their own service to work within and across service boundaries, including sharing of information, shared care pathways, joint planning and clear accountability, then this would help enable inter-professional practice within and between MDTs in prisons and externally. This could be facilitated by agreeing clear roles and responsibilities for interagency professionals and by regular forums – Lennox et al 2021 (full)  (Prisoners with IDs) Key informants emphasised the importance of effective working relationships. Early planning and through-care would ideally involve the collaboration of multiple stakeholders. Information exchange between prisons and supporting agencies was seen as critical to  providing effective support in the community – Young et al 2016 (full)  If support is provided for both prison service and healthcare staff and if there is clear guidance in terms of respective roles, then a better understanding of how each other’s organisation works will be achieved, thereby improving the effectiveness of care pathway development and intervention implementation – Forsyth et al 2020 (full)  Tensions between services and across systems (eg in mental health, disability and justice) as to who was responsible for what (including who was responsible for funding what). Resentment between disability service providers related to perceived funding inequities – Young et al 2016 (prisoners with IDs, full)  For former prisoners with a history of mental disorder: If there were improved and integrated support for those with MH issues being released from prison, then they would be more likely to successfully access the services they require - Cutcher et al 2014  “Each service within a prison has its respective responsibilities and duties concerning people with a learning disability and autistic people. Healthcare services need to be aware of the ways in which other agencies support people, to provide a more coordinated, streamlined approach with time and resources focused appropriately, and duplication or unnecessary overlap avoided. This approach requires robust information sharing, joined-up working and pathways of support/referral between all agencies supporting the same people.” - NHS England and NHS Improvement 2021  “The ageing of the prison population will bring with it both increasing demand and increasing complexity in prisoners’ health and social care needs. People may increasingly have care and support needs on their entry into the criminal justice system, which must continue to be met within the prison setting. New needs may increasingly emerge during the time people spend in prison. Continuity of care and support will need to be a key element in planning for and supporting prisoners’ returns to the community. Rising to these challenges will require adequate resourcing and close collaborative working between health, social care and justice partners.” – Welsh Parliament Health, Social Care and Sport Committee 2021  “People in prison should be entitled to the same quality of integrated health and social care that is available in the community. This should be more clearly articulated in policy and legislation. … Integrated working requires understanding and collaboration between professions and service users” – Levy et al 2018  Ensure local authorities and HM Prisons and Probation Services work together to ensure prisoners’ social care needs are met; provide more resources to help the third sector improve older prisoners’ lives – Age UK 2019  If prisons networked with charities and other external organisations focused on social care, then the SC needs of prisoners and ex-prisoners are more likely to be identified and met - Di Lorito et al 2018 (scoping)  If prison staff had access to a 24-hour telephone line with the local authority and provider, then this would facilitate rapid assessments and emergency care packages as needed - Care Quality Commission & HM Inspectorate of Prisons 2018 (full)  If each prison had an identified and proactive social care lead, alongside comprehensive local agreements between prisons and social services, then this would help ensure that local social services effectively coordinate care for all prisoners, regardless of their geographical allegiances - O’Hara et al 2015 (full)  If each prison had a local lead for adult social care, then coordination between prisons and local authorities could occur to create care plans for each eligible prisoner and ensure no prisoner's needs go unnoticed - Prisons and Probation Ombudsman 2016 (full)  “Each prison must nominate a local lead for Adult Social Care who will have responsibility for liaising with local authorities, their providers and provider staff” - National Offender Management Service (NOMS) 2016  If prisons had Older Prisoner Leads or Disability Liaison Officers who developed close relationships with older prisoners and others with social care needs, and had the relevant resources, then they could more effectively provide care and support - House of Commons Justice Committee 2013 (full) NOTE THIS WAS PRIOR TO THE CARE ACT  Recommendations from the Prison Reform Trust re: supporting vulnerable offenders:  Encourage collaborative working at the strategic level with a range of partners, such as Police and Crime Commissioners, Probation Trusts and the NHS National Commissioning Board; use aligned or pooled budgets, for example Community Budgets, to achieve better value for money from different streams of funding for people with multiple needs; offer social care expertise to other local services – Prison Reform Trust 2013 NOTE THIS WAS PRIOR TO THE CARE ACT  If prisons had clear pathways to oversee referral and assessment procedures, with a multidisciplinary team (e.g., social workers, occupational therapists, mental health and health care staff) then all social care needs would be more likely to be assessed and addressed - Care Quality Commission & HM Inspectorate of Prisons 2018 (full)  If prisons had designated social care leads (could be prison employees, representatives from the local authority or staff employed by the social care provider), then prisoners' needs would be better identified, assessed and addressed - Care Quality Commission & HM Inspectorate of Prisons 2018 (full)  If prisons shared information and best practice, then strategies that work could be replicated, allowing more people with dementia to receive appropriate care in prison - Prisons and Probation Ombudsman 2016 (full)  If OMIC key workers collaborated effectively with social care workers, then prisoners’ social care needs would be better met - Her Majesty’s Inspectorate of Probation  If OMIC key workers successfully engaged with prisoners, then unmet social care needs may be avoided - Her Majesty’s Inspectorate of Probation  If OMIC key workers are trained and appropriately supported, and successfully integrated into social care models, then prisoners’ will be better supported - Her Majesty’s Inspectorate of Probation  Observation – OMIC’s focus appears to be on reducing reoffending – however it focusses on many social care issues – housing, benefits etc. – Very relevant for preventing social care problems.  Consider a professional older prisoner lead who would receive specialist training and be given adequate dedicated time - O’Hara et al 2015 (full)  An MDT working effectively together would facilitate the introduction of preventative measures - Di Lorito et al 2018 (scoping)  Some LAs identified MDT meetings and reviews as a fruitful means of eliciting potential referrals - Tucker et al 2018 (full)  If multidisciplinary teams developed a mutual respect, shared knowledge and supported each other, then programmes would be delivered more successfully - Rowe et al 2020 (full)  If there was collaborative working, shared goals and values between disciplines in the prison (e.g., prison officers and healthcare staff), and engagement at all levels, then social care interventions would be more successful - Forsyth et al 2022 (full)  If multidisciplinary teams were implemented in prison, and members understood each other’s roles and were regularly trained and supervised, then more cohesive care could be provided to older adults in prison - Hagos et al 2021 (full)  If prisoners received support from regular staff rather than agency or temporary staff, then staff would become more familiar with individual prisoners’ needs and more likely to build a rapport, which would better facilitate the care and support provided - Care Quality Commission & HM Inspectorate of Prisons 2018 (full)  “Trans-disciplinary teams can include social workers, social care practitioners, youth workers, gardaí, prison officers, probation officers, GPs, psychiatrists, psychologists, etc. Trans-disciplinary teamwork is where: Members share roles systematically across discipline boundaries. The primary purpose is to pool and integrate the expertise of team members to provide more efficient and comprehensive assessment and intervention services; the communication style involves continuous give-and-take between all members on a regular, planned basis; professionals from different disciplines teach, learn, share and work together to accomplish a common set of intervention goals; the role differentiation between disciplines is defined by the needs of the situation rather than by discipline-specific characteristics; assessment, intervention, and evaluation are carried out jointly by members of the team” – O’Hara 2012 (full)  If services (forensic, healthcare, social care, families) work together and share information, then the needs of those with autism and/or learning difficulties who have sexually offended can be met effectively, and risk of reoffending can be reduced - Hollomotz et al 2018 (full)  If prisons followed a social care model that is present in the community based on collaborative working between health and social care professionals, then they could be better prepared to meet the needs of different populations - Anderson 2015 (full)  If third sector organisations (such as older adult specialist services) could help identify and appropriately address other prisoners’ social care needs, then needs will be better met – Forsyth et al 2021 (full)  Examples of good practice: i) The Older Prisoners Action Group (OPAG) worked at the Isle of Wight prisons, bringing together professionals from the Department of Health, prison and probation services, the NHS, Birmingham University and a number of charities to offer a continuum of care for ageing prisoners. A key objective of the alliance was to advocate for the adoption of a multi-stage common assessment procedure for older inmates, which would create a single appraisal process integrating health and social care needs bridging the transition from prison to resettlement. Ii) Age UK play an integral role in the release and resettlement of older prisoners at Norwich, providing an advice and support service on finance and accommodation and following up these issues after release. iii) A day centre for older men at Stafford is used as a platform for Age UK to run a Senior Citizen Group to provide individually tailored advice on benefits, resettlement support. Iv) Both California Men’s Colony and Laurel Highlands had set up a system for medical and psychology staff to provide continuity of care information to receiving personal care facilities, mental health or Department of Public Welfare (DPW) sites. Laurel Highlands employ a Social Worker responsible for coordinating scheduling and destination details - Moll 2013 (full)  (Prisoners with MH problems): Authors note that advanced communication and information sharing is the foundation of transitional care programmes – Pearsall et al 2014 (SCoR)  *Inter-agency collaboration:*  If there was collaborative working with formal agreements regarding information sharing and respective roles and responsibilities between prison, LA and other external practitioners, then social care interventions would be more successful - Forsyth et al 2018 (full)  If prisons and local authorities developed MOUs (memorandums of understanding) and kept them up-to date in line with the 2014 Care Act, and formed joint working arrangements, then this could ensure that the social care needs of prisoners are met - Care Quality Commission & HM Inspectorate of Prisons 2018 (full)  If there was multi-agency working between prisons and external agencies, then social care needs would be better met - Forsyth et al 2015 (full)  Coordinated approach between health care and social care services for older prisoners - O’Hara et al 2015 (full)  Contact with local social care services for older prisoners - O’Hara et al 2015 (full)  Written protocol between health care and social care services for older prisoners - O’Hara et al 2015 (full)    Sufficient communication from social services for older prisoners - O’Hara et al 2015 (full)  Contact with specialist older adult organisations - O’Hara et al 2015 (full)  If social care workers work well with prison staff, then they will better understand the restrictions of the prison environment, and be better equipped to assess the social care needs of an individual in prison - Pearmain 2016 (full)  Local authorities could promote cooperation between NHS, prison staff, health commissioners and the prison and probation service, which would help to develop good practice - Di Lorito et al 2018 (scoping)  If the role of social work practitioners in prisons was incorporated into community teams’ wider casework, then there would be more opportunity for additional staff to develop experience with this client group, and the promotion of equitable care and potential for continuity upon release (if within the same authority) would improve - Tucker et al 2018 (full)  *Rival statement:*  If the role of social work practitioners in prisons is undertaken by specialist practitioners, then there would be greater potential for close working relationships with prison staff and practitioners would develop more knowledge of the prison and legal system - Tucker et al 2018 (full)  If social care is provided by prison healthcare staff, and resources become stretched, then they may prioritise their healthcare role over the provision of social care, resulting in unmet social care needs - Tucker et al 2018 (full)  *Rival statement:*  If social care is provided by external domiciliary care providers, then there may be problems relating to limited access to prisons in the evenings and at night, and time taken to obtain security vetting - Tucker et al 2018 (full)  All staff should place emphasis on the care and support that is needed rather than punishment, which should make them more willing to respond effectively to prisoners’ social care needs - Di Lorito et al 2018 (scoping)  If NOMS produced guidance for prisons to liaise with LA social care teams, and issued a Prison Service Instruction specifying the extent to which it expects officers to carry out basic social care, then there would be less confusion about roles and responsibilities - House of Commons Justice Committee 2013 (full) NOTE THIS WAS PRIOR TO THE CARE ACT BUT CONFUSION REMAINS REGARDING ROLES AND RESPONSIBILITIES  It is not clear who is responsible for the provision of social care in prisons in Scotland – Scottish Prison Service 2017 (full)  Community insights:  MDTs could also include physiotherapists, nurses, and general practitioners - Briggs et al 2018 (community)  A valuable point that is made in this report is the need for organisations to work together: “Integrated care systems, bringing together commissioners and providers of health and social care services with local authorities and other partners, intend to offer a more strategic outcomes-based approach to care.” – Care Quality Commission 2021 (community)  If an inter-professional/MDT has shared care plans and protocols involving joint decision making, and has routine meetings, then health and social care needs may be better met and outcomes improved - Trivedi et al 2013 (community, systematic review)  If healthcare and social care providers could be co-located, then this would help enable integration and develop the necessary relationships required to form new teams - Mackie & Darvill 2016 (community, systematic review)  If there is good/clear communication between healthcare and social care providers, then this could help maintain team relationships & team functioning - Mackie & Darvill 2016 (community, systematic review)  If management support and leadership is provided, then  this would help facilitate the implementation of integrated health and social care teams - Mackie & Darvill 2016 (community, systematic review)  If sufficient additional resources & capacity are provided, then this could be a key enabler to the implementation of integrated health and social care teams - Mackie & Darvill 2016 (community, systematic review)  If there is a national policy for integrated health and social care for adults, then this could be a key enabler to the implementation of integrated health and social care teams - Mackie & Darvill 2016 (community, systematic review)  "There is increased recognition of a need for services to bring together a range of professionals and skills from across the health and social care sectors. This integration of care is intended to benefit the service user. Integrated care systems are being implemented; it is vital that assessments of these systems are carried out in a systematic and meaningful way." – Kelly et al 2020 (community, umbrella review)  “Challenges to measuring the effects of the integration of care included the identification, and appropriate measurement of, a wide range of mechanisms and outcomes which may be impacted across conceptually diverse interventions [22, 28, 33]. Comparisons between studies included within the reviews were considered difficult due to the heterogeneity of outcomes and study design [22, 24, 28]. Few studies reported within study comparison, for example, usual care versus integration of care, making it difficult to determine effectiveness [22]." - Kelly et al 2020 (community, umbrella review)  If health and social care teams shared IT systems, then this could be a key enabler to the implementation of integrated health and social care - Mackie & Darvill 2016 (community, systematic review)  Integrated working (care homes): CHs’ readiness to work with health-care staff (e.g., leadership, previous history of collaboration); availability of structured assessment and care plans; involvement of a HCP to support change and reinforce learning; organisational endorsement; financial remuneration; staff incentives. Having a GP as part of the care delivery team and access to a wider array of services. Enabling NHS staff and care home staff to **co-design** how they work together to improve residents’ health care - Goodman et al 2017 (community, realist evaluation)  If there is a requirement and payment for dedicated care time as part of a social care provider’s job plan or service specification, then this could have a legitimising function and enable staff to focus on ‘working with’ rather than ‘doing to’ CHs [prisons] – Goodman et al 2017 (community, realist evaluation)  If structured/standardised, comprehensive assessments could be adopted, followed by development of care plans and person-centred goals and coordination of social care delivery activities, all with the engagement of a MDT, then prisoners’ social care needs would be better met - Chadborn et al 2019 (community, realist review)  If integrated social care could be provided by having MDTs who worked together to ensure comprehensive assessment, and case management, then health and social care needs could be better met – Briggs et al 2018 (community, umbrella review)  If MDTs could include social workers, physiotherapists, nurses, and general practitioners, then the health and social care needs of prisoners could be better met - Briggs et al 2018 (community, umbrella review)  If prisons could consult service users and consider factors outside physical health when tailoring services, then prisoners with lived experience would be able to provide an insight into which SC services may be most beneficial for them and which they would most utilise - Briggs et al 2018 (community, umbrella review)  Focusing on the consumer, developing a shared vision of care, leadership involvement, service provision across the boundaries, adequately resourcing the arrangement, developing novel arrangements or aligning with existing relationships, and strengthening connections between sectors - Winters et al 2016 (community, umbrella review)  A common vision and purpose, shared between leaders in a system, to work together to meet the needs of people who use services - Care Quality Commission 2018 (community)  Effective and robust leadership, underpinned by clear governance arrangements and clear accountability for how organisations contribute to the overall performance of the whole system - Care Quality Commission (2018)  Strong relationships, at all levels, characterised by aligned vision and values, open communication, trust and common purpose - Care Quality Commission (2018)  Joint funding and commissioning - Care Quality Commission (2018)  The right staff with the right skills - Care Quality Commission (2018)  The right communication and information-sharing channels - Care Quality Commission (2018)  A learning culture - Care Quality Commission (2018)  Single points of access to provide one point of contact for people and professionals, from which they could be referred to the most appropriate team, based on the person’s needs - Care Quality Commission (2018)  “health and social care leaders need to recognise the interdependencies of their sectors, and plan together for a sustainable system workforce” and focusing on preventative care - Care Quality Commission (2018)  A move from an activity-based funding model towards population-based budgets that encourage collaboration between local systems. In support of this, the national leaders (NHS England, NHS Improvement, the Department of Health and Social Care, and the Ministry of Housing, Communities and Local Government) must work with the Local Government Association and the Association of Directors of Adult Social Services, who should be involved as equal partners, to encourage and enable this change - Care Quality Commission (2018)  “This review suggests that a movement towards focusing on integrated models of care for multimorbidity is likely to offer some positive effects over usual care, such as reduced depressive symptoms, particularly if models have a theoretical basis, are comprehensive (including patient education, self-management structured interprofessional collaboration and professional support) and are targeted at those with high morbidity." – Frost et al 2020 (community, umbrella review)  A single, joint, nationally agreed framework for measuring the performance of how organisations collectively deliver improved outcomes for older people - Care Quality Commission (2018)  Local leaders should agree joint workforce plans, with more flexible and collaborative approaches to staff skills and career paths - Care Quality Commission 2018 (community)  Developing shared aims across organisations: "Locally, health and social care leaders … develop and agree a shared understanding and definition of what integrated care means for their population in their local area, and then work towards delivering this shared aim.” - Care Quality Commission 2016 (community)  What works in implementation of integrated care programs for older adults with complex needs: Trusting MDT relationships, clarity in terms of respective roles, joint ownership and accountability, close collaboration, sharing knowledge. Outcomes: Better coordination and continuity of patient care; reduced health system utilisation; improved patient health; improved patient and caregiver experience – Kirst et al 2017 (community)  Prevention: joint development of an agreed methodology at a national and local level across health and social care that would enable identification of those most at risk of hospital admission or 'deterioration' - Care Quality Commission 2016 (community)  Reviewer’s note: Alludes to preventative approach to healthcare which could be used to make prisons more suitable for older adults. Authors refer to assisted interventions or 'self-management' and 'Tai Chi' (which could be introduced into prisons). The review is good with regards to how comprehensive it is and considers factors such as quality of life and 'patient involvement and satisfaction with care' which is valuable as it takes a different perspective to just the health factors and can help to determine who the treatments work for best and how. However due to the lack of integration of health and social care in previous reviews, it is difficult to determine how well this works and how it could be incorporated into a prison environment – Dawson et al 2020  A focus on person-centred outcomes and meaningfully involving service users in decisions regarding care needs and care planning - Care Quality Commission 2016 (community)  Provision of adequate information about what is available for service users and their families/carers - Care Quality Commission 2016 (community)  A single clinical record system for an area, with information sharing agreements to support electronic communication and coordination - Care Quality Commission 2016 (community)  Responsive and coordinated care: a frailty register to support staff in improving how vulnerable frail people are identified – can support prevention - Care Quality Commission 2016 (community)  Care delivered by healthcare and SC teams working together, rather than being seen separately by each service - Care Quality Commission 2016 (community)  ‘Care navigators’ to offer support with case management, multi-disciplinary team meetings and complex referrals - Care Quality Commission 2016 (community)  “One of the main principles of integrated care is person-centredness” – Stoop et al 2020 (community)  Review staff numbers and skill mix regularly to ensure they are sufficient.  Ensure all involved with the person’s care are familiar with how they prefer support to be given; where possible, the same people are supporting the individual; if the same staff are not available, ensuring there are good handover arrangements; using the same independent advocate where possible - NICE 2018 (community, recommendations)  Ensure that prisoners are informed in advance if staff will be changed and any changes to care and support are negotiated with the prisoners - NICE 2018 (community, recommendations)  Involve voluntary and community sector organisations with expertise in equality and diversity issues to ensure that they can deliver services that meet the needs and preferences arising from: gender, sexual orientation, disability, ethnicity, religious and cultural practices – NICE 2018 (community, recommendations)  To support collaborative working between services, commissioners and managers should consider a local policy for sharing information relevant to people's care within and between services, and joined-up policies, processes and systems – NICE 2018 (community, recommendations)  Pointers to help organisations put NICE guidelines into practice:  1. Raise awareness through routine communication channels, such as email or newsletters, regular meetings, internal staff briefings and other communications with all relevant partner organisations.  2. Identify things staff can include in their own practice straight away.  3. Identify a lead with an interest in the topic to champion the guideline and motivate others to support its use.  4. Conduct a baseline assessment against the recommendations to determine gaps in current service provision.  5. Develop an action plan, with the steps needed to put the guideline into practice.  6. Review and monitor how well the guideline is being implemented.  - NICE 2018 (community, recommendations)  'Some common causes in the literature for OOCs [omissions of care] leading to adverse events include … delegation of tasks, lack of education in care staff, complex resident care needs, and urgent or unexpected situations that interfere with regular care.' - Ogletree et al 2019 (community – nursing homes) |
| 1.5 **Agreements on roles and responsibilities** | |
| C: Despite the introduction of Care Act 2014, there is confusion among prison staff regarding who is responsible for identifying and assessing prisoners’ social care needs.  M – resource: The local lead for social care in each prison^7^ ensures that formal agreements, including a memorandum of understanding^7^ (MOU) are established between LAs, service providers and the prison, that the agreements are kept up to date, and that all staff are aware of respective roles and responsibilities.  M - response: Staff: Confidence in own role  O: Clarity regarding who is responsible for what. Consistency of care. Duplication avoided. Increased job satisfaction while preserving professional identity. Prisoners’ needs more likely to be identified, assessed, and met adequately and in a timely manner. | *This had originally been subsumed within the staff training/awareness section but the team later felt it should be presented in a separate CMO, in part due to stakeholder practitioner feedback. However, after further iterations it was subsequently incorporated within the core principle of integrated care/joint working* |
| **1.6 Physical environment** | |
| If cells and facilities throughout the prison were appropriately adapted to prisoners’ needs, if there were designated dorms for prisoners with disabilities or chronic illness which were closer to common living areas, and if prisoners were given enough time to respond to activities related to prison drills, then they would be able to be as independent as possible while maintaining their dignity – Scottish Government 2022 (full); CLINKS & RECOOP 2021 (full); Age UK 2019; Dillon et al 2019 (full); Du Toit et al 2019 (full); Peacock et al 2019 (full); Care Quality Commission & HM Inspectorate of Prisons 2018 (full); Stewart 2018 (scoping); Turner et al 2018 (scoping); HMIPS 2017 (full); Munday et al 2017 (full); Lee et al 2016 (full); Mistry & Muhammad 2015 (full); Trotter & Baidawi 2015 (full); Rodriguez 2014 (full); Hayes et al 2012 (full); Williams 2013 (full); Senior et al 2013 (full); Sumner 2012 (full)  Physical environment CMO  C: Much of the prison estate is antiquated and not designed with the needs of people with disabilities or other social care needs. While adaptations have been made to some prisons many remain unsuitable for people who have difficulties with mobility or self-care. This restricts prisoners’ ability to retain their independence and dignity.  M – resource: Appropriate adaptations to cells and prison facilities, such as handrails, ensuring that floor surfaces are even and non-slippery, brail signage, wider cell doors and wider corridors. There could also be specially designated blocks for prisoners who have disabilities or chronic illness, or dorms located close to communal areas.  M – response: Prisoners feel safer, valued, and less excluded.  O: Prisoners maintain their independence and dignity, and gain confidence to get around their cells and elsewhere in the prison safely. Prisoners are better able to access activities and resources and to socialise, thereby becoming more likely to develop and maintain positive relationships. | Summary of key recommendation regarding facilities: More space for on-site assessment and intervention; investment in accessible and adaptable facilities – Scottish Government 2022 (full)  If access to and safety in showers was optimised, then prisoners with mobility problems would be better able to have regular showers, maintain their personal hygiene and independence - Williams 2013 (full)  If prisons were adapted with lifts, and prisoners with mobility problems were encouraged to use them, then they would be more independent and more able to access prison facilities, take part in activities, and socialise - Williams 2013 (full)  If prisoners with mobility problems could be located on the lower floor and lower bunks, then they could maximise their independence and be more likely to be active and socialise - Williams 2013 (full)  If prison chairs and other seating took account of prisoners' conditions, then those with problems such as back pain would be more willing to get out of their cell into communal areas, take part in activities, and socialise - Williams 2013 (full)  If problems with the prison's physical environment (twisting stairways and uneven surfaces) could be resolved, then prisoners with mobility problems would be able to get outside for exercise and recreation - Williams 2013 (full)  If prisons had shower cubicles adapted for use by prisoners with social care needs, and baths with grab handles, then those prisoners would be able to retain some independence and maintain their personal hygiene - Williams 2013 (full)    If staff could ensure that prisoners with social care needs are able to access the emergency bell without getting out of bed, then those prisoners would be able to safely call for help in an emergency - Williams 2013 (full)  If prisons could make simple changes to the environment such as coloured cell doors, pictures, calendars, handrails, and non-slippery floors, placing older inmates on lower bunks, then this could assist in maintaining orientation, coping, and independence in inmates with dementia - Mistry & Muhammad 2015 (full)  If prisons could have in-housing units close to dining halls for prisoners with dementia, and if they were given more time to respond to activities related to prison drills, then this would allow them to function independently while maintaining their dignity - Mistry & Muhammad 2015 (full)  If simple adaptations could be made to cells, such as soft lighting, large clocks, and brail signage, then prisoners with visual impairment or dementia would be able to cope better - Munday et al 2017 (full)  If prisons could have ground floor wings with wider access, corridors and cells, then those who use mobility aids such as walkers/ Zimmer frames or wheelchairs would be more appropriately located and able to be more independent, mobile and active – Munday et al 2017 (full)  If benches could be installed at strategic points around the prison, then prisoners would feel encouraged to walk more which would increase their independence and well-being - Care Quality Commission & HM Inspectorate of Prisons 2018 (full)  If adaptations such as handrails or ramps could be added to cells, then prisoners would be better able to get around them - Care Quality Commission & HM Inspectorate of Prisons 2018 (full)  If cell doors could be specially adapted cell doors so they were wide enough for wheelchairs to pass through freely, then prisoners who need them would be less likely to be stuck in their cells and more able to take part in social activities safely – Stewart 2018 (full)  If prisoners with mobility problems were provided with four-wheeled walkers with built in padded seats, then they would feel more comfortable resting and would be more able to collect their own meals (tray can be carried on the walker), which would increase their independence and reduce over-reliance on prisoner buddies - Care Quality Commission & HM Inspectorate of Prisons 2018 (full)  If occupational therapists could assess every cell to establish need, then issues such as high bunks and low toilets could be addressed (e.g., plinths to raise the toilets where no other mechanism available) - Care Quality Commission & HM Inspectorate of Prisons 2018 (full)  If prisons each had a stock of communal wheelchairs, then they could be distributed more quickly when needed - Care Quality Commission & HM Inspectorate of Prisons 2018 (full)  “The SPS needs to review the prisons estate to ensure that the facilities that are provided not only match the needs of the population, but also those of the service providers working with them. Communal areas and in particular cells need to be designed in such a way to ensure that they provide the space to allow nursing and care staff to maintain their professional standards and operate safely.” - Her Majesty’s Inspectorate of Prisons in Scotland (HMIPS) 2017 (full)  If the prison environment was adapted to be more suitable for older prisoners, then they would be able to make use of their environment safely - Her Majesty’s Inspectorate of Prisons in Scotland (HMIPS) 2017 (full)  Ensure that prisons meet their Public Sector Equality Duty under the 2010 Equality Act, “for example by ensuring that accommodation is suitable for prisoners with mobility or other support needs and by providing age specific regimes for prisoners” – Age UK 2019  Access to and safety in bathing facilities could be improved by providing shower cubicles and baths with grab handles - Williams 2013 (full)  If older prisoners were given priority at mealtimes and had permission to take their food to their cells, then they can have more time to finish their meals and meet their nutrition needs - Sumner 2012 (full)  If prisons could use clearer signposting or highly contrasting-coloured lines on the floor to key locations within the prison, then this type of support could be helpful to prisoners who have difficulties navigating the prison environment - Dillon et al 2019 (full)  If the built environment in prisons could be adapted to make it more suitable for prisoners with social care needs, then those who were less able would retain some independence while also being able to access facilities and engage in social interactions - Lee et al 2016 (full)  If prisons had sufficient mobility aids, then this would be a simple and relatively low-cost change which would enable prisoners with social care needs to remain more independent and access the prison without need for additional support - Lee et al 2016 (full)  If prisons had in-cell provisions such as meals and library books, then this would help reduce the need for those with mobility issues to travel to hard-to-access facilities - Lee et al 2016 (full)  If prisons conducted physical environment checks (guidance provided by CLINKS/RECOOP) to ensure that all areas were accessible for those with mobility, visual and hearing impairments and respond to its findings, then the social care needs of prisoners with such impairments would be better met - CLINKS & RECOOP 2021 (full)  If prisons had locate flat/low policies in place, then this would ensure that people with mobility needs are accommodated on ground floors or bottom bunks thus enabling them to be more independent to manage in their cells, to get out of their cells, to be more active, and to socialise more - CLINKS & RECOOP 2021 (full)  Access to single cell options where possible for prisoners with social care needs - CLINKS & RECOOP 2021 (full)  Benches could be installed at strategic points around the prison, which may encourage prisoners to walk more and thereby supporting independence and well-being - Care Quality Commission & HM Inspectorate of Prisons 2018 (full)  If the prison environment was made more suitable for prisoners with social care needs in terms of architecture, design, accessibility, temperature, and noise etc., then social care needs would be better met - Turner et al 2018 (scoping)  If appropriate resources were available to staff providing social care to prisoners with incontinence at night, for example somewhere to shower inmates and availability of fresh bedding, then the social care needs of incontinent inmates would be better met, and staff would be able to fulfil their role more effectively - Turner et al 2018 (scoping)  “Environmental issues, including the design, layout, and facilities of the buildings in the prison estate, frequently present challenges for both staff and prisoners. Many buildings are old and were designed for younger, fitter prisoners than those housed in them now. For example, one prison … was housed within a medieval castle that was also a historic “listed building,” which meant that alterations such as installing lifts or widening cell doors to allow for wheelchair access simply could not be made. Even newer prisons are not necessarily suitable for older people. In our current study, one governor described the prison (which was built in 1979) as “not fit for purpose”” Turner & Peacock 2017  “prison officials should identify low-cost ways for current facilities and facility policies to be redesigned and retrofitted to meet constitutional and other legal standards of physical and mental health care for this population. For example, support handles in showers can reduce the risk of falls, as can a prohibition on ankle shackles for incarcerated adults over a certain age or at a certain level of functional impairment. Housing assignments that prioritize access to dining halls, exercise facilities, and health services can also improve outcomes by minimizing barriers to proper self-care” – Psick et al 2017 (full)  Almost one-fifth (19% of 100 older prisoners) used a stick, Zimmer frame or tripod to move around within the prison. 11% said they had fallen in the past month. From interviews with 27 older prisoners: Reliance on stairs meant that physical access to particular areas of the building was difficult for older prisoners with mobility problems. Bunk beds were not accessible for older prisoners which sometimes meant they had to sleep on the floor. Older prisoners commented that the exercise facilities available were not suitable for their needs – Senior et al 2013 (full) NOTE, BEFORE CARE ACT.  The authors refer to “the considerable body of international evidence suggesting that prison environments are unable to consistently cater for the needs of older prisoners with physical incapacities” and that “There are implications for planning in relation to prison structure and facilities for the growing population of older inmates, particularly in relation to the identified problem areas such as bunks, staircases and bathroom facilities” - Trotter & Baidawi 2015 (full)  If sufficient resources (such as incontinence pads, spare clean clothing and bedding) were made available then prison staff would be better equipped to support prisoners with personal hygiene needs – Williams 2013 (full)  Each prison could have a stock of communal wheelchairs that are kept well-maintained. These could then be distributed more quickly as and when needed - Care Quality Commission & HM Inspectorate of Prisons 2018 (full)  Consider designated dorms for prisoners with disabilities or chronic illness so that they can be housed closer to common living areas, and give them enough time to respond to activities related to prison drills etc. - Rodriguez 2014 (full)  If prisons could make environmental changes and ensure appropriate provision of programmes then older prisoners would feel supported and more likely develop occupational and vocational skills – Peacock et al 2019 (full)  If the prison environment is adapted to the social needs of particular groups of prisoners such as older prisoners or those with dementia, (such as improved accessibility, creating a ‘dementia friendly environment’ tailored to visual perception difficulties, improving the comfort of prisoners, providing peer support) then these prisoners may be less reliant on staff support and medications - Peacock et al 2019 and Brooke et al 2020 (scoping)  There are examples of good practice which address mobility issues. For example, in one prison those with mobility issues are accommodated on the ground floor and a stair lift has been installed to allow these people to reach the medical unit on the first floor - Joyce & Maschi 2016  The document acknowledges that people in prisons have complex needs and the importance of understanding the factors that impact on health and social care outcomes for people in prison, including the specific needs of vulnerable cohorts including older and/or disabled individuals. Also notes the importance of taking account of the physical environment, staffing levels, and regime - Her Majesty’s Government, NHS England, Ministry of Justice 2018  If NOMS conducted a comprehensive analysis of prisons’ physical compliance with disability discrimination and age equality laws, then it could determine which prisons are not able to make the adaptation necessary to hold older or disabled prisoners - House of Commons Justice Committee 2013 (full) NOTE THIS WAS PRIOR TO THE CARE ACT |

**2. Identification of social care needs in prison**

| **Consolidated/refined if-then statements; CMOs** | **Original if-then statements / details / examples / ‘nuggets’ of information** |
| --- | --- |
| **2.1 Screening tools / early identification** | |
| If prisons used standardised and validated screening tools to screen for social care needs upon arrival to prison, and to screen for learning difficulties and related problems, then prisoner’s health and social care needs can be better identified and translated into care plans - Forsyth et al 2022 (full); stakeholder workshop (Feb 2022); Tucker et al 2021 (full); NHS England and NHS Improvement 2021; Peacock et al 2019 (full); Care Quality Commission & HM Inspectorate of Prisons 2018 (full); Skarupski et al 2018 (full); Barry et al 2017 (full); Barry et al 2015 (full)  Identification CMO  C: The identification of prisoners with social care needs is not always conducted in a systematic way on admission to prison, over the course of an individual’s sentence, or prior to release. Additionally, some prisoners are reluctant to acknowledge their social care needs due to fear of appearing ‘weak’ or vulnerable, while others may not have the capacity to request help without the assistance of an advocate.  M – resource: A standardised and validated screening tool to identify social care needs upon arrival to and prior to release from prison. Although standardised, the process allows for a person-centred approach with open-ended questions, enabling and encouraging prisoners to acknowledge any difficulties they may have with mobility, ADLs, or self-care, and staff are trained to conduct the screening in a sensitive and respectful way. The process includes screening for mental health problems, learning difficulties, dementia, mild cognitive impairment, and trauma alongside general health and social care needs.  M - response (prisoners): Willingness and enablement to acknowledge problems; sense of dignity; feeling valued; feeling safe.  M – response (staff): awareness of prisoner needs and history; seeing the prisoner as a disadvantaged person rather than a ‘criminal’; increased confidence in role and responsibilities.  O: Individual’s health and social care needs are identified in a sensitive and timely manner; enhanced preventative care and support. | Importance of timeliness including assessment of needs at reception. An individual might come into prison with an existing social care package, but this might not be known or considered once they enter prison depending on the quality of the information in the community / how well it’s documented - Stakeholder workshop, February 2022  If prisons developed standardised processes of the screening and assessment of needs upon entry to prison, then the health and social care needs of older prisoners could be better met - Peacock et al 2019 (full)  If staff could address prisoners’ social care needs on arrival, then this would help ensure a smooth transition into the prison - Care Quality Commission & HM Inspectorate of Prisons 2018 (full)  If prisons used validated screening tools, then they could better identify the health and social care needs of older prisoners on entry - Peacock et al 2019 (full)  If a specialist screening and assessment tool, such as the Older Prisoner Health and Social Care Assessment and Plan, could be routinely used, then the complex and multiple needs of older prisoners would be better identified – Tucker et al 2021 (full)  If a standardized, supplemental intake assessment tool for older prisoners could be used, perhaps one incorporating variables from the Minimum Data Set used to screen all nursing home residents, then vital baseline health status data can be collected and facilitate development of an appropriate care plan - Skarupski et al 2018 (full)  “It is important that a prison-wide approach to LDD screening and information sharing is co-ordinated, both for system efficiency and to avoid the stress of repeat screening and assessments” - NHS England and NHS Improvement 2021  Timely screening could reduce the risk of social care needs going unrecognised and could prevent self-harm and suicide - Forsyth et al 2022 (full)  Include screening prisoners’ mobility especially for those who use a wheelchair or a walker and where cell doors may be too narrow - Care Quality Commission & HM Inspectorate of Prisons 2018 (full)  There is significant scope to improve the information and care provision arriving with people at reception into prison – Levy et al 2018  “It is argued that the act of asking about trauma can lead to more thoughtful referrals, whilst beginning a process of institutionalising trauma within discussions. … Importantly, by the Prison Service screening to identify trauma histories, this helps to identify individuals who need support and to respond to those needs accordingly.” – Bradley 2021  If prisons could identify prisoners with PADL disability, then this could help to determine a critical point of intervention (e.g., onset of PADL disability) and present opportunities for prevention of depression and suicidal ideation such as accommodations for PADL disability – Barry et al 2017 (full); Barry et al 2015 (full)  If prisons developed an integrated assessment of health, mental health, and social care needs on initial arrival, then this would help remedy the lack of information that prisons receive and to develop a system of continual referral and provision - House of Commons Justice Committee 2013 (full) NOTE THIS WAS PRIOR TO THE CARE ACT  “both self and social stigma are likely to play an influential role in self-reporting. Stigmatised or hidden disabilities (such as learning disabilities or mental health) are at risk of going undetected, resulting in an under-estimation of both need and lack of provision of specialist support” – Scottish Government 2021 |
| **2.2 Active case finding** | |
| If prisons promoted active case finding in addition to screening for social care needs on admission, and if the system anticipated needs rather than reacting retrospectively, then prisoners who developed needs at any point during their imprisonment and/or whose willingness to acknowledge a need for help had changed, would be better identified – Levy et al 2018; Skarupski et al 2018 (full); Tucker et al 2018 (full)  Active case finding CMO  C: Some people enter prison without any social care needs but develop them over time as their health or functioning deteriorates. These emerging needs are not always detected due to lack of systematic, formal process to identify such needs throughout a person’s time in prison. Furthermore, some prisoners are reluctant to seek support due to fear of appearing ‘weak’ or vulnerable and/or a lack of knowledge about the support they are entitled to.  M - resource: As part of an integrated health and social care system, active case finding is encouraged. Staff and prisoners are educated to be aware of, to anticipate, and to be sympathetic to social care needs and prisoners’ rights to receive support if they are struggling. Once identified, support needs can be reported to a social care lead and adequately assessed.  M – response: Care receivers: feel valued and validated; confidence.  M - response (staff): Empathy and respect.  O: Greater awareness among staff and inmates of emerging or changing needs; reduced risk of newly emerging needs being overlooked; increase in proportion of people with needs being identified. | If prisons promoted active case finding in addition to screening for social care needs on admission, then prisoners who developed social care needs at any point during their imprisonment and/or whose willingness to acknowledge a need for help had changed, would be better identified - Tucker et al 2018 (full)  If periodic standardized assessments of older incarcerated individuals or brief evaluations after an event (e.g., fall, injury, health event) were used then changes in care needs can be measured over time and aid advanced care planning upon probation or release into the community – Skarupski et al 2018 (full)  “Reasonable adjustment should be a core plank of working within an equalities framework, central to social care assessment processes and accessibility in a way that anticipates needs rather than reacts retrospectively, and considers social dimensions as well as the physical environment” – Levy et al 2018 |
| **2.3 Awareness/education/self-referral** | |
| If information on the Care Act was provided to prisoners and prison staff through easy-read posters and leaflets, and if local advocacy arrangements could be set up, then prisoners would be made more aware of their right to receive support, the availability of such support, and would be more likely to self-refer - Forsyth et al 2020 (scoping); Care Quality Commission & HM Inspectorate of Prisons 2018 (full); Di Lorito et al 2018 (scoping); NICE 2018 (community); Munday et al 2017 (full); Eadie et al 2017 (full); Anderson 2015; Williams 2013 (full)  Awareness CMO:  C: Social care needs are not well understood among prison staff, prisoners with social care needs are often unaware of their rights to support or are reticent to seek help due to fear of appearing vulnerable, and there is often a lack of advocacy.  M - resource: Provision of information on the Care Act through easy-read posters and leaflets; development of local advocacy arrangements; ensure individuals understand the information. Provision of an accessible self-referral system.  M – response: prisoners with social care needs feel validated in seeking support; prison staff gain more empathy and respect for prisoners with social care needs.  O: Increased awareness among staff and prisoners; increased empowerment; more self-referrals; fewer unidentified/unmet needs. | If easy-read pamphlets and posters could be distributed to explain social care, then prisoners and prison officers would be made aware of the availability of support and the ability to self-refer - Care Quality Commission & HM Inspectorate of Prisons 2018 (full)  If all prisoners were supplied with the “The Care Act and You" leaflet which explains what The Care Act is and how it applies in prison, then prisoners and officers would be more aware of their rights and what to expect – Munday et al 2017 (full)  If Care Act 2014 information posters and leaflets could be displayed in prison reception areas, then prisoners with social care needs would be better informed of their rights and more willing to request help if needed - Eadie et al 2017 (full)  If information could be provided on reception to prison of any services available for older prisoners and those social care needs, then this would increase the likelihood of their needs being identified - Eadie et al 2017 (full)    If prison staff could promote a respectful and safe environment for prisoners with social care needs, respond compassionately and anticipate needs, then prisoners will feel safer and more willing to seek help when they need it – Care Quality Commission & HM Inspectorate of Prisons 2018 (full)  If local advocacy arrangements are in place for older or vulnerable prisoners, then their social care needs are more likely to be identified and met. This could be internal to the prison, or external advocacy services including from LAs - Forsyth et al 2020 (scoping)  Barrier: “ageing prisoners being poorer self‐advocates than the younger inmates” - Di Lorito et al 2018 (scoping)  If accessibility of social care could be improved by allowing prisoners to self-refer, providing advocates, and reducing eligibility criteria, then prisons would be better able to identify social care needs - Anderson 2015 (full)  If prison staff were given basic awareness training to recognise emerging social care needs, then prisoners’ needs would be more likely to be detected at the appropriate time - Di Lorito et al 2018 (scoping)  “Awareness-raising is required for staff and prisoners to develop understanding of disability and working/living with diversity, including the accumulative effects of exclusion” – Levy et al 2018  Consider that some prisoners may feel uneasy about disclosing their need for help when first admitted to prison, and others will lack insight into their condition – Tucker et al 2018 (full)  People in prison need to be able to self-refer for health and social care in a way that will promote outcomes and address inequalities arising through ill-health or disability – Levy et al 2018  If prison staff treat older prisoners with respect and took notice of needs that prisoners bring to their attention, then this could address some of their social care needs by allowing them to have positive interactions and provide them with the opportunity to maintain their personal hygiene/toilet needs - HM Inspectorate of Prisons for Scotland 2017 (full)  If prison staff did not have to apply the 'sameness' principle to prisoners with social care needs, then these prisoners would be more likely to access services needed for any physical disability or incontinence needs, thereby promoting equivalence of care to those outside of prison – Williams 2013 (full)  Community insights  Information:  In line with the Care Act 2014, LAs must provide information about care and support services including: the types of care and support available; how to access care and support, including eligibility criteria. LAs should ensure that information about care and support services is widely and publicly promoted and provide information about the circumstances in which independent advocacy is available. Plain language and personalised communication; check people understand the information  - NICE 2018 (community, recommendations)  Ensure are open channels of communication. Support if have communication needs - in line with the Accessible Information Standard - this could include advocacy support, independent interpreters to enable communication in a language they can readily converse in, a carer, communication aids, additional time to understand and process information - NICE 2018 (community, recommendations) |

**3. Assessment & care planning**

| **Consolidated/refined if-then statements; CMOs** | **Original if-then statements / details / examples / ‘nuggets’ of information** |
| --- | --- |
| **3.1 Initial assessments** | |
| If holistic and multi-disciplinary assessment processes are adopted and if prisoners are enabled to be actively involved in their assessments, individual goal setting and co-produced flexible care plans, with the support of advocates where necessary, then this would lead to more appropriate assessment, greater self-efficacy and motivation, and better working relationships – Stakeholder Workshop (Feb 2022); Care Quality Commission 2021 (community); Hagos et al 2021 (full); Caiels et al 2021 (community); Forsyth et al 2018 (full); Levy 2018; NOMS 2016; Forsyth et al 2015 (full); NICE 2018 & 2019 (community)  Initial assessments CMO  C: Initial needs assessments are sometimes conducted by staff lacking knowledge about social care problems or how to communicate and collaborate effectively with those in need. Some prisoners are reluctant to acknowledge their needs due to past trauma or fears of being seen as ‘weak’ or vulnerable. Trauma survivors are at risk of being retraumatised by the experience of being assessed.  M - resource: Development of person-centred assessment procedures, administered by specially trained, trauma-informed staff, whereby individuals are encouraged where possible to be actively involved in goal setting and co-produced care plans. Plans are designed to be flexible in anticipation of personal care, mobility, nutrition, activity, and other changing needs. Care receivers are encouraged to identify their strengths and assets to help shape services. Includes access to local advocates or family members where the individual is not capable of being meaningfully involved in assessment and planning.  M - response: (Prisoners): motivation; feeling valued; hope  O: More appropriate and holistic assessments; care plans are tailored to the individual and able to take account of changing needs; timelier provision of support; improved working relationships  Nutrition  If appropriate nutritional interventions could be adopted for older prisoners and others with social care needs, then they would be better able to manage and maintain their nutrition - Mohan et al 2018 (full); Robinson et al 2018 (full); Wangmo et al 2018 (full); Firth et al 2015 (full); Lorber et al 2013 (full); Jenkins et al 2012 (full); Sumner 2012 (full)  *CMO - amalgamated with the assessment/care plans CMO*  *C: Some prisoners struggle to manage and maintain their nutritional needs for several reasons including difficulty getting to the dining hall, insufficient time to eat meals, physical problems with eating for example due to ill-fitting dentures, inadequate portions, and menus which are not based on individual medical, religious, or ethical dietary requirements.*  *M - resource: Nutritional interventions (such as customised menus, sufficient time to eat meals, options to eat in own cells) built into care plans and tailored to meet the needs of the individual.*  *M - response:*  *O: Prisoners’ nutritional needs are more likely to be met, promoting general health and well-being.* | Important to consider the individual’s capacity to be involved in the decisions / plans – stakeholder workshop, February 2022  If prisoners are actively involved in their assessments, then their care plans could be personalised, and they could indicate how they wished to have their needs met - Care Quality Commission & HM Inspectorate of Prisons 2018 (full)  If care plans are current and person-centred with clear review dates, then prisoners’ social care needs would be more likely to be met - Care Quality Commission & HM Inspectorate of Prisons 2018 (full)  If prisoners could be involved in planning their own social care needs and setting individual goals, then social care interventions would be more successful - Forsyth et al 2022 (full)  If systematic assessment and care planning procedures are in place, then social care needs will be better assessed and met - Forsyth et al 2015 (full)  A survey of local authorities in England found that in many cases prisoners were involved in formulating their care plans - Tucker et al 2018 (full)  If prisons employ staff that can dedicate time and are trained to assess social care needs, using measures such as the OHSCAP, then prisoners can feel more comfortable in discussing their needs, improving ability to identify and meet specific needs - Forsyth et al 2017;2021 (scoping)  “The need for an independent advocate is indicated if the person would have substantial difficulty in being involved in the assessment or review process. This means the  person could not understand or retain relevant information, or use or weigh up information as part of the process of being involved, or be unable to communicate their views, wishes or feelings without substantial difficulty” - National Offender Management Service (NOMS) 2016  A personal care plan should be introduced for every ageing prisoner. “The care plan should highlight the specific care needs of the prisoner and how these should be met. Plans should be readily available to the relevant staff and should accompany the prisoner if they are moved to another location. A personal care plan should form part of the specification of contractual obligations with other parties who are involved in the management of prisoners for the Scottish Prison Service. In this way problems such as incontinence and the implementation of escort during medical treatment can be managed sensitively, appropriately and consistently to ensure that elderly prisoners receive humane treatment.” - Her Majesty’s Inspectorate of Prisons in Scotland (HMIPS) 2017 (full)  The means by which people in prison identify their needs and desired outcomes should be through a holistic and multi-disciplinary assessment process – Levy et al 2018  Nutrition  If prisoners could be educated regarding nutrition, then they would be more prepared to ensure they are meeting their own nutritional needs - Mohan et al 2018 (full)  If prisons introduced reduced calorie meals, then conditions such as diabetes may be easier to manage within prison - Firth et al 2015 (full)  If prison menus labelled the caloric content and incorporated garden produce into meals, then prisoners would be better able to ensure their nutritional needs are met - Firth et al 2015 (full)  If prisons offered small classes and training opportunities related to nutrition and gardening, then prisoners would be better able to ensure their nutritional needs are met while also engaging in purposeful activity - Firth et al 2015 (full)  Nutritional interventions could include giving prisoners who struggle to get around and/or to feed themselves priority at mealtimes, giving them the option to have their meals in their cells - Sumner 2012 (full)  Should address physical barriers to eating, such as ill-fitting dentures – Sumner 2012 (full)  Customise menus based on health, religious, and ethical dietary requirements - Wangmo et al 2018 (full)  Give those who struggle with their nutrition the option to order appropriate food online or receive it from family/friends– Wangmo et al 2018 (full)  If prisons employed nutritionists, then older prisoners’ nutrition needs could be assessed and meals tailored to these needs – Robinson et al 2018 (full)  If prisoners with diabetes could be educated regarding the carbohydrate content of food, then they could have more control over their food choices and ensure they are receiving appropriate nutrition - Lorber et al 2013 (full)  If dieticians worked with prison chefs in preparing menus and meal plans for prisoners with diabetes, and advice offered regarding appropriate choices based on availability in the prison, then those prisoners would be more likely to have their nutritional needs met and experience an improvement in their overall wellbeing - Jenkins et al 2012 (full)  Community insights:  Trauma-informed approaches  “Trauma informed care is used in many good practice examples and takes a person-centred approach to someone’s history” - Care Quality Commission 2021b  Supporting independence / person-centred care:  Use self-defined strengths, preferences, aspirations and needs as the basis on which to provide individualised care and support. Actively involve service users in all decisions. LAs must provide independent advocacy to enable people to participate in: needs assessment, care planning, the implementation process, and review, where they would otherwise have substantial difficulty in doing so. Needs assessment should involve the person in discussions and decisions about their care and support; promote independence; respect dignity; be transparent. LAs should ensure that: the person is given details of the process, timescale, nature and purpose of the needs assessment; that they can have someone they choose to be present at the assessment; the assessment uses up-to-date information and documentation about the person - NICE 2018a (community, recommendations)  Focus on the individual’s needs and involve them in their care and plans, ensuring they are well informed to make decisions (e.g., how to access support groups, whether telecare would be suitable etc); Ensure individual’s care plans are accessible to them; involve a care coordinator and ensure staff delivering care are well integrated and coordinated - NICE 2015 (community)  Have a named coordinator as the first point of contact and to contribute to the assessment process, liaise and work with the prisoner and all services involved with the person, including voluntary services, and ensure referrals are made and actioned - NICE 2018a (community, recommendations)  Clear information about involvement from others (e.g., peer supporters, advocates) - NICE 2018a (community, recommendations)  Flexible plans to accommodate changing priorities, needs and preferences - NICE 2018a (community, recommendations)  Include and address the specific needs of prisoners in relation to equality and diversity issues - NICE 2018a (community, recommendations)  If a strengths-based approach is used in the assessment process, then prisoners can be supported to understand their needs, realise what they can do, and how to best use their skills and networks, to achieve their outcomes - Caiels et al 2021 (community, systematic review); Social Care Institute for Excellence 2015 (community)  If social care practitioners adopted the five quadrant KVETS model (Knowledge, Values, Ethics, Theory, Skills) to guide themselves and service users along a series of steps and questions that encourage respectful conversations, then this could mobilise person-led and person-centred practice - Caiels et al 2021 (community, systematic review)  The 3 Conversations Model in South Gloucestershire Council:  “The Conversational approach to assessments enables us to fulfil our preventative and wellbeing duties under the Care Act 2014 as well as working with people who are eligible for care and support. Conversations are as follows:  1. Listen & Connect- aim to connect people to resources that already exist either in their own networks or within their community to meet their outcomes.  2. Work intensively in a crisis- aim to ‘stick to someone like glue’ while they overcome a change in their lives, and make short term plans, because you never plan long-term in a crisis.  3. Build a good life- aim to support someone to make long-term plans to meet their outcomes, which includes consideration of eligibility.  The 3 Conversational approach started with 2 innovation sites in February 2019 it has now grown to 5 sites and has been a successful approach in terms of:  • Quicker response times.  • 70% of new referrals were concluded with a Conversation 1 or Conversation 2  • Reduction in existing waiting times.  • More streamlined processes.  • Greater worker satisfaction.” - Caiels et al 2021 (community, systematic review) |
| **3.2 Review / audit for prisoners already identified as having social care needs** | |
| If person-centred assessment of prisoners could occur at least twice a year with clear review dates, then subsequent care plans would be more appropriate and responsive, and those who develop SC needs while imprisoned would not be overlooked - Forsyth et al 2022 (full); Care Quality Commission & HM Inspectorate of Prisons 2018 (full); NICE 2018 (community); Skarupski et al 2018 (full); Tucker et al 2018 (full); Walsh et al 2014 (full)  Reviews/follow-ups CMO  C: Social care needs change over time but many prisons lack any systematic follow-up procedures to monitor needs. Furthermore, some prisoners are reluctant to speak out and seek additional support due to fears of appearing ungrateful or being seen as ‘weaker’ or more vulnerable.  M - resource: As part of an integrated health and social care system, person-centred assessment includes follow-up reviews twice yearly as standard with clear review dates, and more frequently when necessary, in response to individuals’ changing needs. Everyone is given user-friendly information about their right to receive social care support in a respectful and dignified manner, and their right to self-refer for follow-up assessments.  M – response: Care receivers: encouraged to ask for help if needs change; feel valued; motivation  O: More appropriate and responsive care plans; reduced risk of changing needs being overlooked. | If care plans are current and person-centred with clear review dates, prisoners’ social care needs are more likely to be met - Care Quality Commission & HM Inspectorate of Prisons 2018 (full)  If there is regular audit / checking of care plans and input to prevent slippage of delivery, then effectiveness and appropriateness of assessments and subsequent care plans will improve - Forsyth et al 2022 (full)  If care plans are current and person-centred with clear review dates, prisoners’ social care needs are more likely to be met - Care Quality Commission & HM Inspectorate of Prisons 2018 (full)  If older prisoners' social care needs are assessed regularly (e.g., every 6 months), then care plans can be reviewed to take account of changing needs – Walsh et al 2014 (full)  Follow-up assessments should be more than twice yearly, when necessary, in response to changing needs  Community insights:  Plans should be regularly reviewed, include information on how and when reviews should be conducted, contingency and crisis planning - NICE 2018A (community, recommendations) |
| **3.2 Monitoring and oversight** |  |
| If prisons had clear processes to oversee referral and assessment procedures with a multidisciplinary team, and if care plans could be monitored by service managers, then the quality of care plans, assessment, and treatment could be kept at a high standard - Care Quality Commission & HM Inspectorate of Prisons 2018 (full) | If care plans are monitored by service managers who report to service commissioners on trends in care package delivery, then this would ensure that delivery performance and quality had appropriate governance - Care Quality Commission & HM Inspectorate of Prisons 2018 (full) |

**4. Provision of care and support in prison**

| **Consolidated/refined if-then statements; CMOs** | **Original if-then statements / details / examples / ‘nuggets’ of information** |
| --- | --- |
| **4.1 Peer supporters** |  |
| If prisons adopted formal peer support systems with appropriate job descriptions, training and supervision, then this could improve prisoners’ career prospects and self-esteem, reduce burden on staff, enhance prisoner/staff relations and better meet prisoners’ social care needs - Forsyth et al 2021; Lennox et al 2021 (full); Perry et al 2021 (full); Flanigan 2020; House of Commons Justice Committee 2020 (full); Peacock et al 2019 (full); Greenwich Prisons Social Care; described in a literature review by Walton et al 2019; Brooke & Jackson 2019 (full); Du Toit et al 2019; Lee et al 2019 (full); Care Quality Commission & HM Inspectorate of Prisons 2018 (full); Levy et al 2018; NICE 2018A (community); Stewart 2018 (full); Munday et al 2017 (full); Lee et al 2016 (full); Prisons and Probation Ombudsman 2016 (full); Grohs 2015 (full); O’Hara et al 2015 (full)  Peer support CMO  C: Peer support can be provided to prisoners, but not always in a formal, supervised, or appropriate way.  M – resource: Provision of a formal peer support system with appropriate suitability checks, training, job descriptions, supervision, and ongoing support and safeguarding for caregivers, and safeguarding for care receivers. Peer supporters are provided with education and training to identify and report social care needs and to provide agreed, person-centred support in a respectful and dignified way. Try to match caregivers to care receivers.  M – response: peer supporters: a sense of purpose; self-esteem.  M – response: care receivers: a sense of dignity; connection to peers; self-esteem  O: Prisoners receive more appropriate and timely care/support and feel safer and less stigmatised.  Peer supporters benefit from engaging in meaningful activities, learning new transferable skills, and increased confidence, motivation, and prospects.  Less strain on prison staff. Improved relationships among prisoners, and between staff and prisoners. | Training for peer supporters could include a National Care Certificate equivalent which could be developed, perhaps with the support of a third sector organisation such as RECOOP which offers support and advice in implementing such a scheme for prisons - Munday et al 2017 (full)  If prisons had social care peer support schemes with formal job applications, suitability and security checks, job descriptions, training, supervision, and oversight, then support provided to prisoners could be recorded as part of regular care plan reviews and prisoners would be better supported - Care Quality Commission & HM Inspectorate of Prisons 2018 (full)  If prisoner carers were appropriately trained, monitored, assessed, and not overly relied upon, then benefits would be seen for the carer (skills, altruism) and the person being cared for (helping with day-to-day activities) - Prisons and Probation Ombudsman 2016 (full)  If prisons utilised a 'buddy system' to assist with meeting social care needs of older prisoners, then there may be less pressure on prison staff and increased likelihood of the needs of more prisoners being met – House of Commons Justice Committee 2020 (full)  If peers could act as Healthcare Representatives, with appropriate training and robust procedures in place including adequate planning and management to avoid adverse incidents and abuse of responsibilities, and with a dedicated full-time manager, then they could help with some social care needs including supporting access to services, reminding them about appointments and acting as advocates. It could also benefit the peer by increasing self-esteem, self-advocacy, and a sense of responsibility, and could improve services within the prison and improve communication between other prisoners and care providers – Lennox et al 2021 (full)  If prisons developed a Buddy Support Service to provide prisoners with mentoring and National Care Certificate equivalent training, then the basic social care needs of prisoners could be more readily met and the peer supporters would be less likely to feel stressed by their role. (This could be developed with the support of a third sector organisation such as RECOOP which offers support and advice in implementing such a scheme for prisons) - Munday et al 2017 (full)  If prisons had prisoner helper/buddy schemes, including adequate training, then some of the (non-personal) social care needs of older prisoners could be met - O’Hara et al 2015 (full)  If an overarching policy for peer-support programmes could be developed in prisons, then adequate training and supervision would be given to peer-supporters and better care provided - Grohs 2015 (full)  If prisons adopted the use of peer support, then this could be an efficient and cost-effective way of delivering social care within prison - Lee et al 2016 (full)  If peer supporters were provided with adequate training and support, then they would be better able to manage their role and be safeguarded against psychological stressors - Stewart 2018 (scoping)  If peer supporters were provided with adequate training, then prison officers would be more likely to work collaboratively with them - Stewart 2018 (scoping)  If peer supporters were provided with adequate training, then they would gain confidence to provide better informed care whilst knowing the limits of their role and when to ask for formal help - Stewart 2018 (scoping)  If prisoners were screened and trained to provide support to older prisoners with dementia, then more person-centred, one-on-one care cold be delivered while providing peer supporters with valuable rehabilitative skills, and creating a sense of community in the prison environment - Du Toit et al 2019 (full)  If social care in prisons was delivered by a team of experienced social care staff assisted by a team of trained Care and Support Orderlies (existing prisoners), then this would help clients better address their domestic and personal social care needs - Greenwich Prisons Social Care; described in a literature review by Walton et al 2019  Trained orderlies could assist social care staff in meeting domestic and personal social care needs - Greenwich Prisons Social Care; described in a literature review by Walton et al 2019  If peer supporters are provided with adequate training, ongoing support, and psychological safeguarding, then they will be better able to manage their role, gain the confidence to provide better informed care whilst knowing the limits of their role and when to ask for formal help, and prison officers will be more likely to work collaboratively with them - Stewart 2018 (scoping)  Review the approach to peer supporters considering the judgement that the State cannot shift its duties for care onto other prisoners whilst recognising that for many people in prison, caring for or being cared for by a peer is a positive experience. Explore issues of care service/carer registration and training. We recommend introducing group peer mentoring programmes run by disabled people or disabled ex-offenders – Levy et al 2018  Good practice examples for supporting older people in prison include peer support schemes – Flanigan 2020  If 'buddy' prisoners were trained and supported throughout their support of other prisoners, then they would feel confident in taking a person-centred approach - Brooke & Jackson 2019 (full)  If appropriate prisoners could be trained to help identify and address other prisoners’ social care needs, then needs would be better met - Forsyth et al 2021 (full)  Overall, the programmes were reported to have a generally positive impact, with the transformative effect upon the prison overall and prisoner peer supporters most frequently reported, and peer support particularly commended for the hospice programmes. Peer support provides a positive impact for social care programmes in prison. – Lee et al 2019 (full)  If older prisoners had access to buddying systems, then they could build relationships with others and be supported to meet their social care needs – Peacock et al 2019 (full)  Community insights  Match peer supporters to prisoners, considering: the prisoner's care and support needs, and their cultural, religious and communication needs; the carer’s knowledge, skills and experience – NICE 2018A (community, recommendations)  If prisoners have eligible needs that could be met by prisoner peer supporters, this should be discussed with and understood by them at the care planning stage. Provide training opportunities for prisoners who are interested in becoming peer supporters - NICE 2018A (community, recommendations) |
| **4.2 Purposeful/meaningful activity** | |
| If the skills and experience of the volunteer sector and prisoners could be used to codesign and coordinate tailored work opportunities and other activities for prisoners from underserved populations, then those with social care needs would have equal access to appropriate purposeful activity, and increased confidence and well-being - CLINKS & RECOOP 2021 (full); Tucker et al 2021 (full); Orellana et al 2020a and 2020b (community); Rowe et al 2020 (full); Brooke 2019 (full); Peacock et al 2019 (full); Care Quality Commission & HM Inspectorate of Prisons 2018 (full); NICE 2018A (community); Connell et al 2017 (full); Eadie et al 2017 (full); Ellen et al 2017 (community); Lee et al 2016 (full); Baidawi et al 2016 (full); Hellman et al 2016 (full); Field et al 2014 (community); Saunders 2013 (full); Hayes et al 2012 (full)  Purposeful activity CMO  C: There is often a lack of suitable educational, vocational, and other meaningful activities for prisoners with social care needs, and where they do exist some prisoners have difficulties in accessing them.  M - resource: Provision of tailored work, training, and other activities including day care support, codesigned by the voluntary sector and prisoners, and ensuring ease of access.  M – response (prisoners): feel valued and included; motivation; empowerment; sense of purpose; mastery; pride; hope.  O: Prisoners have equal access to purposeful activity; learn new skills; improved prospects for self-improvement including work prospects; reduced social isolation; improved autonomy; improved general well-being. | If the skills and experiences of the voluntary sector and prisoners themselves could be used to develop tailored activities, then prisoners with social care needs would have fairer and equal access to purposeful activity and time out of their cells - CLINKS & RECOOP 2021 (full)  if prisoners with social care needs were offered structured activities such as 'light' work, then they would be more occupied and mobile, and have more chance to get out of their cells and to socialise - Hayes et al 2012 (full)  If prisoners are enabled to work and earn money doing something meaningful to them, then this would motivate them, help them get through the day, enable them to phone friends and family - Naessens 2020 (full)  If specific gym sessions were provided for prisoners with social care needs, concentrating on mobility rather than muscle building, then their mobility would improve and they would have greater opportunity to get out of their cells and to socialise - Hayes et al 2012 (full)  If prisons could ensure appropriate provision of programmes, then older prisoners would feel supported and able to develop occupational and vocational skills to meet their social care needs – Peacock et al 2019 (full)  If prisons could offer specialist services for older prisoners (and those with social care needs generally), such as over 50s health clinics and low impact gym sessions, then social care needs could be better identified and met - Lee et al 2016 (full)  If inmates with cognitive disabilities are taught essential skills (numeracy/literacy) in the form of daily living or practical skills, then then will be more open to learning – Rowe et al 2020 (full)  If activities aimed at promoting the physical, mental, and emotional well-being of prisoners with SC care needs could be developed, then this would help develop/maintain independent living and confidence in approaching support services - Tucker et al 2021 (full)  If programmes were targeted to older prisoners vocational (age-appropriate work, taking into account health issues), physical (age-appropriate exercise), and socio-emotional needs (buddy schemes), then they would experience lower levels of distress in prison - Baidawi et al 2016 (full)  If prisons could offer Day Centres for prisoners with social care needs to promote meaningful activity, then the prisoners would have a safe space in which to interact with others, and make good use of their time in a safe environment - Eadie et al 2017 (full)  If prison Day Centres could create a prisoner job role of 'activity co-ordinator', then this would help to tailor activities specifically to the needs of those with social care needs whilst also providing employment - Eadie et al 2017 (full)  The range of work and activities available within prisons needs to be tailored to the physical abilities of an older population and with a particular emphasis on ensuring that 'social time' is built into the daily regime - Her Majesty’s Inspectorate of Prisons in Scotland (HMIPS) 2017 (full)  Social workers and prison officers need to be pro-active in ensuring and supporting access to purposeful activities that reflect individual strengths, interests – Levy et al 2018  Purposeful activities should include developing skills for work and employment on release irrespective of protected characteristics - Levy et al 2018  If prisons developed ‘Older Prisoners Active Living Groups’ [and similar for prisoners generally with social care needs], then this would help ensure a range of appropriate activities and information sessions such as talks on social care support, resettlement, and activities promoting the constructive use of leisure time – Saunders 2013 (full)  Disabled prisoners should have the same access to and payment from involvement in purposeful activities as all prisoners - Levy et al 2018  Peer support should be explored as a purposeful activity - Levy et al 2018  Educational activities for inmates with mental health issues or LDDs could be adapted to their needs. This would help them to be more engaged in learning, feel motivated to change, be more able to pay attention and learn, experience feelings that they may have lacked due to their mental health (mastery, empowerment, sense of control from personal choices, inclusion in a group, adaptive peer relationships). Teacher attributes would include classroom observation, being adaptive, being active, building rapport, avoiding distractions, careful direction of attention. Lesson attributes would include relatable content, group goals, scaffolding, drawing as homework, varied mediums, group and individual choices– Hellman et al 2016 (full)  If offenders with PDO's [“personality disordered offenders”] were offered interventions oriented towards achievement of a pro-social identity, target skill deficits that impact successfully on employment and social relationships, or provide practical assistance to access pro-social roles, then they could better and more confidently engage in work, training, education of volunteering – Connell et al 2017 (full)  If social groups and work groups could be offered to older prisoners, then this would provide them with a safe place away from boisterous and unsettled younger prisoners, and provide them with a purpose which could motivate them to complete activities – Brooke 2019 (full)  If prison initiatives were not age-defined, then older prisoners who wished to attend initiatives within the prison would not feel excluded - Brooke 2019 (full)  If appropriately checked prisoners could conduct low level maintenance of equipment such as tightening screws and replacing rubber parts on walking frames and sticks, then this would optimise the condition of the equipment while at the same time providing meaningful activity - Care Quality Commission & HM Inspectorate of Prisons 2018 (full)  Community insights  Day care support for prisoners with mobility restrictions who felt socially isolated could reduce the risk of declining independence and wellbeing, and improve QoL, levels of social participation and meaningful activity - Orellana et al 2020b (community)  Day care support could include practical help with social care needs such as hairdressing and maintaining hearing aids, which would help prisoners with aspects of their personal care - Orellana et al 2020b (community)  ADS (adult day service) centres support the health, nutritional, social, and daily living needs of adults with functional limitations in a group setting during daytime hours. Review of 61 studies found several benefits of ADS in terms of wellbeing (e.g. 'self-esteem, perceived physical health, engagement) but noted some limitations with these studies. Benefits with regards to functioning such as 'preventing falls, gait and motor skills' were also identified. Evidence for improvements in mental well-being – Field et al 2014 (community)  Age UK Day Centres: Attendees have a personalised plan for participating in activities; provide practical assistance and a chance to socialise, with support from trained staff and volunteers; range of activities offered includes music and singing, quizzes, gentle exercise, arts and crafts; hot lunches are provided; some centres offer additional services such as assisted bathing, hairdressing, foot care and mobile supermarkets – Age UK 2022 (community)  Provision of self-care resources in day care support could improve autonomy - Ellen et al 2017 (community)  Exercise sessions in day care facilities could help maintain mobility and alleviate depression - Orellana et al 2020b (community)  Day care support could feature social prescriptions by which primary care professionals refer prisoners with social or practical needs to non-clinical services thereby helping to promote improved outcomes - Orellana et al 2020b (community)  Day care providers should offer a choice of activities that are led by the prisoner's needs and interests to motivate them and promote independence. Recognise that preferences are not fixed and may change – NICE 2018A (community, recommendations)  “Interventions for pre-frail and frail older adults should include multi-component exercises, including in particular resistance training, as well as aerobic, balance and flexibility tasks” - Jadczak et al 2018 (community) |
| **4.3 Relationships** |  |
| If prisoners with social care needs could have adequate contact with family and friends and had access to social activities, and if there were positive staff relationships, then they would find it easier to cope, feel less isolated, feel encouraged to develop and maintain personal relationships, and less be likely to reoffend after release - Kenkmann et al 2022 (scoping); Hwang et al 2021 (full); Peacock et al 2019 (full); Albertie et al 2017 (full); Grohs 2017 (full); HMIPS 2017 (full); Scottish Prison Service 2017 (full); Flynn et al 2016 (full); Maschi et al 2015 (full); Adorjan and Chui 2014 (full); Bartlett et al 2014 (full); Hayes et al 2012 (full)  Relationships CMO  C: It is crucial that people in prison can develop positive relationships while serving their sentence and also have sufficient contact with family and friends outside prison so that they can reduce the negative impacts of feeling socially isolated and have a better chance of being successfully re-integrated once they are released back into the community. This is particularly important for some prisoners who have social care needs together with other problems such as learning difficulties, mental health problems, or older age. The amount of contact prisoners are usually allowed varies, for example 4 hours per month in the USA but as few as 2 hours per month in the UK. As a result many prisoners are unable to re-build or maintain important relationships.  M - resource: As part of an integrated health and social care system, there are arrangements in place which a) allow prisoners adequate contact with family and friends (more than the standard amount if this is what they need and want to maintain their social network). This could include video visitations; and b) provide accessible activities such as tailored clubs and buddying systems to enable social interaction and development of positive relationships with other prisoners. Positive staff relationships are also important particularly for those who have no other contacts.  M response (prisoners): feel less isolated and despondent; more included; more valued  O: Prisoners with social care needs are more likely to develop and/or maintain personal relationships with family and fellow prisoners, less likely to feel isolated, depressed, or suicidal, thus will find it easier to cope while in prison and are more likely to have a good social support network on and after release from prison. | If prisoners with social care needs could have adequate contact with friends and family, then they would find it easier to cope with prison life and their personal relationships would be better maintained – Hayes et al 2012 (full)  If prisoners with SC needs were allowed additional visiting hours, then this would help them maintain relationships with family and friends - Kenkmann et al 2022 (scoping)  If activities were provided that were accessible for older prisoners such as a club specifically for older prisoners, then this would help them to develop and maintain personal relationships within the prison - HM Inspectorate of Prisons for Scotland 2017 (full)  If prisoners were given the choice to use video visitation then this would make family contact easier (reducing travel and waiting time) and save money on security (prisoner transport, staff) - Grohs 2017 (full)  Prisoners should be supported to maintain positive contact with their families and arrangements made to encourage family visits - Her Majesty’s Inspectorate of Prisons in Scotland (HMIPS) 2017 (full)  “Recognize the value of relationships to be people in prison and develop innovative approaches to support people in prison to maintain external relationships, contact with family and friends outside of prison” – Levy et al 2018  If prisoners with family relationship needs were in facilities as close to their families as possible, then this would facilitate improved family interactions and their ability to maintain relationships with their children - Bartlett et al 2014 (full)  If offenders could access family support, then they would be less likely to re-offend – Flynn et al 2016 (full)  If positive familial ties are fostered in prison and upon release (ensuring family understands offender, emphasis on reducing shame), then ex-offenders will get the support they need to find employment and desist from crime and drug use - Adorjan and Chui 2014 (full)  If resources could be allocated to trauma-informed programs that re-establish family and community relationships, then this may help address human rights issues and promote overall well-being – Maschi et al 2015 (full)  If programs could provide links to family in a safe and supportive prison environment, then this would encourage hope and optimism and feelings of connectedness and community - Maschi et al 2015 (full)  "These social programs might include family and volunteer prison visiting and service programs, pen pal programs, intergenerational caregiver support services, and intergenerational supportive televisiting services" - Maschi et al 2015 (full)  Given the body of evidence on the positive effect family contact has on hope for the future, reintegration and post release care - adoption of best practice would minimise family estrangement in future plans for housing those in custody with social care needs – Scottish Prison Service 2017 (full)  If strategies could be used to minimise psychological distress and social isolation, similar to strategies used for community-dwelling older people such as improved access to audio-visual and digital technologies for increasing meaningful phone calls, then this would improve social support and connectedness – Hwang et al 2021 (full)  If prisoners are supported to preserve their connectivity (i.e., interpersonal relationships with family and friends outside prison), then this could help protect prisoners from psychological harm and substance abuse by providing emotional and material resources – Albertie et al 2017 (full)  *Rival statement: If connectivity is enforced, then this might lead to higher levels of distress for some prisoners who may not respond well to continued reminders of isolation from social networks and disruption of pre-incarceration trajectories -* Albertie et al 2017 (full) |
| **4.4 Safety / specialised wings** | |
| If prisoners were given the option to be on a prison wing dedicated to prisoners with social care needs, with appropriately adapted physical environments, then prisoners could be more independent, active, and able to socialise safely – CLINKS & RECOOP 2021 (full); Turner et al 2018 (full); Her Majesty’s Inspectorate of Prisons in Scotland (HMIPS) 2017 (full); Monday et al 2017 (full); Hayes et al 2012 (full) | If prisoners were given the option to be on a prison wing dedicated to prisoners with social care needs, with appropriately adapted physical environments, then prisoners could be more independent, active, and able to socialise safely – CLINKS & RECOOP 2021 (full)  If prisons could have a ground floor wing with wider access, corridors and cells, then those who need mobility aids such as walkers/ Zimmer frames or wheelchairs would be more appropriately located and able to be more independent, mobile and active – Munday et al 2017 (full)  If prisoners with social care needs could be offered the choice to be housed on a designated wing for prisoners in need of social care, then this would reduce risk of bullying - Hayes et al 2012 (full)  If older adults were segregated from younger adults in prison to units more suited to their needs, and if prisoners are consulted and given choice in this process, then their social care needs could be better met – Hagos et al 2021 (full)  “The SPS and Scottish Government need to agree a joint approach on the location and management of these prisoners. The prisoners involved in this survey were generally of the view that an age specific population was not desirable. Nonetheless it seems inevitable that a rising population of ageing prisoners in SPS custody will begin to present distinct and more concentrated needs and challenges. It may be that alternative accommodation should be considered, which maintains the level of security that a prison sentence requires, but is suited to the provision of appropriate health and social care for this population.” - Her Majesty’s Inspectorate of Prisons in Scotland (HMIPS) 2017 (full)  If older prisoners were housed separately from younger prisoners, then it may be easier for prisons to meet their specific social care needs as there would be less worry about intimidation leading to older prisoners not feeling safe or not receiving adequate nutrition – Turner et al 2018 (full)  If older prisoners were brought together within the wider population, then this could ensure their needs were best met in the right environment and with the right services on hand – Ministry of Justice (MoJ) 2013 (full) NOTE THIS WAS PRIOR TO THE CARE ACT  *But note, they also say that “The integration of prisoners of different ages in prisons has potential benefits for all elements of the prison population and management.”* |
| **4.5 Out of hours provision** | |
| If prisons could provide out-of-hours access to social care support, including overnight, then prisoners’ SC needs could be attended to in an emergency, and they would be at lower risk of falls - Forsyth et al 2020 (scoping); Care Quality Commission & HM Inspectorate of Prisons 2018 (full)  *Merged the suggestion of out of hours provision with the ‘resources’ part of the CMO about collaborative working/MDTs* | If sufficient out-of-hours provision is available for prisoners with dementia and SC needs, e.g., during the night, then their needs will be better met - Forsyth et al 2020 (scoping)  If prisoners could have access to social care support overnight, then they would be less likely to fall and remain unattended to - Care Quality Commission & HM Inspectorate of Prisons 2018 (full) |
| **4.6 Consistency of care** | |
| If prisoners receive support from regular staff rather than agency or temporary staff, then staff would become more familiar with individual prisoners’ needs and more likely to build a rapport, which would better facilitate the care and support provided - Care Quality Commission & HM Inspectorate of Prisons 2018 (full)  *Added as an outcome in the collaborative working/MDTs CMO* | If prisoners receive support from regular staff rather than agency or temporary staff, then staff would become more familiar with individual prisoners’ needs and more likely to build a rapport, which would better facilitate the care and support provided - Care Quality Commission & HM Inspectorate of Prisons 2018 (full) |

**5. Release**

| **Consolidated/refined if-then statements; CMOs** | **Original if-then statements / details / examples / ‘nuggets’ of information** |
| --- | --- |
| **5.1 Pre-release courses/preparation /planning** | |
| If prisons ran pre-release courses with tailored content for those with social care needs, with everyone being made aware of the courses, and if resettlement planning began at the earliest opportunity and meaningfully involved prisoners in the process and with integrated working between agencies, then this would reduce prisoners’ fears and anxieties prior to release and make resettlement more successful by better meeting care needs and ensuring continuity of care - Stakeholder Workshop (Feb 2022); Lares 2022 (full); NHS England and NHS Improvement 2021; Rowe et al 2020 (full); Age UK 2019; National Probation Service 2019 (SCoR); Di Lorito et al 2018 (scoping); Levy et al 2018; Young et al 2016 (full); Ethridge & White 2015 (SCoR); Forsyth et al 2015 (full); O’Hara et al 2015 (full); Maschi et al 2014 (full); Maschi et al 2013 (scoping); Adorjan and Chui 2014 (full); Saunders 2013 (full); Senior et al 2013 (full)  If prisoners were enabled to maintain life skills and to ‘give back’ (e.g., volunteering/community work) and/or by being healthy role models, then they might feel less shame about their incarceration and be more likely to successfully reintegrate on release - Kenkmann et al 2022 (scoping); Lares, 2022); Maschi et al 2014 (full)    If prisons provided user-friendly, personalised guidance notes for prisoners with social care needs prior to release and a personalised pathway document upon release, and if a ‘nodal point’ could be set up for those returning to the community providing probation, counselling and aftercare services, then the transition should be less stressful and more successful – Munday et al 2017 (full); Raghavan 2013 (full)  If appropriate relationships with good communication, sharing of information and better integration could be developed between prisons and other agencies such as the probation service and LAs, and the MoJ published up to date information about which local authorities controlled prisons, then prison officers would be able to share prisoner release information more effectively and prisoner transition would be more successful with their long-term social care needs being met - Robinson et al 2022 (full); Levy et al 2018; Pearmain et al 2016 (full); van Dooren et al 2016 (full); Young et al 2016 (full); Valmaggia et al 2014 (full)  If prisoners could receive appropriate education delivered by trained specialists in collaboration with voluntary organisations to develop referral pathways, then prisoners with social care needs would develop the necessary skills to help them more successfully reintegrate into the community - Kenkmann et al 2022 (scoping); Cochrane et al 2021 (scoping); Rowe et al 2020 (full); Du Toit et al 2019 (full); Raghavan 2013 (full)  If contact with probation services occurred earlier rather than just before release from prison, then it is more likely that a suitable place to live would be found and this would reduce prisoners’ anxieties and fears - Hayes et al 2012 (full)  If progress of released prisoners is monitored to ensure they have access to the appropriate services, then needs will be better met and re-entry will be more successful - Forsyth et al 2015 (full)  Original CMO for pre-release planning (incorporates the 7 statements above)  C: Many people face several barriers to successful resettlement once released from prison, including having nowhere to stay, no training or employment lined up, very little money or clothing, and no medication or immediate access to other services. Those without a supportive social network and/or with health and social care needs are at even greater risk if they do not receive the support and aftercare they need to help them cope, as are survivors of PTSD. Some people may have managed in prison but are unable to function in the community, for example, if they cannot manage stairs but are not housed in ground floor accommodation.  M - resource: A programme which involves release planning at the earliest opportunity and provision of education and pre-release courses including tailored content for those with social care needs. Release planning covers, at the very least, housing, food provision, welfare benefits, medication, social and healthcare, training/education and employment. Prisoners are involved in the planning process and enabled to maintain or learn life skills, including training in the use of technology where applicable. For employment, education programmes in prison focus on basic work life skills, particularly for prisoners with disabilities, managing expectations, building up resumes and other job-specific skills training, including role play activities. Prisoners are given the opportunity to ‘give back’, for example by volunteering. They are provided with user-friendly, personalised guidance notes prior to release, and a care package and personalised pathway document upon release. ‘Nodal points’ are also set up for those returning to the community providing probation, counselling, and aftercare services. Arrangements for regular follow-up and self-referral for changing needs whilst in the community.  M - response: (Prisoners): motivation; positivity; pride; hope.  O: Prisoners develop or maintain the necessary skills to help them successfully reintegrate into the community; resettlement is more successful for more people by better meeting housing and other social care needs and ensuring continuity of care; reduction in prisoners’ fears and anxieties prior to release; reduction in feelings of shame about incarceration. | If older prisoners received pre-release education and training then this would help them to access post-release services and prepare them for the job market. Such education and training must address the varying context between prison and community - Hagos et al, 2022 (full)  If assessments and discharge plans are shared with community corrections, parole officers and other relevant community health-care providers, then this would provide good guidance to the whole process of prison-to-community transition - Hagos et al, 2022 (full)  If prison-based professionals are trained on how to support prisoners with complex needs, especially those who may be least likely to take initiative in asking for help, then their needs would be better met in prison and in preparation for release – Pasma et al 2023 (full)  If in-prison assistance by community-based professionals, such as parole officers, is promoted and funded, then they can operate in closer proximity to prisoners, which would increase the amount of contact and make them more capable to focus on prisoner needs in preparation for release - Pasma et al 2023 (full)  If prisoners were offered pre-release courses which catered for individual needs, then they would not have to rely on potentially inaccurate information from other inmates and consequently would be less likely to experience high levels of anxiety about their release - Di Lorito et al 2018 (scoping)  If release of older prisoners could be planned before release (incl housing, food provision, medication/healthcare, employment) then they would be better able to reintegrate into society, have better mental health, and be less likely to reoffend - Lares 2022 (full)  If plans for older prisoners’ release could be addressed early (i.e., on entry into prison) then this would reduce anxiety and fears about release - Forsyth et al 2015 (full)  If there was adequate release planning to ensure continuity of care before and after release, then re-entry to the community would be more successful - Forsyth et al 2015 (full)  If, as part of release planning, a care package is organised for prisoners with social care needs, then their needs will be better met post-discharge - Forsyth et al 2015 (full)  If progress of released prisoners is monitored to ensure they have access to the appropriate services, then needs will be better met and re-entry will be more successful - Forsyth et al 2015 (full)  If prisons had a pre-release program that educated prisoners on the social care they can access upon release such as the benefits they are entitled to, how to find employment or housing and self-care, then they will be more able to access support and to meet their own social care needs upon release - Maschi et al 2013 (scoping)  Release planning should include housing, food provision, medication, healthcare, training/education, and employment - Maschi et al 2014 (full)  If prisoners could be given job-specific or general skills training (conflict resolution, professional conduct etc), then employers would be more convinced of their ability to work well, and therefore more likely to hire them - Harley 2014 (full)  If prisoners could be given role play activities or training to help them explain their criminal history truthfully and evidence their change into a reliable worker, then employers would be more convinced of their ability to work and therefore more likely to hire them - Harley 2014 (full)  If prisoners’ basic (food, shelter) and criminogenic (anger management, SA) needs are targeted before or alongside gaining employment, then they would be more able to find and retain employment, and therefore less likely to reoffend - Harley 2014 (full)  As part of the Government’s rehabilitation programme, guidance should be developed on practical matters such as pension advice, housing and accessing health and social care ahead of release – Age UK 2019  Prisoners should be able to contribute meaningfully to plans that seek to support their transition back to the community – Levy et al 2018  “Some people will have needs that do not qualify them for social care services while they are in prison because the prison regime meets those needs as a matter of course. However, on returning to the community they may not manage as well and therefore healthcare professionals will need to consider the need for a further social care needs assessment referral as part of resettlement planning.” - NHS England and NHS Improvement 2021  If prisons established pre-release groups for older prisoners (and prisoners generally with social care needs) within 6 months of release to provide resettlement support and advice, then this could prepare prisoners for the transition from custody to the community which could make resettlement more successful and minimise anxieties about release – Saunders 2013 (full)  If programmes in prison and pre-release focus on skills and education training and target older offenders, then ex-offenders will feel more equipped to gain purposeful activity upon release – Adorjan and Chui 2014 (full)  In resettlement prisons, if discharge planning is effectively conducted, then this would assist in ensuring the social care needs of older prisoners are appropriately met on release – O’Hara et al 2015  If contact with probation services occurred earlier rather than just before release from prison, then it is more likely that a suitable place to live can be found and this would reduce anxieties and fears – Hayes et al 2012 (full)  “People leaving prison often experience difficulties in preparing for release. The local authority where the prisoner is located may carry out an assessment of the care and support they will need to support their release into the community. The Act will ensure that there will be continuity of care on release.” - Department of Health 2016  “People sent to prison may not be placed near their home and this can pose a challenge in facilitating continuity of care at time of arrival and release.” – World Health Organization 2020  If programmes for prisoners with cognitive disabilities were person centred and flexible, involved multidisciplinary teams, focused on individuals’ strengths, were culturally aware, and set realistic goals, then those prisoners would build skills that would reduce recidivism and help them reintegrate into the community – Rowe et al 2020 (full)  If prisoners with disabilities were provided with vocational services including basic work life skills, assistance with job retention, disability training, disability-related augmentative skills, then they may be more successful in securing and maintaining employment post-release - Baloch and Jennings 2018 (full)  (Prisoners released on medically recommended intensive supervision) Participants who were serving time were aware of some of the things they would need to learn when released from prison, including making food and going outside - Ethridge & White 2015 (SCoR)  (Prisoners with LDs) All key informants raised the critical importance of early planning and continuity of care as essential processes. Service contacts with sufficient ‘quality (one-on-one) time’ and proper training were highlighted as crucial to a positive transition experience. Aligning the timing and scope of support with the extent of the former prisoner’s re-entry needs was also considered pivotal for successful reintegration – Young et al 2016 (full)  (Prisoners with LDs) Identifying individual needs and providing appropriate support was seen to be most effective when agencies had sufficient time to work with a person, prior to their release from prison, to plan their transition into supported accommodation. Many agencies noted the importance of having a worker dedicated to client contact on the day of release, and of continued contact in the subsequent days and weeks in the community. Ongoing support can then be tailored to the individual’s circumstances and needs. System-level priorities include... developing adequate structure and governance that allows flexible service provision – Young et al 2016 (full)  People with LDDs: may have difficulty accessing rehabilitative and health services in prison and post release, communicating their needs to staff, and disclosing their disability. The person may have … difficulty understanding and retaining information relevant to care. Additional support may be needed to help people to apply for and access benefits. Lack of income and an inability to acquire it is likely to influence a person’s reintegration success, leading to significant stressors in acquiring fundamental needs such as food and housing and exacerbating the associated risk of reoffending - Ellem et al 2020 (full)  The NPS (National Probation Service) aims to improve staff’s knowledge of social care legislation, confidence in recognising potential social care needs, as well as confidence to make referrals to Local Authorities for assessment and support. This support may include advice to prevent, reduce or delay a social care need from developing – National Probation Service 2019 (SCoR)  NPS will seek to support, and improve, access to social care assessments. Achieving this goal will require timely information sharing and more robust engagement with Local Authorities, as well as working with prison colleagues to improve the transfer of care for individuals released into the community. This includes ensuring that care packages are reassessed based on an individual’s new environment - National Probation Service 2019 (SCoR)  The authors note that one prison participating in the study offered a pre-release training course to prisoners; however, not all prisoners interviewed at this prison were aware of the course's existence. The few prisoners who attended a pre-release course reported that some aspects were informative but that the information imparted was not tailored to older prisoners' needs – Senior et al 2013 (full)  Issue of housing: assessing the unknown – if prisoner is on lower floor needs might not be apparent. Approved premises may only be for 3 to 6 months; problem with those not moving to their own area; risk assessment/danger to selves. Short-sentence prisoners – needs may not be assessed because they’re in prison for a short time and may be released with even greater care needs – stakeholder workshop, February 2022  Joining up with other plans on release – think of the SC element alongside other needs they may have. Think about the interface; continuity of care and then the handover; how does handover happen or does it even happen. Consider combining pre-release plans with multi agency public protection (MAPP) - stakeholder workshop, February 2022  Consider risk periods for example re: drug-related deaths; issue of continuity of care. Release often happens on a Friday – this impact drug-related death as difficult for people to reach the support they need when key services shut down for the weekend - stakeholder workshop, February 2022  For prisoners transferring to LTC facilities: Developing valuable relationships with the correctional institutions could be achieved through an interdisciplinary team of key nursing home staff visiting the prisons. Viewing prison conditions will raise nursing home staff awareness about the environmental context from which their new residents are transitioning. Also, the visits will facilitate the building of important connections and promote future communication across these different institutional settings – Loeb 2013 (full)  Example of an initiative in the US (describe a case study): Taking services to the client / accompanying to appointments. Used a low-barrier, strengths-based approach responding to people's needs and supporting the achievement of their self-identified goals without judgement and utilized the evidenced-based frameworks of motivational interviewing (MI), harm-reduction, housing first (HF), and trauma-informed care (TIC) when applying therapeutic interventions and providing case management. In this case Roger had considerable cognitive impairment and the OT input focused on establishing daily routines; strategies to increase compliance with medication; planning for and preparing simple meals safely; money management; and home management (Detailed examples given of specific interventions). A key feature of this model of care is flexibility—of time, location, and spirit of service provision - Kannenberg and Conley 2020 (SCoR)  Examples of initiatives in UK:  a) Reablement service - a ‘short term free service’ and re-assessment.  b) The ‘reconnect service’ which supports people ‘through the gate’ who don’t normally meet the criteria for through the gate work; ensuring they get the correct referrals and engage in that, including housing, help with substance abuse etc. Identification and engagement pre-release is what makes it stand out. Working with them before they’re released. ‘transition period’. Registration with the relevant GP well in time before release is important but can be a challenge if unsure WHERE going to be released or not going back to own area.  c) ‘Weekly care planning meeting’ as part of their terms of reference - a MDT forum, discuss healthcare, social care needs etc. Makes a difference – enables the SC team to ask questions and get the info they need - stakeholder workshop, February 2022  If parole officers grounded their relationships with older parolees in social support/connection and respect, then re-entry would be more successful - Hughes & ten Bensel 2021  If special population training for parole officers was provided to help them address parolee needs and ease the distribution of resources for aging offenders, then officers would be better placed to support older parolees who in turn would be more likely to accept healthcare and self-care advice - Hughes & ten Bensel 2021  If there is good communication and sharing of information between prison services and local authorities, then appropriate care and support can be provided to those in prison, and support can continue when transitioning back to the community – Pearmain et al 2016 (full)  If interdisciplinary work occurs between appropriate agencies, released prisoners’ long-term social care needs will be more likely to be met - Di Lorito et al 2018 (scoping)  Prisoners with emerging mental health problems:  If there are effective links between prison and community services, with 'gate work' in the weeks prior to release and stable family support, then this could help reduce the risk of recidivism and social isolation – Valmaggia et al 2014 (full)  If each local authority had a single point of contact for the receipt of information about people moving into their area on release, then prison officers would be able to share information more effectively about prisoners’ social care needs - Robinson et al 2022 (full)  If clear timescales for referrals and responses were introduced, then the transition of prisoners on release would be more successful and their social care needs would be more likely to be met in a timely manner - Robinson et al 2022 (full)  If prisons developed good relationships with other agencies such as the probation service, then unanticipated social care needs of prisoners on release would be more likely to be identified and met - Robinson et al 2022 (full)  If the MoJ website published up-to-date information as to which local authority each prison falls into, then this would minimise uncertainty, the transition of prisoners on release would be more successful, and their social care needs would be more likely to be met - Robinson et al 2022 (full)  Prisoners with LDs:  If prisons could be involved in transitional service arrangements and facilitating organisations to be more involved with individuals prior to their release, then the social care needs of prisoners would be better met. This should include information exchange between prisons and supporting agencies – van Dooren et al 2016 (full)  Key informants highlighted the current system’s complexity and lack of integration as a potential barrier to effective service delivery and, ultimately, a successful transition from prison… A lack of formalised coordination between the justice system and disability service providers prior to release – Young et al 2016 (full)  There is significant scope to improve the information and care provision arriving with people at reception into prison and leaving with them at release – Levy et al 2018  If there is a lack of support and resources to encourage social care provision, then this creates a barrier to providing personalised social care upon release – Fox et al 2016 (full)  Appropriate education could include physical, health, academic, social, vocational, moral, spiritual, and cultural education - Raghavan 2013 (full)  If trained mentors, such as case workers from a third sector organisation, are used to assess the needs of and develop a pathway of referrals for soon-to-be-released prisoners, then reintegration back into the community would be more successful- Cochrane et al 2021 (scoping)  “Voluntary sector agencies have a key role to play in improving the lives of older prisoners both inside and outside the prison. More resources should be given to the third sector to provide these and other services to older prisoners." – Age UK 2019 |
| **5.2 Post-Release Employment** | |
| If employment placement programmes could be set up and special arrangements made with suitable employers, then prisoners would be more successful in securing and maintaining employment on release and employers would be more convinced of their potential - Ethridge et al 2020 (full); Baloch and Jennings 2018 (full); Durcan et al 2018 (full); Harley 2014 (full); Adorjan and Chui 2014 (full); Feist-Price et al 2014 (full); Raghavan 2013 (full); Durcan 2012 (full)    Post-release employment CMO  C: Reluctance among employers to hire ex-offenders significantly increases the risk of deterioration in mental and physical health, homelessness, and re-offending  M resource: Provision of employment placement programmes including input from employment specialists; special arrangements with and incentives for suitable employers who adopt pragmatic recruitment practices. Arrangements for follow-up or ongoing support if work circumstances or abilities change.  M - response: (Ex-offenders): motivation; dignity; pride; hope  M - response: (Employers): philanthropy  O: Prison leavers would be more successful in securing and maintaining employment, thereby reducing the risk of deterioration in well-being, homelessness, and re-offending; employers would be more convinced of the potential of ex-offenders | If people leaving prison have access to stable and good quality (less interchangeability of staff, higher income) jobs, then this would help reduce the risk of reoffending – Connell et al 2023 (full)  If practitioners collaborated with employers to modify workplace environments and reduce stigma, then former prisoners would be more likely to retain their jobs – Connell et al 2023 (full)  If practitioners encouraged people to apply for good quality stable jobs, addressed ‘soft’ and ‘life skills’ to help support sustaining a job, and supported wider needs related to health and living in under-served communities, then former prisoners would be more likely to keep their jobs and less likely to reoffend – Connell et al 2023 (full)  If there could be a tailored, wrap-around approach with joined-up commissioning to support prisoners with mental health problem through the prison gate and into the community, with employment specialists focusing on work outcomes and separate support and mentoring for other needs, then this could improve job outcomes - Durcan et al 2018 (full)  If the DWP could ensure that the Health and Work Programme provides effective support to people leaving prison and that contracts incentivise providers to adopt IPS principles, and avoid the use of conditions and sanctions on people with mental health difficulties, then this could help these individuals to find and retain paid employment - Durcan et al 2018 (full)  If employers could adopt pragmatic recruitment practices in the employment of ex‐offenders, for example by looking for a positive attitude and enthusiasm rather than focusing on qualifications while still providing opportunities for personal and career skills development prior to and after release, and if they could be aware and supportive of other needs such as accommodation, benefits, or family/relationship problems, then ex-offenders would be better able to find and retain paid employment - Durcan 2012 (full)  If there could be a review of all education, training and employment schemes in prisons, prioritising those that support people into actual employment and identifying opportunities for adopting IPS (Individual Placement and Support) principles for those who desire to engage in this, then this could help to achieve better outcomes for those seeking employment opportunities when they leave. This should include those with mental health difficulties - Durcan et al 2018 (full)  If prison leavers who are marginalised are offered supported employment, specifically evidence-based Individualized Placement and Support (IPS) to promote self-advocacy, then they will be more likely to secure and keep employment – Ethridge et al 2020 (full)  IPS (Individual Placement and Support) for offenders with severe mental illness:  Competitive Employment is the Primary Goal; Zero Exclusion: Eligibility Based on Client Choice; Integration of Employment & Mental Health Services; Attention to Client Preferences; Personalised Benefits Counselling; Rapid Job Search; Systematic Job Development; Time-Unlimited & Individualised Support – Hamilton et al 2015 (full)  Special arrangements could include quotas for recruitment in government jobs, bank loans and subsidies for self-employment, and follow-up of cases occurs upon release from prison - Raghavan 2013 (full)  Suitable employers could hire prisoners whilst still incarcerated with the help of intermediary agencies and the benefit of tax credits for hiring ex-offenders - Feist-Price et al 2014 (full); Holzer, Raphael, and Stoll (2003) |
| **5.3 General** | |
| If prison officers were given more time for meaningful interaction, then they may be more likely to address identified needs relating to discharge planning - Forsyth et al 2022 (full)  If older prisoners, and others with social care needs, could be allocated to prisons [resettlement prisons] near to their homes (or the courts dealing with their cases), then this would provide an opportunity to engage with resettlement services prior to release – MOJ 2013 (full) NOTE THIS WAS PRIOR TO THE CARE ACT | If prison officers were given more time for meaningful interaction, then they may be more likely to address identified needs relating to discharge planning - Forsyth et al 2022 (full)  If older prisoners, and others with social care needs, could be allocated to prisons [resettlement prisons] near to their homes (or the courts dealing with their cases), then this would provide an opportunity to engage with resettlement services prior to release – MOJ 2013 (full) NOTE THIS WAS PRIOR TO THE CARE ACT  “Although most reentry individuals initially are on some form of parole supervision, that arrangement at best emphasizes compliance and does not effectively address the multiple needs of these individuals. The crisis of change involved for this population calls for proactive social work service that is humanizing, responsive, and demonstrative that successful reentry is an achievable goal, however complex” – Kenemore 2014 (full) |
| **5.4 Special groups** | |
| If prisoners with intellectual or cognitive disabilities are provided with person-centred, flexible programmes with realistic goals run by an MDT, if prison staff are trained in caring for/understanding people with such disabilities, and if they are helped to find social support in prison, then these individuals could build appropriate skills leading to reduced recidivism and better community reintegration - Rowe et al 2020 (full); Boodle et al 2014 (full)  If prisoners with TBI (traumatic brain injury) are given skills to recognise how their condition affects their attentional, emotional, and behavioural abilities and are provided with adaptation and compensation techniques for TBI resultant deficits, then prisoners could maintain focus, plan ahead, manage responsibility, and deal with stressful or confrontational situations in an appropriate way and successfully reintegrate into society including employment or education - Linden et al 2021 (full)  If prisoners with learning difficulties could be taught life skills and have a personal learning plan which specifies educational activities that should be undertaken during their sentence, then this could help ensure a more successful re-integration into society with increased attendance at education and vocational training activities – Coates 2016 (full); Koo 2016 (full)  If prisoners had vocational councillors and could use staff-led, formatted manualized vocational group programs to provide education about job searching, modified to the individual prisoner, involving technique practice opportunities with a reflective discussion, then this would allow prisoners to better tailor their responses to their unique circumstances and help improve prisoner confidence and vocational outcomes post-release - LePage et al 2013 (full)  If additional in-prison vocational rehabilitation and employment programs are implemented for inmates with physical or cognitive disabilities (such as interpretation or reading services to aid in achieving and sustaining employment), then they groups may be more likely to utilise such services and better able to build connections and find employment prior to release to aid the re-entry process - Baloch and Jennings 2021 (full)  If there was timely planning of transition for prisoners with IDs, with support systems in the community being set up prior to their release and ensuring continuity of care/support, then they would more successfully reintegrate into the community and be less likely to re-offend - van Dooren et al 2016 (full); Murphy & Barnoux 2015 (full)  Special groups CMO (combines the 6 statements above re special groups)  C: People with physical, learning, or cognitive disabilities are more likely to experience problems re-settling back into the community and finding employment after being released from prison, especially if they have not been equipped with the necessary skills.  M - resource: Prisoners with physical, learning, or cognitive disabilities are offered person-centred and flexible vocational and employment programmes with realistic goals run by MDTs. They are taught life skills and have a personal, co-designed learning plan which specifies the educational activities to be undertaken during their sentence. They are given skills to recognise how their condition affects their attentional, emotional, and behavioural abilities and are provided with adaptation and compensation techniques. Vocational councillors provide education about job searching where applicable, modified to the individual prisoner, involving technique practice opportunities. Links are established between in-prison and community support programmes. Support is arranged prior to release with individuals being followed-up as needed.  M response (prisoners): Confidence about educational and employment prospects; motivation; pride; acceptance of strengths and weaknesses  O: Prisoners plan more realistically, manage responsibility, and deal with stressful or confrontational situations in an appropriate way.  A more successful re-integration into society with increased attendance at education and vocational training activities.  Improved vocational outcomes post-release. | If all prisoners with neurodiversity had a care plan which advised staff on how best to support them - e.g., making allowances such as showers outside of allotted association time - then this would ensure individualised support is provided and would facilitate the development of positive self-identity – Davison 2023 (full)  If prison staff are trained in how to adapt their communication to match the needs of neurodiverse prisoners, then the individual will gain more autonomy while being better supported. This will also open opportunities for learning, mutual support and being part of a community - Davison 2023 (full)  If prison staff are educated to be aware that some prisoners do not understand jargon and the details of what is required of them, and to adjust their communication accordingly, then the neurodiverse individual will feel less anxious, frustrated and embarrassed. They will also better understand and comply with the conditions of their license, be less likely to reoffend, and therefore less likely to be recalled to prison after release - Davison 2023 (full)  If staff were given communication guides which highlighted simple changes staff could make, then the needs of neurodiverse prisoners would be better met - Davison 2023 (full)  Consider developing links between in-prison and community support programmes for inmates with cognitive disabilities or other special needs - Rowe et al 2020 (full)  If prison staff are trained in caring for/understanding people with intellectual disabilities then they will better explain changes (e.g. establishment transfer), react to situations more appropriately (e.g. not punishing people for actions they can’t help/don't understand), and be a source of social support, leading to increased wellbeing for prisoners with intellectual disabilities – Boodle et al 2014 (full)  If those with learning difficulties are aided in finding social support in prison (taking care to manage expectations about the maintenance of these friendships once released) and upon release then they will be more supported and better able to cope - Boodle et al 2014 (full)  Prisoners with disabilities should have their mental/physical health problems addressed prior to release and be taught employable skills - Feist-Price et al 2014 (full)  'Prisoners who are registered with DHHS as having an intellectual disability are allocated a DHHS worker on release who is able to assist with securing accommodation, employment and other supports. Prisoners who have an ABI may receive no service" (from the VO Report) (Bunn 2019 – from SCoR scoping review)  The Ombudsman report found that people with cognitive impairment were likely to be significantly disadvantaged in the period following release due to difficulties complying with parole conditions and navigating a complex and fragmented system. Also said those with Acquired Brain Injury face unique challenges, not least because of the challenge of accessing assessment services and being accurately diagnosed, such that the subsequent failure to manage daily life is individualized – masking the culpability of the justice system in this process - (Bunn 2019 – from SCoR scoping review)  The skills learnt could be reinforced through appropriate and realistic education and training programmes for prisoners with TBI, which would provide them with space to develop as a person and a stepping-stone to bettering themselves on release - Linden et al 2021 (full)  Prisoners with TBI should be encouraged to set realistic post-release employment and educational goals - Linden et al 2021 (full)  Teaching of life skills could include a focus on literacy and numeracy skills - Coates 2016 (full)  The Personal Learning Plan should include a specific section directed towards an agreed employment pathway, where appropriate. The plan could be shared with key agencies and accessible on release. The plan should be informed by initial assessment, be subject to regular review, be integrated with the sentence plan, and be owned by the prisoner – Coates 2016 (full)  If correctional educators could be trained to teach people with learning disabilities how to learn how to learn, think, and solve problems on their own, then this would foster development of prisoners' self-esteem and social skills – Koo 2016 (full)  If life skills should be taught to inmates with learning disabilities, and designed with the challenges of learning disabilities in mind, then this would help them secure employment post-release – Koo 2016 (full)  If correctional educators' training could be held annually, then this would refresh best practices as new inmates with learning disabilities arrive over time - Koo 2016 (full)  If prisons give 24/7 care to those with intellectual disabilities without teaching them skills to live in the community/providing transition support, then they will not be able to cope upon release and feel a need to return to prison (to reconnect with friends made, to utilise services, to feel safe/cared for) – Boodle et al 2014 (full)  If prisoners had vocational councillors and could use staff-led, formatted manualized vocational group programs to provide education about job searching, modified to the individual prisoner, involving technique practice opportunities with a reflective discussion, then this would allow prisoners to better tailor their responses to their unique circumstances and help improve prisoner confidence and vocational outcomes post-release - LePage et al 2013 (full)  If support systems in the community were arranged for prisoners with IDs prior to their release, including with families if they are able to provide positive support, then they would be less likely to re-offend - Murphy & Barnoux 2015 (full)  If there could be timely planning of transition people with IDs, and good continuity of care/support, then they would be more likely to successfully reintegrate into the community after release from prison - van Dooren et al 2016 (full)  If there could be gradual release programs (for a day or two at a time building up to full release), then they would be more likely to successfully reintegrate into the community after release from prison. For some, this may require a period of live-in support - van Dooren et al 2016 (full) |

| **6. Other – unconsolidated** |
| --- |
| If social care needs of older prisoners are targeted using a cohesive framework of appropriate screening, specialist services, and a multi-disciplinary approach, then identification, assessment and support of older prisoners will be improved - Brooke et al 2020 (full)  If a national strategy was developed for the care of older prisoners in UK prisons, then better care will be provided - Brooke et al 2020b (full)  If an overarching framework was created for older people in prison, then these people could be given more adequate care - Tinker et al 2014 (full)  If adaptations could be made to the standard prison regime to address the specific needs of those with social care needs whilst maintaining their positive influence on the wider population, then the needs of prisoners with social care needs could be met while ensuring the usual running of the prison - Lee et al 2016 (full)  Poor access to social care to support ADLs may result in deteriorating health, therefore increasing the need for secondary care – Spiers et al 2019 (community)  Consider an older prisoner clinic – O’Hara et al 2015 (full)  If prisoners with social care needs were given the option to be in a prison further from home if it was better able to meet their needs, then this would enable prisoners to be more independent to get out of their cell, to be more active, and to socialise more - CLINKS & RECOOP 2021 (full)  (From a sample of people with known/suspected social care needs): 47% of participants said they had difficulty with personal care e.g. showering, using the toilet, shaving, washing, dressing or undressing. The most common ADL that people assessed reported difficulty with was washing (over 50 people) – Scottish Prison Service 2017 (full) *from SCoR review*  Common unmet needs included daytime activities (29%); benefits (28%), food (22%) and physical health (21%). Only 1% had no needs (met or unmet) at all – O’Hara et al 2016 (full) *from SCoR review*  Alternatives to custody  if prisoners’ social care needs cannot be adequately met in the prison setting, then alternatives to custody could be considered which would allow them, where possible, to serve their sentence in the community - CLINKS & RECOOP 2021 (full)  “17.1 There should be wider discussion about using community alternatives to prison for people with particularly high level care needs.  17.2 Effective multi-disciplinary work is required to introduce community alternatives to prison and to ensure that risk is fully incorporated into care planning and that Courts and the Parole Board are confident in the approaches recommended. – Levy et al 2018  ROTL is a key part of preparing people for release and in particular, people who have spent long periods in custody, but that its use has fallen significantly in recent years. Quote HM Chief Inspector of Prisons as saying that: For prisoners who are coming to the end of longer sentences for serious offences, ROTL, properly managed, contributes to their acclimatisation to life beyond prison walls and tests their readiness to live in the community without reoffending - Cornish et al 2016 (full)  Costs  If, for older prisoners with SC needs, the use of prison personnel was tailored (e.g., less security staff as lower risk of abscondment and violence, and more health and social care staff), then costs for this group would be reduced both during and after their time in prison - Kenkmann et al 2022 (scoping)  Wider implications  If prisoners are unable to access social care services within prison, then this may be breaching international obligations entered into by the United Kingdom. It may also breach the European Convention of Human Rights – Williams 2013 (full)  “The European Convention on Human Rights 1950 and the UN Convention on the Rights of Persons with Disabilities 2006, in tandem with the Equality Act 2010 (UK), should be used as overarching frameworks guiding the development of social care in prisons.” – Levy et al 2018  If prisoners with disabilities are placed in administrative segregation solely because handicap-accessible cells were not available, then this would be a violation of the Equality Act 2010 and the United Nations (UN) Convention on disability rights – Brodheim 2015 (full)  “Our findings suggest that the aging in prison crisis and conditions of confinement are human rights and intergenerational family justice issues that violate older adults in prison, their rights to dignity and respect and their access to political, civil, economic, social and cultural resources." – Maschi et al 2015 (full) |

* The referenced sources provided the insights for the statements. In some cases, original wording was retained and in others it was adapted. ‘Scoping’ indicates statement was generated during initial scoping exercise; ‘full’ indicates statement informed by, or ‘nugget’ of information taken from, the full search of the prison literature; ‘community’ indicates statement/nugget of information came from the community literature; ‘SCoR’ indicates information came from studies in the social care on release scoping review (currently under peer review) that were not identified in the current review

**Complete list of references/documents/sources of information used to inform the IPT**

Adorjan M, Chui WH. Aging Out of Crime: Resettlement Challenges Facing Male Ex-Prisoners in Hong Kong. The Prison Journal 2014;94(1):97–117.doi:10.1177/0032885513512095

Albertie A, Bourey C, Stephenson R, et al. Connectivity, prison environment and mental health among first-time male inmates in Mexico City. Glob Public Health 2017;12(2):170-184.doi:10.1080/17441692.2015.1091023

Age UK. Older Prisoners (England and Wales). Policy Position paper. 2019. Available at https://www.ageuk.org.uk/globalassets/age-uk/documents/policy-positions/care-and-support/ppp_older_prisoners_en_wa.pdf

Age UK. Day Centres. 2022. https://www.ageuk.org.uk/services/in-your-area/day-centres/ Last accessed 20 Oct 2022

Anderson I. A report on the findings of the ADASS survey of social care activity in prisons and approved premises: quarter 1 2015/16. 2015. https://www.adass.org.uk/media/4231/an-analysis-of-social-care-activity-in-prisons-and-approved-premises.pdf Last accessed 17 Oct 2022

Baidawi S, Trotter C, Flynn C. Prison experiences and psychological distress among older Inmates. Journal of Gerontological Social Work 2016;59(3):252-270.doi:10.1080/01634372.2016.1197353

Baloch NA, Jennings WG. Examining Vocational Rehabilitation Services Provided to Incarcerated Persons With Disabilities. Criminal Justice Policy Review 2021;32(3):268–283.doi.org/10.1177/0887403420913621

Baloch NA, Jennings WG. Offender vocational rehabilitation services and postrelease employment: A case for inmates with disabilities. J Offender Rehabil 2018;57(6):402-414.doi:10.1080/10509674.2018.1510865

Barry LC, Wakefield DB, Trestman RL, et al. Disability in prison activities of daily living and likelihood of depression and suicidal ideation in older prisoners. Int J Geriatr Psychiatry 2017;32(10):1141-1149.doi: 10.1002/gps.4578

Barry LC, Trestman RL, Wakefield DB, et al. Disability in prison activities of daily living and suicidal ideation in older inmates. Am J Geriatric Psychiatry 2015; 23(3 SUPPL. 1):S156-S157 (conference publication)

Bartlett A, Walker A, Harty M, et al. Health and social care services for women offenders: current provision and a future model of care. J Forens Psychiatry Psychol 2014;25(6):625-635.doi:10.1080/14789949.2014.944202

Boodle A, Ellem K, Chenoweth L. Anna's story of life in prison. Br J Learn Disabil 2014;42:117-124.doi.org/10.1111/bld.12015

Boucher NA, Van Houtven CH, Dawson WD. Older Adults Post-Incarceration: Restructuring Long-term Services and Supports in the Time of COVID-19. J Am Med Dir Assoc 2021,22(3):504-509.

Bradley A. Viewing Her Majesty’s Prison Service through a Trauma-Informed Lens. Prison Service Journal 2021;255.

Briggs AM, Valentijn PP, Thiyagarajan JA, et al. Elements of integrated care approaches for older people: a review of reviews. BMJ Open 2018;8(4):e021194.doi: 10.1136/bmjopen-2017-021194

Brodheim M. California Prison Officials Ordered to Provide Qualified Sign Language Interpreters for All Deaf Prisoners. Prison Legal News 2015. Available at https://www.prisonlegalnews.org/news/2015/jul/7/california-prison-officials-ordered-provide-qualified-sign-language-interpreters-all-deaf-prisoners/

Brooke J, Rybacka M. Development of a Dementia Education Workshop for Prison Staff, Prisoners, and Health and Social Care Professionals to Enable Them to Support Prisoners With Dementia. J Correct Health Care 2020,26(2):159-167.doi:10.1177/1078345820916444

Brooke J, Diaz-Gil A, Jackson D. The impact of dementia in the prison setting: A systematic review. Dementia 2020;19:1509-1531.doi:10.1177/1471301218801715

Brooke J. Prison initiatives to support older prisoners and those with dementia: the prisoner’s lived experience. Alzheimer's Association International Conference 2019. Los Angeles, United States. 15(7 Supplement.p1165)

Brooke J, Jackson D. An exploration of the support provided by prison staff, education, health and social care professionals, and prisoners for prisoners with dementia. J Forens Psychiatry Psychol 2019;30(5):807-823.doi:10.1080/14789949.2019.1638959

Bunn, R. (2019). Intersectional needs and reentry: Re-conceptualizing ‘multiple and complex needs’ post-release. Criminology & Criminal Justice, 19(3), 328-345. https://doi.org/10.1177/1748895817751828

Caiels J, Milne A, Beadle-Brown J. Strengths-Based Approaches in Social Work and Social Care: Reviewing the Evidence. J Long Term Care 2021;401–422.doi:10.31389/jltc.102

Care Quality Commission. The state of health care and adult social care in England 2020/21. 2021a. London: Care Quality Commission. Available at https://www.cqc.org.uk/sites/default/files/20211021_stateofcare2021_print.pdf

Care Quality Commission. Home For Good: Successful community support for people with a learning disability, a mental health need and autistic people. 2021b

https://www.cqc.org.uk/publications/themed-work/home-good-successful-community-support-people-learning-disability-mental Last accessed 27 Oct 2022

Care Quality Commission. Beyond barriers: how older people move between health and care in England. 2018. London: Care Quality Commission. Available at

https://www.cqc.org.uk/sites/default/files/20180702_beyond_barriers.pdf

Care Quality Commission. Building bridges, breaking barriers: Integrated care for older people. 2016. London: Care Quality Commission. Available at https://www.cqc.org.uk/sites/default/files/20160712b_buildingbridges_report.pdf

Care Quality Commission & HM Inspectorate of Prisons. Social care in prisons in England and Wales: a thematic report. 2018

Chadborn NH, Goodman C, Zubair M, et al. Role of comprehensive geriatric assessment in healthcare of older people in UK care homes: realist review. BMJ Open 2019;9:e026921.doi:10.1136/bmjopen-2018-026921

Clinks & RECOOP. Understanding the needs and experiences of older people in prison. 2021. London: Clinks. https://www.clinks.org/sites/default/files/2021-11/Understanding%20the%20needs%20and%20experiences%20of%20older%20people%20in%20prison_1.pdf Last accessed 13 Oct 2022

Clinks. Clinks’ response to the Health and Social Care Committee inquiry into prison healthcare. The effectiveness of prisons and prison healthcare services in meeting the physical and mental health, and social care, needs of prisoners. 2018. London: Clinks. https://www.clinks.org/sites/default/files/2018-10/clinks_response_to_prison_healthcare_inquiry_june2018.pdf

Coates S. Unlocking Potential. A review of education in prison. 2016. London: Ministry of Justice. Available at https://assets.publishing.service.gov.uk/government/uploads/system/uploads/attachment_data/file/524013/education-review-report.pdf

Cochrane A, Booth A, Walker I, et al. Examining the effectiveness of Gateway-an out-of-court community-based intervention to reduce recidivism and improve the health and well-being of young adults committing low-level offences: study protocol for a randomised controlled trial. Trials 2021 Dec 19;22(1):939. doi: 10.1186/s13063-021-05905-2

Connell C, Furtado V, McKay EA, et al. How effective are interventions to improve social outcomes among offenders with personality disorder: a systematic review. BMC Psychiatry. 2017,17;17(1):368.doi:10.1186/s12888-017-1536-3

Cornish N, Edgar K, Hewson A, et al. Social care or systematic neglect? Older people on release from prison. London: Prison Reform Trust & Restore Support Network. Available at http://www.prisonreformtrust.org.uk/Portals/0/Documents/Older-prisoner-resettlement.pdf

Cutcher Z, Degenhardt L, Alati R, et al. Poor health and social outcomes for ex-prisoners with a history of mental disorder: a longitudinal study. Aust N Z J Public Health 2014;38(5):424-9.doi: 10.1111/1753-6405.12207

Davison E. 'Before My Brother Came... I Lived Off Sandwiches': Adapting the Prison Service to ensure equality for neurodiverse prisoners. Prison Service Journal 2023, 266:11-16.

Dawson S, Kunonga P, Beyer F, et al. Does health and social care provision for the community dwelling older population help to reduce unplanned secondary care, support timely discharge and improve patient well-being? A mixed method meta-review of systematic reviews [version 1; peer review: 2 approved]. F1000Research 2020;9:857.doi.org/10.12688/f1000research.25277.1

Department for Health and Social Care. Care act 2014: Care and support statutory guidance. 2018. London: Department of Health and Social Care. Retrieved from https://www.gov.uk/government/publications/care-act-statutory-guidance/

care-and-support-statutory-guidance

Department of Health. Care Act 2014 Factsheets. Factsheet 12: Prisoners and People Resident in Approved Premises. 2016. London: Department of Health and Social Care.

Di Lorito C, Völlm B, Dening T. The individual experience of ageing prisoners: systematic review and meta-synthesis through a Good Lives Model framework. Int J Geriatr Psychiatry 2018;33:252-62.doi:10.1002/gps.4762

Dillon G, Vinter LP, Winder B, et al. ‘The guy might not even be able to remember why he's here and what he's in here for and why he's locked in’: residents and prison staff experiences of living and working alongside people with dementia who are serving prison sentences for a sexual offence. Psychol Crime Law 2019;25:5,440-457,doi:10.1080/1068316X.2018.1535063

du Toit SHJ, Withall A, O'Loughlin K et al. Best care options for older prisoners with dementia: a scoping review. Int Psychogeriatr 2019;31(8):1081-1097.doi: 10.1017/S1041610219000681

Durcan G, Allan J, Hamilton IS. From prison to work: a new frontier for Individual Placement and Support. 2018. London: Centre for Mental Health.

Durcan G. Beyond the gate: supporting the employment aspirations of offenders with mental health conditions. Mental Health and Social Inclusion 2012,16(4):188-193.doi:10.1108/20428301211281041

Durr P. Trauma-informed work with people in contact with the criminal justice system. Evidence review. 2020. London: Clinks. https://www.clinks.org/publication/trauma-informed-work-people-contact-criminal-justice-system

Eadie T, Grainge P, Jackson J, et al. Good Practice Guide. Working with older prisoners. 2017. Bournemouth: RECOOP.

Ellem K, Denton Michelle, Davidson D. Supporting people with intellectual and developmental disabilities leaving prison. In Lindsay, William, Craig, Leam, & Griffiths, Dorothy (Eds.) The Wiley Handbook on What Works for Offenders with Intellectual and Developmental Disabilities: An Evidence‐Based Approach to Theory, Assessment, and Treatment. 2020. John Wiley & Sons, United States of America, pp. 263-281.

Ellen ME, Demaio P, Lange A, et al. Adult Day Center Programs and Their Associated Outcomes on Clients, Caregivers, and the Health System: A Scoping Review. The Gerontologist 2017;57(6):e85–e94.doi.org/10.1093/geront/gnw165

Ethridge G, Dowden AR, Brooks M, et al. Employment and earnings among ex-offenders with disabilities: A multivariate analysis of RSA-911 data. J Vocational Rehab 2020;52(3):279-289.doi: 10.3233/JVR-201077

Ethridge PA, White TG. The Use of Medically Recommended Intensive Supervision (Medical Parole) in Texas. J Correctional Health Care 2015;21(4):375-89.doi:10.1177/1078345815600158

Favril L, Yu R, Hawton K, Fazel S. Risk factors for self-harm in prison: a systematic review and meta-analysis. Lancet Psychiatry 2020;7(8):682-691.doi: 10.1016/S2215-0366(20)30190-5

Feist-Price S, Lavergne L, Davis, M. Disability, Race and Ex-Offender Status: The Tri-vector Challenge to Employment. J Appl Rehabil Couns 2014,45(4).doi:10.1891/0047-2220.45.4.25

Fields NL, Anderson KA, Dabelko-Schoeny H. The effectiveness of adult day services for older adults: a review of the literature from 2000 to 2011. J Appl Gerontol 2014;33(2):130-63.doi:10.1177/0733464812443308

Firth CL, Sazie E, Hedberg K, et al. Female Inmates with Diabetes: Results from Changes in a Prison Food Environment. Womens Health Issues 2015;25(6):732-8.doi:10.1016/j.whi.2015.07.009

Flanigan C. Health and Healthcare in Prison: A Literature Review. 2020. Available at https://www.scotphn.net/wp-content/uploads/2020/12/Prison-Literature-Review-Dec-2020.pdf

Flatt JD, Williams BA, Barnes D, et al. Post-traumatic stress disorder symptoms and associated health and social vulnerabilities in older jail inmates. Aging Ment Healt 2017;21(10):1106-1112.doi:10.1080/13607863.2016.1201042

Flynn S, Humber N, Bartlett, A, et al. Women offenders and mental health. In Castle D & Abel K (Eds.), Comprehensive Women's Mental Health (pp. 148-160). 2016. Cambridge: Cambridge University Press. doi:10.1017/CBO9781107045132.014

Forsyth K, Daker-White G, Archer-Power L, et al. A qualitative exploration of the older prisoner health and social care assessment and plan (OHSCAP) in a “dangerous” prison system. The Journal of Forensic Psychiatry & Psychology 2023, 34:2, 275-293, DOI: 10.1080/14789949.2023.2208571

Forsyth K, Swinson N, Archer-Power L, et al. Audit of fidelity of implementation of the older prisoner health and social care assessment and plan (OHSCAP). The Journal of Forensic Psychiatry & Psychology 2022; 33:1:21-36.doi:10.1080/14789949.2021.2008472

Forsyth K, Webb RT, Power LA, et al. The older prisoner health and social care assessment and plan (OHSCAP) versus treatment as usual: a randomised controlled trial. BMC Public Health 2021;21:2061.doi.org/10.1186/s12889-021-11965-5

Forsyth K, Heathcote L, Senior J, et al. Dementia and mild cognitive impairment in prisoners aged over 50 years in England and Wales: a mixed-methods study. Health Serv Deliv Res 2020;8(27).doi:10.3310/hsdr08270

Forsyth K, Archer-Power L, Senior J, et al. The effectiveness of the Older prisoner Health and Social Care Assessment and Plan (OHSCAP): a randomised controlled trial. Health Serv Deliv Res 2018;5(31).doi:10.3310/hsdr05310

Forsyth K, Archer-Power L, Senior J, et al. The effectiveness of the Older prisoner Health and Social Care Assessment and Plan (OHSCAP): a randomised controlled trial. 2017 Southampton (UK): NIHR Journals Library.

Forsyth K, Senior J, Stevenson C, et al. 'They just throw you out': release planning for older prisoners. Ageing Soc 2015;35:2011-2025.doi:10.1017/S0144686X14000774

Fox C, Marsh C. ‘Personalisation’: Is social innovation possible under Transforming Rehabilitation? Probation Journal 2016;63(2):169–181.doi.org/10.1177/0264550516648402

Fox A, Fox C, Marsh C. Could Personalisation Reduce Re-offending? Reflections on Potential Lessons from British Social Care Reform for the British Criminal Justice System. Journal of Social Policy 2013,42(4):721-741.doi:10.1017/S0047279413000512

Frost R, Rait G, Wheatley A, et al. What works in managing complex conditions in older people in primary and community care? A state-of-the-art review. Health Soc Care Community 2020;28:1915-1927.doi.org/10.1111/hsc.13085

García-Martínez J, Álvarez C. Analysis of penitentiary, social and legal operators' perceptions of prison inmates with intellectual disabilities. Rev Esp Sanid Penit 2021;23(3):115-118.doi:10.18176/resp.00040.

Goodman C, Davies SL, Gordon AL, et al. Optimal NHS service delivery to care homes: a realist evaluation of the features and mechanisms that support effective working for the continuing care of older people in residential settings. Health Serv Deliv Res 2017;5(29).doi.org/10.3310/hsdr05290

Grohs M. Video Visitation. Corrections Forum 2017.

Grohs M. Aging inmate care: a special report. Corrections Forum 2015.

Hagos AK, Withall A, Ginnivan NA, et al. Barriers and enablers to health and social services for older prisoners transitioning to community", International Journal of Prisoner Health 2022, 124-137. https://doi.org/10.1108/IJPH-08-2021-0088

Hagos AK, Butler TG, Howie A, et al. Optimising the Care and Management of Older Offenders: A Scoping Review. The Gerontologist 2021;62(9):e508–e519.doi.org/10.1093/geront/gnab104

Hamilton IS, Schneider J, Kane E, et al. Employment of ex-prisoners with mental health problems, a realistic evaluation protocol. BMC Psychiatry 2015:15(185).doi.org/10.1186/s12888-015-0553-3

Harley DA. Adult Ex-Offender Population and Employment: A Synthesis of the Literature on Recommendations and Best Practices. Journal of Applied Rehabilitation Counseling 2014,45(3).doi:10.1891/0047-2220.45.3.10

Hayes AJ, Burns A, Turnbull P, et al. The health and social needs of older male prisoners. Int J Geriatr Psychiatr 2012;27:1155–62.doi.org/10.1002/gps.3761

Hellman Y, Oganesyan A, Gutierrez A (Jr). Best Practices in Education for Mentally Ill Inmates: Los Angeles County Sheriff's Department's Education Based Incarceration Unit. Corrections Today 2016.

Henwood, M. Skills around the person: Implementing asset-based approaches in adult social care and end of life care. 2014. Leeds: Skills for Care.

Her Majesty’s Government, NHS England, Ministry of Justice. National partnership agreement for prison healthcare in England 2018–2021. 2018. https://assets.publishing.service.gov.uk/government/uploads/system/uploads/attachment_data/file/697130/moj-national-health-partnership-2018-2021.pdf

Her Majesty’s Inspectorate of Prisons in Scotland (HMIPS). Who cares? The lived experience of older prisoners in Scotland’s prisons. 2017. Available from: https://www.prisonsinspectoratescotland.gov.uk/sites/default/files/publication_files/

SCT03172875161.pdf

Her Majesty’s Inspectorate of Probation. Custody and Resettlement. https://www.justiceinspectorates.gov.uk/hmiprobation/research/the-evidence-base-probation/specific-types-of-delivery/custody-and-resettlement/ Last accessed 24 Oct 2022

Hollomotz A, Talbot J, Gordon E, et al. Behaviour that challenges: planning services for people with learning disabilities and/or autism who sexually offend. Briefing paper. 2018. Leeds: University of Leeds

House of Commons Justice Committee. Ageing prison population. Fifth Report of Session 2019–21. London: House of Commons. https://committees.parliament.uk/publications/2149/documents/19996/default/ Last accessed 13 Oct 2022

House of Commons Justice Committee. Older Prisoners: Fifth Report of Session 2013–14.

https://www.parliament.uk/globalassets/documents/commons-committees/justice/older-prisoners.pdf

Hughes MH, ten Bensel T. "Stuck in their ways": Examining parole officers' perceptions on guiding older offenders through the re-entry process. Am J Crim Just 2022;47:287–305.doi.org/10.1007/s12103-021-09613-0

Hwang YIJ, Ginnivan NA, Simpson PL, et al. COVID-19 and incarcerated older adults: a commentary on risk, care and early release in Australia. Int J Prison Health 2021, May 17(ahead-of-print).doi:10.1108/IJPH-10-2020-0078

Jadczak A, Makwana N, Luscombe-Marsh N, et al. Effectiveness of exercise interventions on physical function in community-dwelling frail older people: an umbrella review of systematic reviews. JBI Database of Systematic Reviews and Implementation Reports 2018;16(3):752-775.doiI:10.11124/JBISRIR-2017-003551

Jenkins R, Kadis T, Wilson J, et al. Diabetes redesign in Her Majesty's Prison Wakefield: Tackling the challenges. Diabetic Medicine 2012;29(SUPPL 1:168-169. (Conference Publication)

Joyce J and Maschi T. “In here, time stands still” The rights, needs and experiences of older people in prison. 2016. Irish Penal Reform Trust.

https://www.iprt.ie/site/assets/files/6388/iprt-older_people_in_prison_report_web.pdf

Kannenberg K, Conley M. Advancing occupational justice through street-based intervention: A case study examining strategies for increasing meaningful engagement in the face of homelessness and incarceration. Work 2020;65(2):303-310.doi:10.3233/WOR-203082

Kelly L, Harlock J, Peters M, et al. Measures for the integration of health and social care services for long-term health conditions: a systematic review of reviews. BMC Health Serv Res 2020;20:358.doi.org/10.1186/s12913-020-05206-5

Kenemore TK (2014). Social Work Practice with Reentry from Incarceration. In: Rosenberger, J. (eds) Relational Social Work Practice with Diverse Populations. Essential Clinical Social Work Series; 239-260. Springer, New York, NY. https://doi.org/10.1007/978-1-4614-6681-9_15

Kenkmann A, Ghanem C, Erhard S. The Fragmented Picture of Social Care for Older People in German Prisons. Journal of Aging & Social Policy 2022.doi: 10.1080/08959420.2022.2031701

Koo A. Correctional education can make a greater impact on recidivism by supporting adult inmates with learning disabilities. Journal of Criminal Law & Criminology 2016. Accessed at https://core.ac.uk/download/pdf/231044413.pdf

Kirst M, Im J, Burns T, et al. What works in implementation of integrated care programs for older adults with complex needs? A realist review. International Journal for Quality in Health Care 2017;29(5):612–624.doi.org/10.1093/intqhc/mzx095

Krammer S, Maercker A, Grosse Holtforth M, et al. ICD-11 posttraumatic stress disorder (PTSD) in male prisoners. Fortschr Neurol Psychiatr 2019;87:112-120.doi:10.1055/s-0044-101545

Lares LA. Psychosocial needs of released long-term incarcerated older adults. 2020. Dissertation, retrieved from https://www.proquest.com/dissertations-theses/psychosocial-needs-released-long-term/docview/2572615402/se-2

Lee C, Treacy S, Haggith A, et al. A systematic integrative review of programmes addressing the social care needs of older prisoners. Health Justice 2019;7(1):9.doi:10.1186/s40352-019-0090-0

Lee C, Haggith A, Mann N, et al. Older prisoners and the Care Act 2014: An examination of policy, practice and models of social care delivery. Prison Serv J 2016;224:35-41

Lennox C, Stevenson R, Owens C, et al. Using multiple case studies of health and justice services to inform the development of a new complex intervention for prison-leavers with common mental health problems (Engager). Health Justice 2021;9(1):6.doi:10.1186/s40352-021-00131-z

LePage JP, Lewis AA, Washington EL, et al. Effects of structured vocational services in ex-offender veterans with mental illness: 6-month follow-up. J Rehabil Res Dev 2013;50(2):183-92.doi: 10.1682/jrrd.2011.09.0163

Levy S, Campbell FK, Kelly L et al. A New Vision for Social Care in Prison. 2018. Dundee: School of Education & Social Work, University of Dundee.

Linden M, O’Rourke C, Monaghan C, et al. Experiences of offenders with traumatic brain injury. Brain Impair 2021,22(1):108-123.doi:10.1017/BrImp.2020.23

Loeb SJ, Hollenbeak CS, Penrod J, et al. Care and companionship in an isolating environment: inmates attending to dying peers. J Forensic Nurs 2013;9(1):35-44.doi: 10.1097/JFN.0b013e31827a585c

Lorber DL, Chavez RS, Dorman J, et al. Diabetes management in correctional institutions. Diabetes Care 2013;36(Suppl 1):S86-92.doi:10.2337/dc13-S086

Mackie S, Darvill A. Factors enabling implementation of integrated health and social care: a systematic review. Br J Community Nurs 2016;21(2):82-7.doi: 10.12968/bjcn.2016.21.2.82

Malik N, Facer-Irwin E, Dickson H, et al. The Effectiveness of Trauma-Focused Interventions in Prison Settings: A Systematic Review and Meta-Analysis. Trauma Violence Abuse 2021;28:15248380211043890.doi:10.1177/15248380211043890

Maschi T, Viola D, Koskinen L. Trauma, stress, and coping among older adults in prison: Towards a human rights and intergenerational family justice action agenda. Traumatology 2015;21(3):188–200.doi.org/10.1037/trm0000021

Maschi T, Morgen K, Westcott K, et al. Aging, incarceration, and employment prospects: Recommendations for practice and policy reform. J Applied Rehabilitation Counseling 2014; 45(4):44–55.doi.org/10.1891/0047-2220.45.4.44

Maschi T, Viola D, Sun F. The high cost of the international aging prisoner crisis: well-being as the common denominator for action. Gerontologist 2013;53(4):543-54.doi: 10.1093/geront/gns125

McAnallen A, McGinnis E. Trauma-Informed Practice and the Criminal Justice System: A Systematic Narrative Review. Irish Probation Journal 2021;18:109-128

McCartan K.F. Trauma-informed practice. HM Inspectorate of Probation. 2020. https://www.justiceinspectorates.gov.uk/hmiprobation/wp-content/uploads/sites/5/2020/07/Academic-Insights-McCartan.pdf

McKenna B, Skipworth J, Tapsell R, et al. A prison mental health in-reach model informed by assertive community treatment principles: evaluation of its impact on planning during the pre-release period, community mental health service engagement and reoffending. Crim Behav Ment Health 2015,25: 429–439.doi:10.1002/cbm.1942

Ministry of Justice. Transforming rehabilitation: a summary of evidence on reducing reoffending. 2013. Available at: https://www.gov.uk/government/publications/transforming-rehabilitation-a-summary-of-evidence-on-reducing-reoffending

Mistry P, Muhammad L. Dementia in the incarcerated ready or not? Corrections Forum 2015;24(5),8-12.

Mohan ARM, Thomson P, Leslie SJ, et al. A Systematic Review of Interventions to Improve Health Factors or Behaviors of the Cardiovascular Health of Prisoners During Incarceration. J Cardiovasc Nurs 2018;33(1):72-81.doi:10.1097/JCN.0000000000000420

Moll A. Losing Track of Time: Dementia and the Ageing Prison Population: Treatment Challenges and Examples of Good Practice. 2013. London, UK: Mental Health Foundation

Mulcahy J. Towards ACE-Aware, trauma responsive penal policy and practice. Prison Service Journal 2019;245:3-13.

Munday D, Leaman J, E O’Moore. Health and social care needs assessments of the older prison population: a guidance document. London: Public Health England.

Murphy GH, Barnoux M, Blake E, et al. The costs and benefits of social care support with ex-offenders with intellectual difficulties. Journal of Intellectual Disability Research 2015;59(SUPPL. 1):66-67). Conference: 10th International Congress of the Eamhid. Florence Italy.

Naessens L. Addressing the needs of people in prison: the case of prison work. Eur J Social Work 2020,23:6, 933-944.doi: 10.1080/13691457.2020.1805586

National Institute of Clinical Excellence (NICE). Integrated health and social care for people experiencing homelessness. NICE guideline [NG214] 2022. Available at https://www.nice.org.uk/guidance/ng214/chapter/Recommendations

National Institute of Clinical Excellence (NICE). Evidence for strengths and asset-based outcomes. A quick guide for social workers. 2019. London: National Institute for Health and Care Excellence. Available at https://www.nice.org.uk/Media/Default/About/NICE-Communities/Social-care/quick-guides/strengths-and-asset-based-outcomes-quick-guide.pdf

National Institute of Clinical Excellence (NICE). People's experience in adult social care services: improving the experience of care and support for people using adult social care services. 2018a. London: National Institute for Health and Care Excellence. Available at https://www.nice.org.uk/guidance/ng86/resources/peoples-experience-in-adult-social-care-services-improving-the-experience-of-care-and-support-for-people-using-adult-social-care-services-pdf-1837698053317

National Institute of Clinical Excellence (NICE). Care and support of people growing older with learning disabilities. 2018b. London: National Institute for Health and Care Excellence. Available from https://www.nice.org.uk/guidance/ng96

National Institute of Clinical Excellence (NICE). Physical health of people in prisons. Quality standard [QS156]. 2017. London: National Institute for Health and Care Excellence. Available from https://www.nice.org.uk/guidance/qs156/chapter/Quality-statements

National Institute of Clinical Excellence (NICE). Physical health of people in prison. NICE guideline [NG57]. 2016. London: National Institute for Health and Care Excellence. Available at: https://www.nice.org.uk/guidance/ng57

National Institute of Clinical Excellence (NICE). Home care: delivering personal care and practical support to older people living in their own homes. 2015. Available at: https://www.nice.org.uk/guidance/ng21

National Institute for Health Research (NIHR). Advancing Care - Research with care homes. Themed Review. 2017. Southampton: University of Southampton; NIHR Dissemination Centre. doi:10.3310/themedreview-001931

National Offender Management Service. Adult Social Care. 2016.

https://www.gov.uk/government/publications/adult-social-care-psi-032016-pi-062016

Her Majesty’s Prison and Probation Service & National Probation Service. National Probation Service Health and Social Care Strategy 2019-2022. 2019. London: National Probation Service

NHS England and NHS Improvement. Meeting the healthcare needs of adults with a learning disability and autistic adults in prison. Guidance on the design and delivery of prison healthcare for adults with a learning disability and autistic adults accessing healthcare services whilst in prison. Version 1. 2021. https://www.england.nhs.uk/wp-content/uploads/2021/09/B0707-meeting-the-healthcare-needs-of-adults-with-a-learning-disability-and-autistic-adults-in-prison.pdf

O'Hara K, Forsyth K, Webb R, et al. Links between depressive symptoms and unmet health and social care needs among older prisoners. Age Ageing 2016;45(1):158-63.doi:10.1093/ageing/afv171

O’Hara K, Forsyth K, Senior J, et al. ‘Social Services will not touch us with a barge pole’: social care provision for older prisoners. J Forensic Psychol Res Pract 2015;26:2,275-281.doi:10.1080/14789949.2014.1000938

O’Hara M. A care practitioner’s perspective on the need for reflective practice in the work of prison officers, social care practitioners and gardaí in the Republic of Ireland. Reflective Practice 2012;13(1):39-53.doi:10.1080/14623943.2011.616886

Ogletree A, Mangrum R, Barry R. AHRQ Omissions of Care in Nursing Homes: Final Environmental Scan Report. (Prepared by American Institutes for Research under contract number HHSP233201500014I-HHSP23337003T). Rockville, MD: Agency for Healthcare Research and Quality; October 2019.

https://www.ahrq.gov/sites/default/files/wysiwyg/patient-safety/omissionsofcare-envscan.pdf

Orellana K, Manthorpe J, Tinker A. Day centres for older people: A systematically conducted scoping review of literature about their benefits, purposes and how they are perceived. Ageing & Society 2020a;40(1):73-104.doi:10.1017/S0144686X18000843

Orellana K, Manthorpe J, Tinker, A. Day centres for older people - attender characteristics, access routes and outcomes of regular attendance: findings of exploratory mixed methods case study research. BMC Geriatr 2020b;20:158.doi.org/10.1186/s12877-020-01529-4

Pasma AJ, van Ginneken EFJC, Palmen H, Nieuwbeerta P. Do Prisoners With Reintegration Needs Receive Relevant Professional Assistance? Int J Offender Ther Comp Criminol. 2023 Feb;67(2-3):247-269. doi: 10.1177/0306624X221086554

Peacock S, Burles M, Hodson A, et al. Older persons with dementia in prison: an integrative review. Int J Prison Health 2019;16(1):1-16.doi:10.1108/IJPH-01-2019-0007

Pearmain H. Care Act 2014: guidance for occupational therapists: transitions; custodial settings; employment, training and education. 2016. London: College of Occupational Therapists Ltd. https://www.rcot.co.uk/practice-resources/rcot-publications/downloads/care-act-2014-transitions Last accessed 17 Oct 2022

Pearsall A, Edge D, Doyle M, et al. Mind the gap: Improving transitions for mentally disordered offenders leaving custodial environments. Int J Psychosocial Rehabilitation 2014;18(2):101-112.

Perry AE, Waterman MG, Dale V, et al. The effect of a peer-led problem-support mentor intervention on self-harm and violence in prison: An interrupted time series analysis using routinely collected prison data. EClinicalMedicine 2021;32.doi:10.1016/j.eclinm.2020.100702

Petrillo M and Bradley A. Working with trauma in adult probation. Research & Analysis Bulletin 2022/02. 2022. Manchester: HM Inspectorate of Probation.

Pettus C, Renn T, Tripodi S, et al. Study protocol paper for the multisite randomized controlled trial of comprehensive trauma informed re-entry services for moderate to high-risk young males releasing from state prisons. Contemp Clin Trials 2022;117:106766.doi:10.1016/j.cct.2022.106766

Poveda-Moral S, Falcó-Pegueroles A, Ballesteros-Silva MP, et al. Barriers to Advance Care Planning Implementation in Health care: An Umbrella Review with Implications for Evidence-Based Practice. Worldviews Evid Based Nurs 2021;18(5):254-263.doi: 10.1111/wvn.12530

Prison Reform Trust, Centre for Mental Health, Adult Directors of Social Services & Revolving Doors. (2013). Making the difference: The role of adult social care services in supporting vulnerable offenders. London: Prison Reform Trust. Retrieved from http://www.prisonreformtrust.org.uk/wp-content/uploads/old_files/Documents/Making%20the%20difference.pdf

Prisons and Probation Ombudsman. Learning lessons bulletin. Fatal incidents investigations. Dementia. 2016. Issue 11. https://s3-eu-west-2.amazonaws.com/ppo-prod-storage-1g9rkhjhkjmgw/uploads/2016/07/PPO-Learning-Lessons-Bulletins_fatal-incident-investigations_issue-11_Dementia_WEB_Final.pdf

Psick Z, Simon J, Brown R et al. Older and incarcerated: policy implications of aging prison populations. International Journal of Prisoner Health 2017;13(1), 57–63. doi:10.1108/IJPH-09-2016-0053

Raghavan V. Social work intervention in criminal justice: Field-theory linkage. In: Singh, Shweta [Ed]. Social work and social development: Perspectives from India and the United States. 2013. p.265-289. 2013. Chicago, IL, US: Lyceum Books; US.

Robinson L, Tucker S, Hargreaves C, et al. Providing Social Care following Release from Prison: Emerging Practice Arrangements Further to the Introduction of the 2014 Care Act, The British Journal of Social Work, Volume 52, Issue 2, March 2022, Pages 982–1002, https://doi.org/10.1093/bjsw/bcab082

Robinson GE, Cryst S. Academy of Nutrition and Dietetics: Revised 2018 Standards of Practice and Standards of Professional Performance for Registered Dietitian Nutritionists (Competent, Proficient, and Expert) in Post-Acute and Long-Term Care Nutrition. J Acad Nutr Diet 2018;118(9):1747-1760.e53.doi:10.1016/j.jand.2018.06.007

Rodriguez A. Through the looking glass: Integrating male prisoners' perceptions and views of death. Dissertation. 2014 https://ir.ua.edu/handle/123456789/1756

Rowe S, Dowse L, Newton D, et al. Addressing education, training, and employment supports for prisoners with cognitive disability: Insights from an Australian programme. J Policy Pract Intellect Disabil 2020;17(1):43-50

Saunders L. Older offenders: the challenge of providing services to those aging in prison. Prison Service Journal, 2013;208:43-48

Scottish Government. Understanding the health needs of Scotland's prison population: a synthesis report. 2022.

https://www.gov.scot/publications/understanding-health-needs-scotlands-prison-population-synthesis-report/documents/

Scottish Government. Understanding the Social Care Support Needs of Scotland’s Prison Population. 2021. https://www.gov.scot/publications/understanding-social-care-support-needs-scotlands-prison-population/

Scottish Prison Service. Evaluation of the SPS Throughcare Support Service. 2017. Edinburgh: Scottish Prison Service.

https://www.sps.gov.uk/Corporate/Publications/Publication-5246.aspx

Senior J, Forsyth K, Walsh E, et al. Health and social care services for older male adults in prison: the identification of current service provision and piloting of an assessment and care planning model. 2013. Southampton (UK): NIHR Journals Library. doi: 10.3310/hsdr01050

Shaw J, Conover S, Herman D, et al. Critical time Intervention for Severely mentally ill Prisoners (CrISP): a randomised controlled trial. Southampton (UK): NIHR Journals Library; 2017 Feb.

Skarupski KA, Gross A, Schrack JA, et al. The Health of America's Aging Prison Population. Epidemiol Rev 2018;40(1):157-165.doi:10.1093/epirev/mxx020

Slasberg C, Beresford P. Strengths-based practice: social care’s latest Elixir or the next false dawn? Disabil Soc 2017;32(2):269-273.doi:10.1080/09687599.2017.1281974

Spiers G, Matthews FE, Moffatt S, et al. Impact of social care supply on healthcare utilisation by older adults: a systematic review and meta-analysis. Age Ageing 2019,48(1):57–66.doi:10.1093/ageing/afy147

Stewart W (2018) What Does the Implementation of Peer Care Training in a U.K. Prison Reveal About Prisoner Engagement in Peer Caregiving? J Forensic Nurs 2018,14(1):18-26.doi:10.1097/JFN.0000000000000183

Sumner A. Assessment and management of older prisoners. Nursing Older People 2012;24(3):16-21

Sweeney A. Evidence-Based Guidelines for Conducting Trauma-Informed Talking Therapy Assessments. 2021. Available at https://www.kcl.ac.uk/ioppn/assets/trauma-informed-assessment-guidelines.pdf

Tinker A, Gilani N, Luthra I, et al. Why is it important to consider so-called “invisible” older people in UK healthcare? Qual Ageing Older Adults 2014,15(4):187-196.doi:10.1108/QAOA-08-2014-0012

Trivedi D, Goodman C, Gage H, et al. The effectiveness of inter-professional working for older people living in the community: a systematic review. Health Soc Care Community 2013;21(2):113-28.doi:10.1111/j.1365-2524.2012.01067.x

Trotter C, Baidawi S. Older prisoners: Challenges for inmates and prison management. Australian & New Zealand Journal of Criminology 2015,48(2):200–218.doi:10.1177/0004865814530731

Tucker S, Hargreaves C, Cattermull M, et al. The nature and extent of prisoners’ social care needs: Do older prisoners require a different service response? Journal of Social Work 2021;21(3):310–328.doi.org/10.1177/1468017319890077

Tucker S, Hargreaves C, Roberts A, et al. Social care in prison: Emerging practice arrangements consequent upon the introduction of the 2014 Care Act. Br J Soc Work 2018;48:1627–1644

Turner M, Peacock M, Payne S, et al. Ageing and dying in the contemporary neoliberal prison system: Exploring the 'double burden' for older prisoners. Soc Sci Med 2018;212:161-167.doi:10.1016/j.socscimed.2018.07.009

Turner M, Peacock M. Palliative care in UK prisons: practical and emotional challenges for custodial staff, healthcare professionals and fellow prisoners. J Correct Health Care 2017;23:56-65.doi.org/10.1177/1078345816684847

Turner M, Peacock M, Varey S, et al. The healthcare needs of "end of lifers": Results of a survey of older prisoners. Palliative Medicine. Conference: 11th Palliative Care Congress. 2016. Glasgow United Kingdom;30(4):S6-S7.

Valmaggia L, Jarrett M, Campbell C, et al. Development and implementation of the London Early detection And Prevention (LEAP) service in an inner London prison setting. Early Intervention in Psychiatry. Conference: 9th International Conference on Early Psychosis - To the New Horizon. 2014. Tokyo Japan. Conference Publication: 8(SUPPL. 1):19

van Dooren K, Young JT, Claudio F, et al. Understanding the transition out of prison for people with intellectual disability. Report to the Criminology Research Advisory Council. 2016. https://www.aic.gov.au/sites/default/files/2020-05/26-1314-FinalReport.pdf

Walsh E, Forsyth K, Senior J, et al. Undertaking action research in prison: Developing the Older prisoner Health and Social Care Assessment and Plan. Action Res 2014,12(2):136–150.doi:10.1177/1476750314524006

Walton H, Harshfield A, Tomini SM, et al. Innovations in Adult Social Care and Social Work Report. November 2019. Available at https://www.nuffieldtrust.org.uk/files/2019-11/adult-social-care-innovations-horizon-scanning-report-final-13112019.pdf

Wangmo T, Handtke V, Bretschneider W, et al. Improving the Health of Older Prisoners: Nutrition and Exercise in Correctional Institutions. J Correct Health Care 2018;24(4):352-364.doi:10.1177/1078345818793121

Welsh Parliament Health, Social Care and Sport Committee. Health and social care provision in the adult prison estate in Wales. 2021. Available at https://senedd.wales/media/ct4f03nb/cr-ld14318-e.pdf

Williams J. Social care and older prisoners. Journal of Social Work 2013;13:471–491.doi: 10.1177/1468017311434886

Winters S, Magalhaes L, Kinsella EA, et al. Cross-sector Service Provision in Health and Social Care: An Umbrella Review. Int J Integr Care 2016;16(1):10.doi:10.5334/ijic.2460

World Health Organization. Organizational Models of Prison Health. Considerations for Better Governance. 2020. Copenhagen: WHO Regional Office for Europe. https://apps.who.int/iris/bitstream/handle/10665/336214/WHO-EURO-2020-1268-41018-55685-eng.pdf

World Health Organization. Prisons and Health. 2014. <https://www.who.int/europe/publications/i/item/9789289050593>

Wright L. “You’ve Made My Mirror Smile at Me” – Compassion and Complex Trauma in Prison. The Royal College of Psychiatrists Quality Network for Prison Mental Health Services newsletter 2021,12. https://www.rcpsych.ac.uk/docs/default-source/improving-care/ccqi/quality-networks/prison-quality-network-prison/newsletters/qnpmhs---newsletter-edition-12.pdf?sfvrsn=7ea319a3_2

Young J, van Dooren K, Claudio F, et al. Transition from prison for people with intellectual disability: A qualitative study of service professionals. Trends & issues in crime and criminal justice no. 528. 2016. Canberra: Australian Institute of Criminology. https://www.aic.gov.au/publications/tandi/tandi528.

Zubala A, MacGillivray S, Frost H, et al. Promotion of physical activity interventions for community dwelling older adults: A systematic review of reviews. PLoS One 2017;12(7):e0180902.doi: 10.1371/journal.pone.0180902
