# Supplementary material for: Developing an initial programme theory for a model of social care in prisons and on release (empowered together): A realist synthesis approach
Source: Med Sci Law. 2024 Jul 25;65(3):194–206. doi: 10.1177/00258024241264762 (PMC12149453; doi:10.1177/00258024241264762)
Supplement: sj-docx-7-msl-10.1177_00258024241264762 - Supplemental material for Developing an initial programme theory for a model of social care in prisons and on release (empowered together): A realist synthesis approach [file sj-docx-7-msl-10.1177_00258024241264762.docx]

**Extended Findings section**

This section presents a narrative summary of the four consolidated CMOs, which are outlined in Table 2 and visually in Supplementary Figure S2. Thus, this section encompasses a description of the model of social care that our expert by experience group has named ‘Empowered Together’. The content of the CMOs is based on evidence and insights from sources as described In the Methods. The complete list of if-then statements, CMOs, and ‘nuggets’ of information including sources of material are included in Supplementary File 1). The remainder of the Findings describes the four consolidated CMOs in detail, including expected outcomes.

**Identifying social care needs**

Before social care needs can be addressed they need to be identified, either on arrival or, for emerging needs, at any point during an individual’s stay in prison.

A key resource needed to ensure accurate and timely identification of needs is a standardised but flexible, person-centred screening tool capable of detecting ‘harder to see’ needs. This should include open-ended questions, enabling/encouraging individuals to acknowledge any difficulties they have with mobility, self-care, relationships, or other elements of social care, with social care practitioners trained to conduct the screening in a sensitive, trauma-informed way. Trauma-informed approaches are particularly important when collaborating with individuals, given the high prevalence of past trauma in this population which can severely impact emotional well-being. The Prison Reform Trust report that 27% of adult male individuals in England and Wales had suffered childhood abuse: the risk of being re-traumatised during assessments or when receiving assistance with things like personal care is significant. The identification process should include screening for mental health problems, learning difficulties, dementia, mild cognitive impairment, and trauma alongside general health and social care needs. If person-centred screening tools are used, then people will feel more comfortable, validated, and safe about acknowledging their problems when they first enter prison.

Active case finding is fundamental to ensure social care needs are identified, particularly those that develop over time and may not be apparent on arrival. This should involve repeating the formal identification process twice yearly and enabling suitably trained staff to proactively talk to individuals, asking questions about their ability to get around safely, take care of themselves, and participate in activities, with a robust route into assessment. This should involve liaison between care staff and prison officers who may identify/refer individuals with additional support needs if these become apparent.

It is important that the potential difficulties that the physical environment poses for some individuals are considered when needs are being identified. This could be, for example, the need for special equipment or adaptations to the cell such as handrails to help people feel safe and be as independently mobile as possible.

The identification of social care needs must happen within an integrated care system which includes clarity for staff about respective roles/responsibilities. Awareness of issues relating to social care, including difficulties accessing facilities and less visible problems such as loneliness or nutrition, is vital for this to work. While all individuals, their families, and staff should be educated about social care issues, staff should receive formal training to recognise/understand these needs, drawing on the tenets of equality, diversity, and inclusion. It is important that training programmes are co-designed with individuals and voluntary organisations, further promoting person-centredness and ensuring that pertinent issues are addressed. By possessing the relevant knowledge and insight, prison staff will be more responsive to individuals’ needs and confident in their role. As regards social care practitioners, while some receive training around social work in prisons, many do not feel suitably equipped or confident to work in this setting. Training, co-designed by individuals, voluntary groups, prison staff, and social care practitioners, would ensure they were better equipped and more willing to work with clients in the prison setting, and would cultivate rapport between staff and individuals, and between prison staff and practitioners. Well-advertised, accessible self-referral options should be available together with provision of user-friendly informational posters and leaflets, such as ‘The Care Act and You’ leaflet, with the option of advocacy to ensure the information is understood by the individual or, where necessary, make decisions on their behalf.

**Assessment**

Individuals should be empowered to be actively involved in their assessments and in co-designing their care plans, which must be timely and take the limitations of the physical environment into account. There should be the option of assistance from an advocate for those with insufficient capacity to meaningfully participate in their assessments/care plans. Advocates could be informal and internal to the prison (peers, prison staff, healthcare staff) with the person’s consent, or formal independent advocacy services including LAs or other staff who must be approved by the LA (e.g., the third sector). By co-designing their individualised support package, people with care needs would feel valued, motivated, and included, and would gain hope for the future. All staff should be educated about trauma-informed approaches. In response, they would be more sympathetic, display greater empathy, and be more likely to foster feelings of trust and safety within the individual, thereby facilitating rapport and reducing the risk of re-traumatisation. The assessment process itself will be traumatising for some individuals, so formal assessments should be conducted by suitably qualified practitioners.

Strengths and assets-based approaches to assessment and care planning focus on people’s capabilities, explore help available from wider support networks, and take account of issues of importance to the individual. Adopting such methods would help people explore their needs and priorities and facilitate a holistic approach by which self-defined strengths, preferences, aspirations and needs form the basis of care/support. Care needs change over time, therefore regular follow-up assessments should be conducted, with changes to care plans being jointly agreed. It is important that everyone has user-friendly information about social care rights and the option to self-refer in response to changing needs thereby encouraging them to seek help when needed.

Anticipated outcomes of TIP approaches to assessment include care plans that are responsive and tailored to the individual; greater inclusion/equality; more engagement with services, less risk of traumatisation, greater equivalence of care, and improved relationships.

**Provision of care and support**

To optimise social care in prisons it is vital that collaborative, integrated working is implemented whereby prisons, local authorities, healthcare, social care, and voluntary organisations develop joint working arrangements with multidisciplinary teams (MDTs), co-designed goals, shared aims, and agreements on information sharing. Pivotal to this is the nomination of a social care lead in each prison to facilitate the co-ordination of care. Care leads should be embedded within the integrated care system to coordinate referrals, and MDTs should be involved in care planning, but it is crucial that there is clarity regarding roles and responsibilities and that all stakeholders are accountable for the implementation of these plans, which could be expediated by co-designed MOUs (memorandums of understanding). Clarification around responsibilities would enable staff to feel more confident and organised in their role. There should be formal up-to-date agreements clarifying roles/responsibilities throughout the integrated system. Through joint working, co-designed goals, and nominated points of contact, staff would feel more satisfied and assured in their roles, with mutual support and respect being garnered. This would reduce duplication of effort while promoting person-centred approaches to addressing individuals’ needs.

Services should be co-designed with people who have social care needs and experience of living in prison. Tailoring to individual needs, this should include safe access to purposeful activities such as employment or vocational training, and fully integrated, meaningful day care support where appropriate. Support should be available to help people build/maintain relationships with family and fellow individuals. In response to an integrated, tailored approach, individuals would feel less isolated and more motivated, and would gain a sense of pride and hope. Outcomes include equal access to purposeful activity, improved prospects for self-improvement, and increased social inclusion and autonomy.

Peer support systems, in which the strengths and assets of other individuals can be cultivated by training them to support people with social care needs, are seen as a major resource. It is evident, however, that peer supporters must be suitably assessed, trained, safeguarded, and supported themselves. Where possible, the peer supporter and support receiver should be matched to take account of cultural, religious, communication and support needs. Training and support for peers could involve third-sector organisations (such as RECOOP), while group peer mentoring programmes could be run by ex-offenders who themselves had social care needs. A professional could be employed to support peer supporters who could be encouraged to obtain recognised qualifications. Peer supporters should not be involved in providing personal care. By being supported by peers, care receivers would experience greater dignity and connection to peers resulting in a more positive experience. Peer supporters would feel a sense of purpose and increased self-esteem and would benefit from new transferable skills, thereby increasing confidence, motivation, and prospects.

Individuals often find it difficult to build or maintain personal relationships with fellow individuals or family and friends. This can be particularly difficult for people with social care needs alongside other problems such as learning difficulties, mental health problems, or physical frailty. This has negative consequences for individuals while serving their sentence, such as loneliness and depression, and is a serious barrier to successful re-integration into the community. Individuals should be supported to maintain contact with family/friends, and arrangements made to encourage family visits. This is particularly important for certain groups such as people with learning difficulties or dementia. The duration/frequency of visits may be more than the standard amount if this is what individuals want/need to maintain their social network. This could include online visits/video visitations, which should not be a replacement for in-person visits. It is also important to ensure facilities within the prison are accessible to enable social interaction and development of positive relationships with fellow individuals. Accessible activities include tailored clubs and buddying systems. Positive staff relationships are important particularly for those with no other contacts. By helping individuals to build/maintain relationships they would feel less isolated and despondent, more included and valued, with a more positive outlook for the future. Their mental well-being would improve, and the risk of self-harm/suicide would diminish. They would find it easier to cope and be more likely to have a good support network on release, thereby reducing the risk of re-offending.

The physical environment of prisons is often obstructive and even dangerous to those who have mobility, vision, or self-care problems, but having accommodation in prison that meets one’s care needs is vital to an individual’s human rights, dignity, and inclusion, as is an accessible built environment that is safe to navigate. Appropriate modifications and resources may include raised toilets and chairs, showers/baths with grab rails, lower bunks, wider cell doors, and ground floor accommodation. Adapting corridors and communal areas so that they can safely accommodate those who struggle to get around due to physical or sensory problems would allow easier access to facilities and activities for which individuals who are otherwise excluded. This in turn would enable them to build and maintain social relationships and reduce the risk of loneliness and depression, while relatively low-cost adjustments would help to address needs such as toileting, personal hygiene, help with mobility, and independence. The option of dedicated wings for people with social care needs should also be considered, bearing in mind this has advantages and disadvantages.

**Social care on release from prison**

Individuals should be offered tailored pre-release planning and courses, and planning should begin as early as possible, with the individual’s engagement, to enable iterative assessment of predicted needs, and covering, at the very least, housing, training/education, employment, food provision, and social and healthcare. Individuals should be enabled to maintain or learn life skills, with other issues such as LDDs being considered. Courses should be co-designed, trauma-informed, and run by staff who have been specifically trained to do so. Employment training should focus on basic work life skills, particularly for people with disabilities, managing expectations, building up resumes/CVs and other job-specific skills training. Employment placement programmes should ideally be developed with input from employment specialists and include special arrangements with appropriate employers. Individuals should be provided with personalised guidance notes prior to release, and a care package (if applicable) and personalised pathway document upon release. A single point of contact and special ‘nodal points’ should be established for those returning to the community, providing trauma-informed probation, counselling, and aftercare services, continuing-care packages, and joined-up support including suitable housing, employment, and access to benefits. There should be arrangements in place for regular follow-up and ongoing support, and self-referral options to address changing needs.

In response to timely, tailored release planning and care packages, individuals would respond with positivity, motivation, confidence, pride, hope and dignity. Expected outcomes include development/retention of necessary skills to successfully reintegrate into the community, including securing and maintaining appropriate accommodation, paid employment, continuity of care, and reduction in fears/anxieties prior to release. This would in turn reduce the risk of deterioration in well-being, homelessness, and recidivism. Employers would be more convinced of ex-offenders’ potential, while less recidivism would have longer-term benefits for families and wider society.
